# Deciphering the Genetic Crosstalk between Microglia and Oligodendrocyte Precursor Cells during Demyelination and Remyelination Using Transcriptomic Data

**DOI:** 10.3390/ijms232314868

**Published:** 2022-11-28

**Authors:** Jennifer Enrich-Bengoa, Gemma Manich, Irene R. Dégano, Alex Perálvarez-Marín

**Affiliations:** 1 Biophysics Unit, Department of Biochemistry and Molecular Biology, School of Medicine, Universitat Autònoma de Barcelona, 08193 Cerdanyola del Vallès, Catalonia, Spain; 2 Institut de Neurociències, Universitat Autònoma de Barcelona, 08193 Cerdanyola del Vallès, Catalonia, Spain; 3 Medical Histology Unit, Department of Cell Biology, Physiology and Immunology, School of Medicine, Universitat Autònoma de Barcelona, 08193 Cerdanyola del Vallès, Catalonia, Spain; 4 Centro de Investigación Biomédica en Red of Cardiovascular Diseases (CIBERCV), Instituto de Salud Carlos III, 28029 Madrid, Spain; 5 Registre Gironí del Cor (REGICOR) Study Group, Hospital del Mar Medical Research Institute (IMIM), 08003 Barcelona, Catalonia, Spain; 6 Faculty of Medicine, University of Vic-Central University of Catalonia, 08500 Vic, Catalonia, Spain

**Keywords:** corpus callosum, cuprizone, demyelination, oligodendrocytes, microglia

## Abstract

Demyelinating disorders show impaired remyelination due to failure in the differentiation of oligodendrocyte progenitor cells (OPCs) into mature myelin-forming oligodendrocytes, a process driven by microglia–OPC crosstalk. Through conducting a transcriptomic analysis of microarray studies on the demyelination–remyelination cuprizone model and using human samples of multiple sclerosis (MS), we identified molecules involved in this crosstalk. Differentially expressed genes (DEGs) of specific regions/cell types were detected in GEO transcriptomic raw data after cuprizone treatment and in MS samples, followed by functional analysis with GO terms and WikiPathways. Additionally, microglia–OPC crosstalk between microglia ligands, OPC receptors and target genes was examined with the NicheNet model. We identified 108 and 166 DEGs in the demyelinated corpus callosum (CC) at 2 and 4 weeks of cuprizone treatment; 427 and 355 DEGs in the remyelinated (4 weeks of cuprizone treatment + 14 days of normal diet) compared to 2- and 4-week demyelinated CC; 252 DEGs in MS samples and 2730 and 12 DEGs in OPC and microglia of 4-week demyelinated CC. At this time point, we found 95 common DEGs in the CC and OPCs, and one common DEG in microglia and OPCs, mostly associated with myelin and lipid metabolism. Crosstalk analysis identified 47 microglia ligands, 43 OPC receptors and 115 OPC target genes, all differentially expressed in cuprizone-treated samples and associated with myelination. Our differential expression pipeline identified demyelination/remyelination transcriptomic biomarkers in studies using diverse platforms and cell types/tissues. Cellular crosstalk analysis yielded novel markers of microglia ligands, OPC receptors and target genes.

## 1. Introduction

Multiple sclerosis (MS) is the most common demyelinating disorder of the central nervous system (CNS), with 3.0 million patients worldwide. MS is more common in women than in men, and has a great impact on patients’ quality of life [1]. MS is considered as a multifactorial disease that is triggered by genetic predisposition and environmental factors, leading to neurological impairment and disabling symptoms [2,3]. Within MS, current treatments are disease-modifying therapies mainly focused on treating attacks, ameliorating the symptoms, and suppressing or modulating inflammation [4,5]. Therefore, a better understanding of the molecules that are involved in this pathology would be key to discovering novel therapies.

MS and most other demyelinating disorders are characterized by neuroinflammation that leads to demyelination, oligodendrocyte death and axonal injury. In the CNS, myelin is produced by myelinating oligodendrocytes that originate from oligodendrocyte precursor cells (OPCs), which can proliferate, migrate, and differentiate into these mature oligodendrocytes that are able to generate myelin. Different studies suggest that these different steps towards mature oligodendrocytes are orchestrated by microglia, astrocytes and OPCs [6,7,8,9,10,11]. In this crosstalk, both astrocytes and microglia can play a dual role, being either beneficial or detrimental for (re)myelination through interaction with oligodendrocytes. Beneficial roles of microglia promote OPC survival and maturation, foster OPC differentiation (i.e., via galectin-3), and contribute to myelin formation during development and remyelination [10,11,12,13,14]. However, the complete role of microglia in (re)myelination is poorly understood.

The objectives of this study were (i) to identify transcriptomic markers of demyelination and remyelination in CC, microglia, OPC and human MS samples using a common pipeline, and (ii) to study the microglia ligands as well as the OPC receptors and target genes involved in the microglia–OPC crosstalk in demyelination using available transcriptomic data from cuprizone demyelination–remyelination mouse model experiments.

## 2. Results

### 2.1. Identification of Demyelination and Remyelination Marker Genes in the Cuprizone Model

#### 2.1.1. Differentially Expressed Genes (DEGs) in the CC

**Figure 1:**
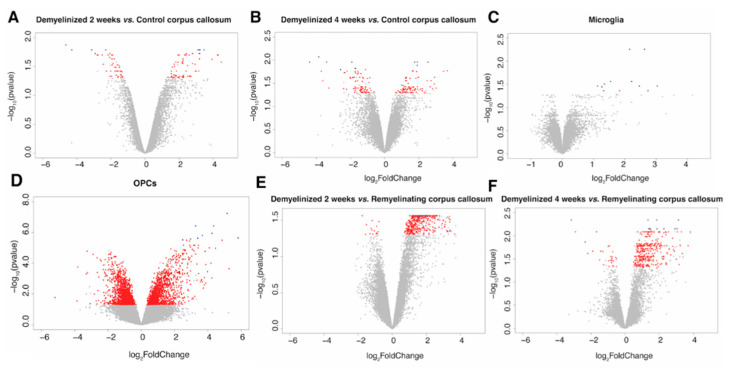
Differentially expressed genes (DEGs) in the corpus callosum (CC), oligodendrocyte progenitor cells (OPCs) and microglia. Volcano plots of −log10-adjusted *p*-values and log fold changes. Significant DEGs are shown in red and the 10 most significant DEGs are shown in blue. CC analysis at early (2-week cuprizone treatment) (**A**) and late (4-week cuprizone treatment) (**B**) demyelination. (**C**) Microglia analysis. (**D**) OPC analysis. (**E**,**F**) Analysis of early (**E**) and late (**F**) demyelination when compared to remyelinating the CC.

After 2 weeks of demyelination treatment there were 108 DEGs in the CC, 45 downregulated and 63 upregulated (Figure 1A, Table S2). The 10 most significant DEGs between 2-week cuprizone-treated mice and the control were Serpinb1a, Gdf15, Pigz, Tgm1, B230206H07Rik, Ninj2, Eif4ebp1, Slc34a3, Trib3 and Cdkn1a. Among these genes, Gdf15, Tgm1, Eif4ebp1, Trib3 and Cdkn1a were downregulated in the CC of cuprizone-treated mice, while Serpinb1a, Pigz, B230206H07Rik, Ninj2 and Slc34a3 were upregulated. There were three Wikipathways associated with the DEGs (p53 signaling, white fat cell differentiation and hypertrophy model), but no GO molecular functions.

**Figure 2:**
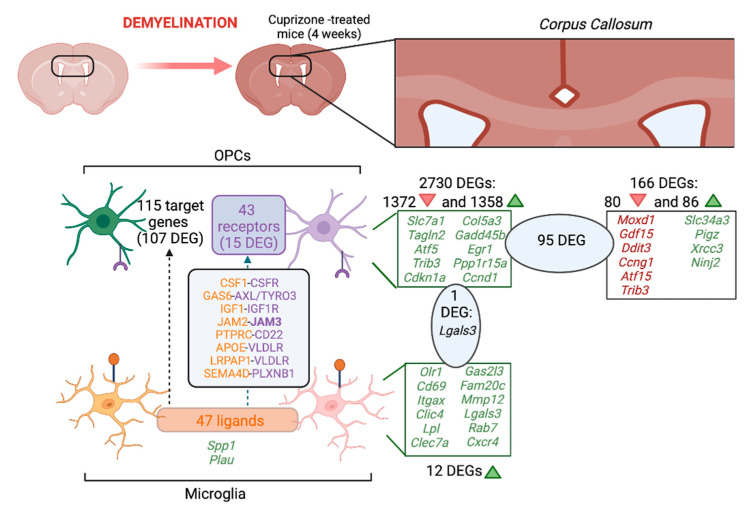
Demyelinating marker genes and microglia–OPC interactions in the cuprizone model. In this study, different datasets comparing the control to 4-week cuprizone-treated mice were analyzed in terms of the identified DEGs; upregulated genes are shown in green and downregulated genes are shown in red. In total, 95 DEGs were found in common between both CC and in OPCs, and 1 DEG was found expressed in both microglia and OPCs. In the microglia–OPC crosstalk, we found 47 ligands in microglia, 43 OPC receptors and 115 target genes in OPCs. Microglia ligands are presented in orange and OPC receptors are presented in purple. Created with Biorender.com.

**Figure 3:**
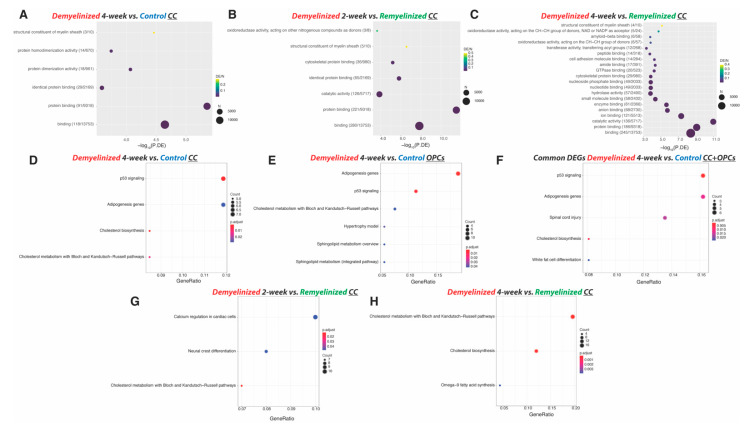
GO annotation and WikiPathways analysis of DEGs. (**A**–**C**) Dot plots including GO molecular functions associated with the 20 most significant DEGs in the CC during demyelination: (**A**) Comparison of controls to 4-week cuprizone-treated mice, and from early demyelination or late demyelination to remyelination; (**B**) 2-week-treated mice compared to remyelination; (**C**) 4-week-treated mice compared to remyelination. In these graphs, dot size shows the number of DEGs for each GO function, the -log10 of the *p*-value for over-represented significant GO terms is illustrated, and the color gradation expresses the amount of DEGs in the set divided by the total number of genes within the GO term. (**D**–**H**) Dot plots of the significantly expressed functions of WikiPathways associated with the DEGs identified in the CC (**D**) and OPCs after 4 weeks of cuprizone treatment (**E**), and with the common DEGs identified in the CC and OPC analysis (**F**). Wikipathways associated with DEGs detected in the CC at early and late demyelination compared to remyelinating CC were analyzed for (**G**) 2-week-treated mice compared to remyelination and (**H**) 4-week-treated mice compared to remyelination. In these graphs, the x-axis represents the number of DEGs in the differential expression set divided by the number of genes in the pathway. Color scale shows the adjusted *p*-value. Dot size illustrates the number of genes for each pathway.

After 4 weeks of demyelination treatment we identified 166 DEGs in the CC: 80 downregulated and 86 upregulated (Figure 1B, Table S3 and Figure S1A,B). The 10 most significant DEGs between 4-week cuprizone-treated mice and the control were *Moxd1*, *Gdf15, Ddit3, Ccng1, Atf15, Trib3, Slc34a3, Pigz, Xrcc3* and *Ninj2*. Of these, *Moxd1, Gdf15, Ddit3, Ccng1, Atf15 and Trib3* were significantly downregulated in the CC of cuprizone-treated mice, while *Slc34a3, Pigz, Xrcc3* and *Ninj2* were significantly upregulated (Figure 2). There were six significant GO molecular functions associated with the DEGs identified, such as being a structural constituent of the myelin sheath (Figure 3A). Four Wikipathways were associated with the DEGs, including cholesterol biosynthesis and metabolism (Figure 3D).

There were 75 common DEGs at 2 and 4 weeks of demyelination, all downregulated/ upregulated at both time points. Among the 10 most significant DEGs, 5 genes were present at 2 and 4 weeks: *Gdf15, Atf5* and *Trib3* were downregulated compared to control, while *Slc34a3* and *Ninj2* were upregulated.

#### 2.1.2. DEGs in Microglia

There were 12 DEGs when comparing microglia from 4-week cuprizone-treated and control mice. All DEGs were upregulated: *Olr1*, *Cd69*, *Itgax*, *Clic4*, *Lpl*, *Clec7a*, *Gas2l3*, *Fam20c*, *Mmp12*, *Lgals3*, *Rab7* and *Cxcr4* (Figure 1C, Table S4 and Figure S1C). There was one GO molecular function associated with these DEGs (carbohydrate binding), but no Wikipathways.

#### 2.1.3. DEGs in OPCs

Analysis of the DEGs of OPCs between late-demyelinated cuprizone-treated and control mice revealed 2730 DEGs (those with a *p*-value < 0.001 are presented in Table S5). Of these DEGs, 1372 were downregulated and 1358 were upregulated (Figure 1D). The 10 most significant DEGs were all upregulated: *Slc7a1*, *Tagln2*, *Atf5*, *Trib3*, *Cdkn1a*, *Col5a3*, *Gadd45b*, *Egr1*, *Ppp1r15a* and *Ccnd1* (Figure 2). There were 226 GO biological processes associated with the DEGs, such as regulation of axon extension, neurogenesis and neuron projection development. Associated WikiPathways included cholesterol and sphingolipid metabolism (Figure 3E).

#### 2.1.4. Common DEGs in the CC, Microglia and OPCs

**Figure 4:**
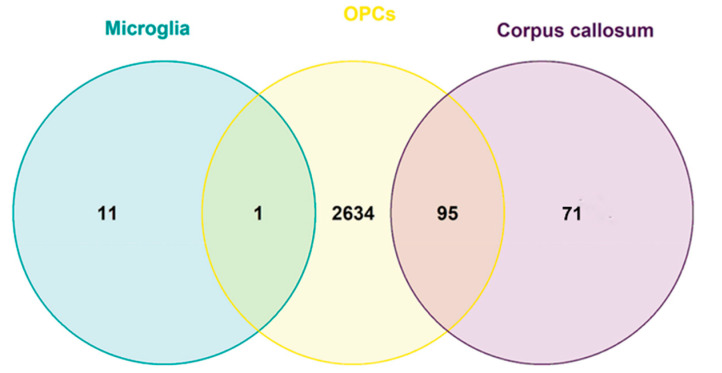
Common differentially expressed genes (DEGs) in the corpus callosum (CC), microglia and oligodendrocyte progenitor cells (OPCs) during demyelination. Venn diagram showing the number of DEGs shared between murine CC, OPC and microglia samples after a 4/5-week cuprizone treatment. No common DEGs were found between microglia, OPCs and CC, but one common DEG was found between microglia and OPCs, and 95 shared DEGs were found between CC and OPCs.

There were no DEGs in the three tissue/cell types (CC, microglia and OPCs) between 4-week cuprizone-treated and control mice. However, there was one common DEG in microglia and OPCs (*Lgals3*), and 95 common DEGs in the CC and OPCs (Table S6 and Figure 4). The 95 common DEGs were associated with 86 GO biological processes, including gliogenesis, oligodendrocyte development and nervous system development, among others. These common 95 DEGs were also associated with 5 Wikipathways, such as spinal cord injury (Figure 2F).

### 2.2. Identification of Common Demyelination Marker Genes in Multiple Sclerosis Samples and in Samples from the Cuprizone Mice Model

There were 252 DEGs when comparing MS and control samples (Table S7), and 74 of these DEGs were also identified in the cuprizone model analyses (Figure S2). Of the common 74 DEGs, 72 were differentially expressed in OPCs and 13 in the CC (Tables S8 and S9). Moreover, 11 out of 74 of the DEGs were identified in both OPC and CC samples (Table S10). One of the 11 common DEGs in MS samples and in CC and OPC samples was myelin basic protein, which is a factor in the pathogenesis of MS [15]. In addition, another of the 72 common DEGs in MS samples and in OPCs was myelin-associated oligodendrocytic basic protein.

### 2.3. Identification of Remyelination Marker Genes in the CC in the Cuprizone Model

After 2 weeks of demyelination treatment, the CC had 415 DEGs compared to the remyelinating CC, including 15 downregulated and 400 upregulated. The 10 most significant DEGs were all upregulated: *D16Ertd472e*, *Aoc1*, *Krt15*, *Serpinb1c*, *Slain1*, *Cxcl10*, *Sntn*, *Lrig3*, *Ppfibp2* and *Dusp15* (Figure 1F and Table S11). These DEGs were associated with seven GO molecular functions, related to myelin sheath structure (Figure 3B), and with three WikiPathways, including cholesterol metabolism (Figure 3G).

When comparing the CC having undergone 4 weeks of demyelination and the remyelinating CC, there were 355 DEGs, 27 were downregulated and 328 were upregulated (Figure 1F and Table S12). The 10 most significant DEGs in this case were *Ninj2*, *Mog*, *Cyp3a13*, *Trib3*, *Gm5067*, *Shgl3*, *Msmo1*, *E330037M01Rik*, *Plekhh1* and *Insig1*. From these, the only downregulated DEG was *Trib3*, while the rest were upregulated. There were 23 GO molecular functions associated with the DEGs, including being a structural constituent of the myelin sheath and amyloid-beta binding (Figure 3C and Table S13). Three WikiPathways were associated with the DEGs: cholesterol biosynthesis and metabolism, and omega-9 fatty acid synthesis (Figure 3H).

There were 159 common DEGs in the remyelinating CC compared to 2 or 4 weeks of demyelination. All were either up- or downregulated in both analyses.

### 2.4. Cell Type Composition in CC Samples

Estimated cell fractions of CC samples are presented in Table S14. The cell types showing the largest proportion in CC samples were neurons, astrocytes and oligodendrocytes, while the ones showing the lowest proportions were pericytes, endothelial cells and OPCs. Cell fraction estimation was similar between samples treated, or not treated, with cuprizone. However, in control samples, the proportion of astrocytes was higher and the proportion of oligodendrocytes was lower than in cuprizone-treated samples. There was high accuracy in the predictions, as shown by the Pearson correlation coefficient and the root mean square error (RMSE) between CC data and cell type predicted data (Table S15).

### 2.5. Microglia–Oligodendrocyte Interaction in Cuprizone-Induced Demyelination–Remyelination: Potential Receptor–Ligand Pairs to Promote Remyelination

**Figure 5:**
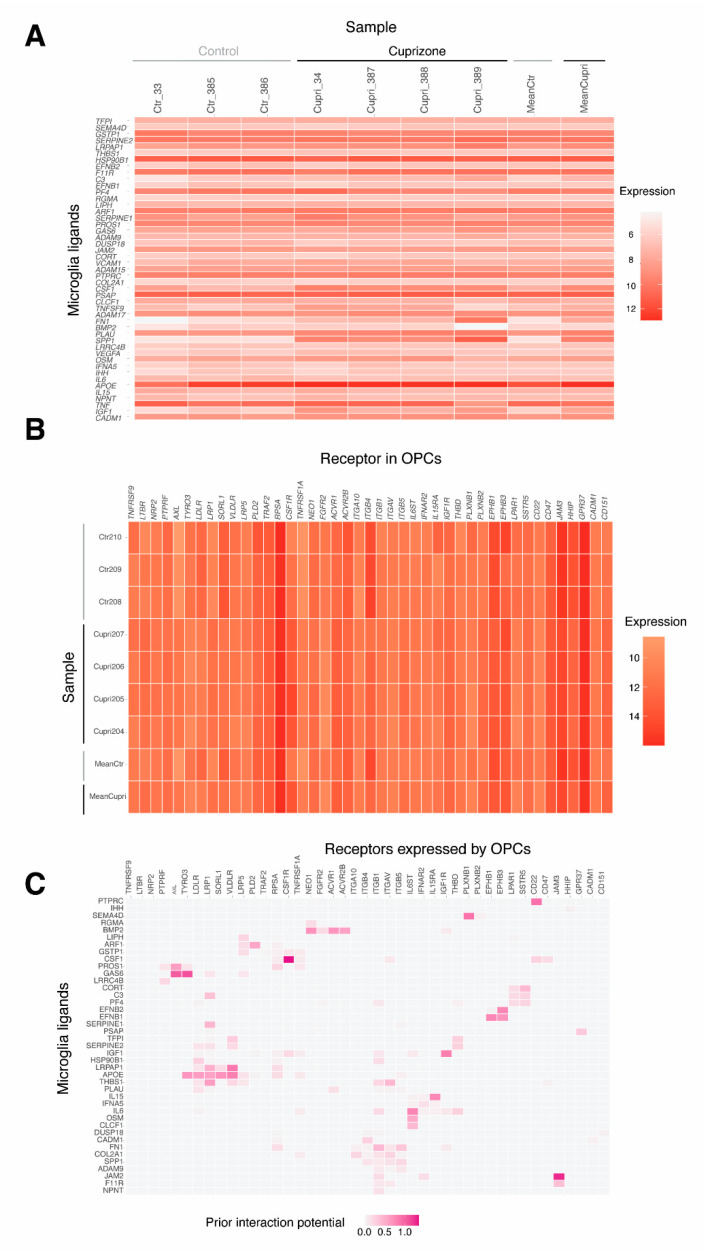
Identification of interacting microglia ligands and oligodendrocyte progenitor cell (OPC) receptors using the NicheNet model. (**A**,**B**) Expression of microglia ligands in microglia samples (**A**), and OPC receptor expression in OPC samples (**B**) from cuprizone-treated (Cupri) and control mice (Ctr). (**C**) Predicted interactions between identified microglia ligands and OPC receptors. In all figures, the color scale indicates the levels of expression, the darker the red or purple colors are, the higher the expression (red) or the regulatory potential of microglia ligands for OPC receptors (purple).

In total, 47 potential ligands were identified in microglia that showed a similar potential to regulate the OPC gene expression set of interest following cuprizone treatment (Figure 2 and Figure 5A, Table S14). All of them showed active target links in OPC genes differentially expressed after demyelination. Ligands showed similar expression in cuprizone-treated and control samples, except for the increased expression of two genes—Plau (plasminogen activator urokinase) and Spp1 (secreted phosphoprotein 1)—which showed borderline significant differences in expression (Table S15, adjusted *p*-value = 0.052).

A total of 43 potential receptors for ligands identified in OPC after cuprizone treatment were detected in a ligand–receptor analysis (Figure 2 and Figure 5B). The maximal interaction potential was discovered for the following microglia ligand–OPC receptor pairs: CSF1–CSF1R, GAS6–AXL/TYRO3, IGF1–IGF1R, JAM2–JAM3, PTPRC–CD22, APOE–VLDLR, LRPAP1–VLDLR, SEMAD4D–PLXNB1. From these, the cytokine pair CSF1–CSF1R, GAS6–AXL/TYRO3, the TGF-β signaling transductor SEMAD4D–PLXNB1, the tyrosine kinase signal receptor PTPRC (also known as CD45) and ligand CD22, and the growth factor pair IGF1–IGF1R are involved in cell migration, survival and/or phagocytosis [16,17,18,19,20,21]; integrins JAM2–JAM3 function as cell adhesion molecules [22], and in APOE–VLDLR, LRPAP1–VLDLR, the receptors are involved in lipid transport. Out of 43 receptors, 15 were differentially expressed in OPCs from cuprizone-treated compared to control mice (Table S18). From these, *Jam3* was the most differentially expressed in OPCs, being downregulated (Figure 5C).

**Figure 6:**
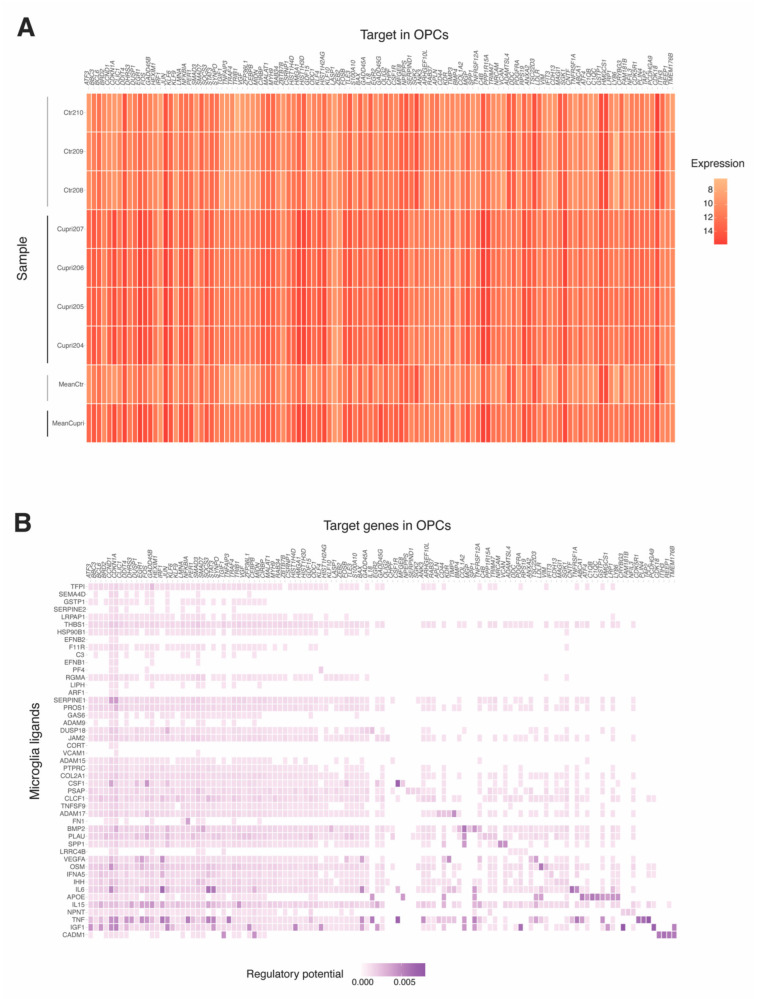
Identification of interacting microglia ligands and oligodendrocyte progenitor cell (OPC) target genes using the NicheNet model. (**A**) Expression of OPC target genes detected in samples of OPC from cuprizone-treated (Cupri) or control (Ctr) mice. (**B**) Predicted interactions between microglia ligands and OPC target genes. In all figures, the color scale indicates the levels of expression, the darker the red or purple colors are, the higher is the expression (red) or the regulatory potential of microglia ligands for OPC target genes (purple).

Overall, 115 DEGs that could be predicted targets of the identified ligands were identified in OPCs (Figure 2 and Figure 6A). Of those, 107 were differentially expressed in OPCs from cuprizone-treated compared to control mice (Figure 6B and Table S19). The most differentially expressed were *Bbc3*, *Gadd45b* and *Hist1h3d*, all of which were upregulated in OPCs after cuprizone treatment.

## 3. Discussion

In this in silico study, we analyzed demyelination/remyelination transcriptomic markers in mouse CC, microglia and OPCs, as well as human MS samples, and we examined the crosstalk between microglia and OPCs during demyelination. In demyelination, we identified 166 DEGs in the CC, 12 in microglia and 2730 in OPCs. Ninety-five genes were differentially expressed in both CC and in OPCs, and one gene was differentially expressed in both microglia and OPCs. In remyelinating compared to demyelinating CC, there were 611 DEGs. These demyelination/remyelination markers were associated with molecular functions and pathways related to myelin, central nervous system cell populations and lipid metabolism. Regarding the microglia–OPC crosstalk during demyelination, we identified 47 ligands in microglia, 43 OPC receptors and 115 target genes in OPCs (Figure 3). In human MS samples, we identified 252 DEGs, and from those, 13 were shared with demyelinated mouse CC (11 of them were also common with OPCs). This limited overlapping between species may be explained by the restricted transcriptomic MS sample availability, the area heterogeneity, the different nature of the lesions (chronic versus acute demyelination), and the limitations of the cuprizone model. However, the overlapping of some relevant genes detected in human MS samples involved in myelination, such as *Mobp* or *Mbp,* highlights the validity of this transcriptomic analysis, and urges the undertaking of further studies to investigate the role of other common DEGs between human MS and CC, such as Jam3 or CD22. Common genes among the 10 most significant DEGs in early and late demyelinating CC, which included *Gdf15, Ninj2* and *Trib3*, have been previously associated with myelination. *Gdf15* is a member of the transforming superfamily of proteins, such as TGF-β [23], and was downregulated in demyelinating CC in our study. *Gdf15* is found in oligodendrocytes of the CC, and it is overexpressed in patients with stabilized MS [24], and also in functional recovery after traumatic spinal cord injury (SCI) [25]. It could be possible that the observed downregulation is linked to the severe decrease in oligodendrocytes in the CC. *Ninj2* was found upregulated during early and late demyelination and when comparing late demyelination–remyelination. *Ninj2* is associated with neurite outgrowth and myelination networks [26,27]. Therefore, the observed *Ninj2* upregulation during demyelination may indicate a feedback loop to restore myelin in the CC. *Trib3* was downregulated in early and late demyelination and during remyelination when compared to late demyelination. *Trib3* has been associated with myelin destruction [28,29].

The 12 DEGs identified in microglia included *Lpl* [30] and *Olr1* [31], which have been previously associated with myelination. In addition, the common DEG in demyelinating microglia and OPCs, *Lgals3*, has been consistently identified in microglia and oligodendrocytes in the cuprizone demyelination–remyelination model [32,33], and its role in oligodendrocyte maturation has been dissected [34].

Three of the 10 most significant DEGs in demyelinating OPCs have been associated with demyelination, and were also found among the most significant DEGs in the CC: *Atf5, Trib3* and *Cdkn1a*. *Atf5* was downregulated in the CC but upregulated in OPCs. *Atf5* downregulation is necessary for the differentiation of neurons, astrocytes and OPCs, while *Atf5* is not expressed in mature oligodendrocytes [35]. One explanation for being downregulated in the CC and upregulated OPCs could be that during demyelination, OPCs are not capable of differentiating due to the overexpression of *Atf5*. However, other cell types may be trying to counterbalance this effect by downregulation of *Atf5. Trib3* was also downregulated in the CC under both demyelination and remyelination conditions, but upregulated in OPCs. *Trib3* is expressed in the endoplasmic reticulum of oligodendrocytes, and its overexpression has been linked to stress responses, such as myelin breakdown or cuprizone [29,30]. Our results for OPCs agree with the available literature. The observed downregulation in the CC could be related to the different cell types present in the CC, in addition to oligodendrocytes and OPCs. On the other hand, *Cdkn1a* (p21) is a ubiquitously expressed cell-cycle-dependent kinase inhibitor that has been found expressed in most oligodendrocytes of demyelinated lesions [36]. Other cell-cycle-dependent kinases of the same family, such as *Cdkn1c*, were identified as DEGs in human MS samples.

GO term analysis showed that DEGs were associated with different biological and molecular processes related to the CNS, such as to constituents of myelin, axon extension and neurogenesis, while associated pathways included cholesterol and sphingolipid metabolism. Cholesterol and sphingolipids are major components of myelin, and their altered metabolism is linked to different neurological and neurodegenerative disorders [37,38]. The association of the most significant DEGs with demyelination/remyelination, as well as the whole DEGs with myelin-related functions and pathways, argues for the robustness of the method for DEG analysis.

Among the 47 potential microglia ligands, *Spp1* and *Plau* expression were higher in cuprizone-treated compared to control mice. *Spp1* upregulation has been observed in response to injury in different animal models of CNS trauma [39,40], in experimental autoimmune encephalomyelitis (EAE) [41], and in microglia/macrophages and astrocytes during cuprizone-induced demyelination [42]. *Spp1* has also been related to enhanced myelin formation and prevents apoptosis of human OPCs [43,44]. Our results are in line with previous results published based on the cuprizone model [42], showing that that *Spp1* is upregulated during cuprizone-induced demyelination. In addition, *Plau* has been associated with the promotion of axon outgrowth in myelin presence after injury [35].

Regarding the identified OPC target genes, the most differentially expressed were *Bbc3, Gadd45b* and *Hist1h3d*. *Bbc3* is a pro-apoptotic gene that promotes premyelinating oligodendrocyte cell death [45]. Our results indicate that *Bbc3* is upregulated in cuprizone-treated compared to control mice. These results indicate that *Bbc3* overexpression in cuprizone-treated mice could be linked to fostering programmed cell death in OPCs. Moreover, *Gadd45b*, which was upregulated in cuprizone-treated OPCs, downregulates JNK signaling [46], a signaling event that is necessary for OPC proliferation [47]. *Hist1h3d* is a gene belonging to the histone cluster family, and is involved in transcriptional control; its expression has been described in other cell types, such as microglia [48].

The performed analysis also revealed that among the 43 identified receptors in OPCs, 15 were differentially expressed. Some of them were upregulated (PLXNB2, TNFRSF1A, ACVR1, LRP1, ITGB5 and AXL) or downregulated (JAM3, HHIP, LDLR, FGFR2, ITGB4, EPHB1, LPAR1, TYRO3 and SORL1) after cuprizone treatment. Most of these OPC receptors have been associated with CNS myelin and oligodendrocytes, and related pathologies. Regarding upregulated receptors, mutated *Tnfrsf1a* is a risk factor for MS due to it interfering in the TNF-α signaling pathway, leading to an increase in proinflammatory signals [49]. In addition, TNF has been associated with an accelerated onset of EAE [50]. Therefore, the increase in *Tnfrsf1a* after cuprizone treatment could modulate the TNF signaling pathway and contribute to the observed demyelinating phenotype in the cuprizone model. Mutations in the *Acvr1* gene arrest oligodendrocyte differentiation [51], and *Acvr1* can regulate OPC differentiation and myelin production [52]. Our results reinforce the role of *Acvr1* during demyelination by inhibiting oligodendrocyte differentiation. *Lrp1* is a negative regulator of OPC differentiation, and its deletion is associated with OPC proliferation and the generation of new mature oligodendrocytes [53]. Therefore, *Lrp1* increase in the cuprizone model would reduce the number of newborn mature oligodendrocytes, and OPCs would not differentiate into oligodendrocytes. *Itgb5* is a member of the integrin family, which is necessary for oligodendrocyte proliferation, survival and maturation [54]. Upregulation of *Itgb5* after cuprizone treatment could promote OPC functioning. Finally, *Axl* is necessary for the maintenance of axonal integrity and remyelination, since its loss induces higher oligodendrocyte apoptosis, reduced remyelination and extensive axonal damage after cuprizone treatment [16,17]. Therefore, *Axl* increase after cuprizone treatment could be an attempt to protect cells from injury.

Regarding the identified downregulated OPC receptors, Jam3 has been reported as a Jam2 counter receptor [55,56]. Jam2 inhibits oligodendrocyte wrapping but does not affect OPC differentiation, proliferation or migration. Therefore, the downregulation of Jam3 could contribute to the loss of myelin integrity that occurs in cuprizone-induced demyelination. Interestingly, Jam3 was also identified as a common DEG between human MS samples and CC analysis. Hhip interacts with Sonic Hedgehog (shh), and it has been reported that Shh signaling protects against demyelination and favors remyelination by OPC proliferation [57]. Ldlr has been related to correct myelination, both directly [58] and through cholesterol metabolism. In addition, low levels of Ldlr have been associated with failure in OPC differentiation [59], consistent to the observed behavior at 4 weeks of cuprizone treatment. Fgfr2 is necessary for upregulating myelin gene expression, regulating myelin thickness and OPC differentiation, and fostering myelin growth during developmental myelination [50]. Additionally, Fgfr2 promotes remyelination in chronic demyelinated lesions but not in acute lesions [60]. Itgb4 has been related to OPC proliferation [61], and its deletion leads to delayed peripheral nerve regeneration in the peripheral nervous system (PNS) [62]. Ephb1 has been described as implicated in T cell differentiation and migration to inflammatory sites in both EAE and MS [63], and stimulates myelin sheath formation [64]. Lpar1 expression has been reported as necessary for oligodendrocyte differentiation and myelination, and its deletion has been correlated with myelin alterations and oligodendrocyte death [65]. The observed downregulation of Lpar1 in cuprizone-treated mice is in line with published data, contributing to oligodendrocyte death during demyelination. Finally, Tyro3 expression occurs at the onset of remyelination and enhances myelination [66].

Our results from the microglia–OPC crosstalk analysis show that most of the identified microglia ligands, OPC receptors and target genes play important roles in myelination. These results point also to ligands, receptors and target genes that have not been previously associated with myelination, and that could also be involved in this process. In addition, our results highlight the potential of the NicheNet model to study cellular crosstalk not only in co-cultured cell types, but also with transcriptomic data of independent studies where cell types have received the same treatment. This observation may open the possibility of analyzing the crosstalk between cell types that cannot be co-cultured or for which transcriptomic data are available.

We combined all the available studies on the CC, microglia and OPCs in mice treated with cuprizone analyzed with microarrays, and used the same pipeline to obtain DEGs; we validated our results by comparing them with human MS samples. DEGs were associated with molecular functions and pathways related to myelination, and some of them were identified in patients with MS. We conducted a crosstalk analysis based on two microglia studies and one OPC study with independent transcriptomic data, and the results from the crosstalk analysis are in accordance with the demyelination/remyelination processes. There are, however, some limitations that should be considered. Some experimental conditions of the studies used were different, such as slight time point variation for cuprizone demyelination (either 4 or 5 weeks), or the use of only males in the OPC transcriptome but both sexes in the rest of the studies [67,68,69,70]. These changes may be factors that could introduce variability in DEGs detection, despite other factors affecting cuprizone treatment, such as the animal strain or age, being the same for all cases [71]. Additionally, because the number of samples in the analyzed studies was small, the number of DEGs in cell types and tissues after cuprizone treatment could be affected, which could impact in the crosstalk analysis. In addition, we did not know the cell type composition of the corpus callosum samples. However, we performed an in silico composition analysis that yielded the presence of six cell types, with neurons, astrocytes and oligodendrocytes being the most abundant (Tables S12 and S13). Since most of the DEGs, ligands, targets and receptors identified in the analysis were associated with myelination, and some of them were shared with human MS samples, we consider our results to be robust.

## 4. Materials and Methods

### 4.1. Data

Two searches were performed in GEO/ArrayExpress. One to identify studies that analyzed gene expression by microarray in mice treated, or not treated, with cuprizone, and another one to identify studies that analyzed gene expression by microarray in MS patient white matter samples (Table S1).

Raw data for the six studies identified were obtained from GEO. There were two studies on mice microglia, one on the mouse CC, one on mice OPCs, and two on human white matter [67,68,69,70,72].

### 4.2. Identification of Demyelination/Remyelination Biomarkers

**Figure 7:**
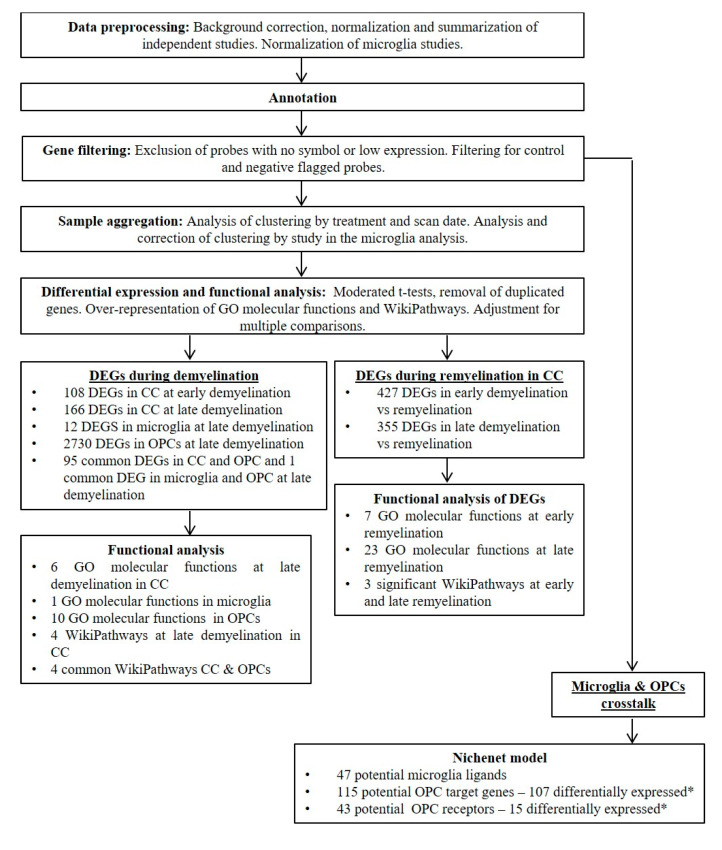
Methodology workflow and summary of results. * In OPCs from cuprizone-treated mice compared to OPCs from control mice. CC, corpus callosum; DEGs, differentially expressed genes; GO, gene ontology; MS, multiple sclerosis; OPC, oligodendrocyte progenitor cell.

The pipeline to identify demyelination/remyelination DEGs in samples from mice, and to identify DEGs in human white matter samples from MS patients and controls, was as follows. We performed data preprocessing, annotation, gene filtering and DEG analysis in all the analyses. In addition, we performed a functional analysis for the DEGs identified in the studies with mouse samples (Figure 7). Demyelination DEGs were examined in the CC, microglia and OPC, regarding the studies, as well as in the MS samples. Remyelination DEGs were only examined in the CC.

Quality control of raw data was performed with boxplots and density plots of log intensities and with MA plots. In Affymetrix studies, we also inspected images and plots of relative log expression values and of normalized unscaled standard errors. Preprocessing was performed as follows. Background correction was performed with convolution of normal and exponential distributions, then, quantile normalization was used. Finally, summarization was performed with the median-polish method for Affymetrix studies, and by replacing array replicates with their average for Agilent studies. For the microglia analysis and for the white matter analysis, normalized data of the two available studies were combined using quantile normalization. Normalized data were examined with boxplots.

After preprocessing, the data were annotated and filtered. We excluded probes with no symbol or low expression, considering, in that case, the 30th percentile of lower intensity in several arrays as the smallest experimental group. Gene filtering was included in Agilent studies for control and negative flagged probes. Sample clustering by treatment and scan date was analyzed with hierarchical clustering and principal component analysis. There was no scan date clustering. Treatment clustering was clear in microglia and OPC data, and moderate in CC and white matter data. We excluded the most different half of the samples in the CC analyses based on sample clustering. For the microglia and the white matter analyses, clustering by study was identified and corrected with the ComBat method.

Differential expression was analyzed with moderated *t*-tests including an intensity trend for prior variance and robustifying for outlier sample variances. *p*-values were adjusted for multiple comparisons using the Benjamini–Hochberg (BH) method. Duplicated assigned genes were removed by selecting the one with the lowest *p*-value.

In the analyses of mice samples, DEGs were visualized with volcano plots. Sample clustering by gene expression of all DEGs and by gene expression of the most significant DEGs was visualized with heatmaps (Figure S1). Functional analysis of DEGs was performed on the three cell types/regions separately, and on the DEGs identified in more than one cell type/region. Considering the large number of DEGs in OPCs, functional analysis was undertaken with an adjusted *p*-value < 0.001. Then, we performed over-representation functional analyses with gene ontology (GO) molecular-level functions and WikiPathways. *p*-values were obtained with the hypergeometric test and adjusted with the BH method. Significant GO molecular functions and pathways were visualized with dot plots.

### 4.3. Cell Type Analysis in CC Samples

CIBERSORTx [73] was used to perform the cell type composition analysis. We used, as the signature matrix, the gene expression signature of the mouse brain obtained from single-cell RNA sequencing by Donovan et al. [74] Mixture data were the normalized and filtered expression data of the five CC samples included in the 4-week demyelination analysis. Quantile normalization and 100 permutations were used to obtain the results. Pearson’s correlation coefficient and RMSE of signature genes between original mixture data and the estimated mixture were computed. Sensitivity analyses were performed using the raw and normalized data, by enabling batch correction, by not including quantile normalization and by increasing the number of permutations.

### 4.4. Microglia and OPC Crosstalk

We applied the NicheNet model [75] to examine the intercellular communication between microglia (sender cells) and OPCs (receiver cells). OPC and microglia genes used for the analysis had been previously normalized, annotated and filtered. Gene filtering for low expression included genes below the 60th (microglia) and the 75th (OPCs) percentile of intensity. The gene set of interest was defined as the OPC DEGs with a *p*-value < 0.01. OPC background genes were all those genes expressed by OPCs except for genes contained in the set of interest. Potential microglia ligands were selected from those expressed by microglia that were able to bind putative OPC receptors. Potential OPC receptors were those expressed by these cells that could bind a putative microglia ligand. Putative ligand–receptor associations were restricted to the ones described in curated databases.

Ligand activity was determined by how well the ligands predicted changes in the expression in the gene set of interest when compared to background genes, and was categorized using the Pearson correlation coefficient. OPC target genes were analyzed according to those expressed by OPCs that belonged to the 100 most strongly predicted target genes of the top-ranked microglia ligands. Both ligands and receptors were organized by hierarchical clustering. Microglia ligand and OPC receptor/target gene association was visualized with heatmaps. Expression of the identified microglia ligand and OPC receptor/target gene association was compared between cuprizone samples and controls using *t*-tests, with adjusted *p*-values for multiple comparisons with the BH method.

Analyses were conducted in R-4.0.4.

## 5. Conclusions

In this study, we established a strategy to select genes associated with different cell types or tissues in de-/remyelination processes in cuprizone-treated mice. Genes identified in this study were related to molecular functions and pathways associated with myelination. These genes are potential candidates for determining demyelination and remyelination in cuprizone-treated mice, and the whole strategy could be used to analyze different cell types in other demyelination and remyelination models and/or treatments. We also performed an in silico analysis to identify ligands, receptors and target genes involved in the microglia–OPC crosstalk during demyelination. Some of them were associated with demyelination, as they were differentially expressed in samples from cuprizone-treated compared to control mice. This result suggests that crosstalk analysis can be studied using data from different experiments, at least in cuprizone-induced demyelination experimental models. We discovered novel potential demyelination biomarkers as well as novel ligands, receptors and target genes involved in microglia–OPC crosstalk, which demand additional validation studies.

## Data Availability

Datasets used in this study (GSE100663, GSE84113, GSE66926, GSE48872, GSE38010, GSE52139) can be found in the GEO dataset browser of the NIH (https://www.ncbi.nlm.nih.gov/gds/, accessed on 31 October 2022).

## Supplementary Materials

The following supporting information can be downloaded at: https://www.mdpi.com/article/10.3390/ijms232314868/s1.
