# Supplementary material for: Deciphering the Genetic Crosstalk between Microglia and Oligodendrocyte Precursor Cells during Demyelination and Remyelination Using Transcriptomic Data
Source: Int J Mol Sci. 2022 Nov 28;23(23):14868. doi: 10.3390/ijms232314868 (PMC9738937; doi:10.3390/ijms232314868)
Supplement: Supplementary file 1 [file ijms-23-14868-s001.zip › ijms-1957495-supplementary.pdf]

Figure S1

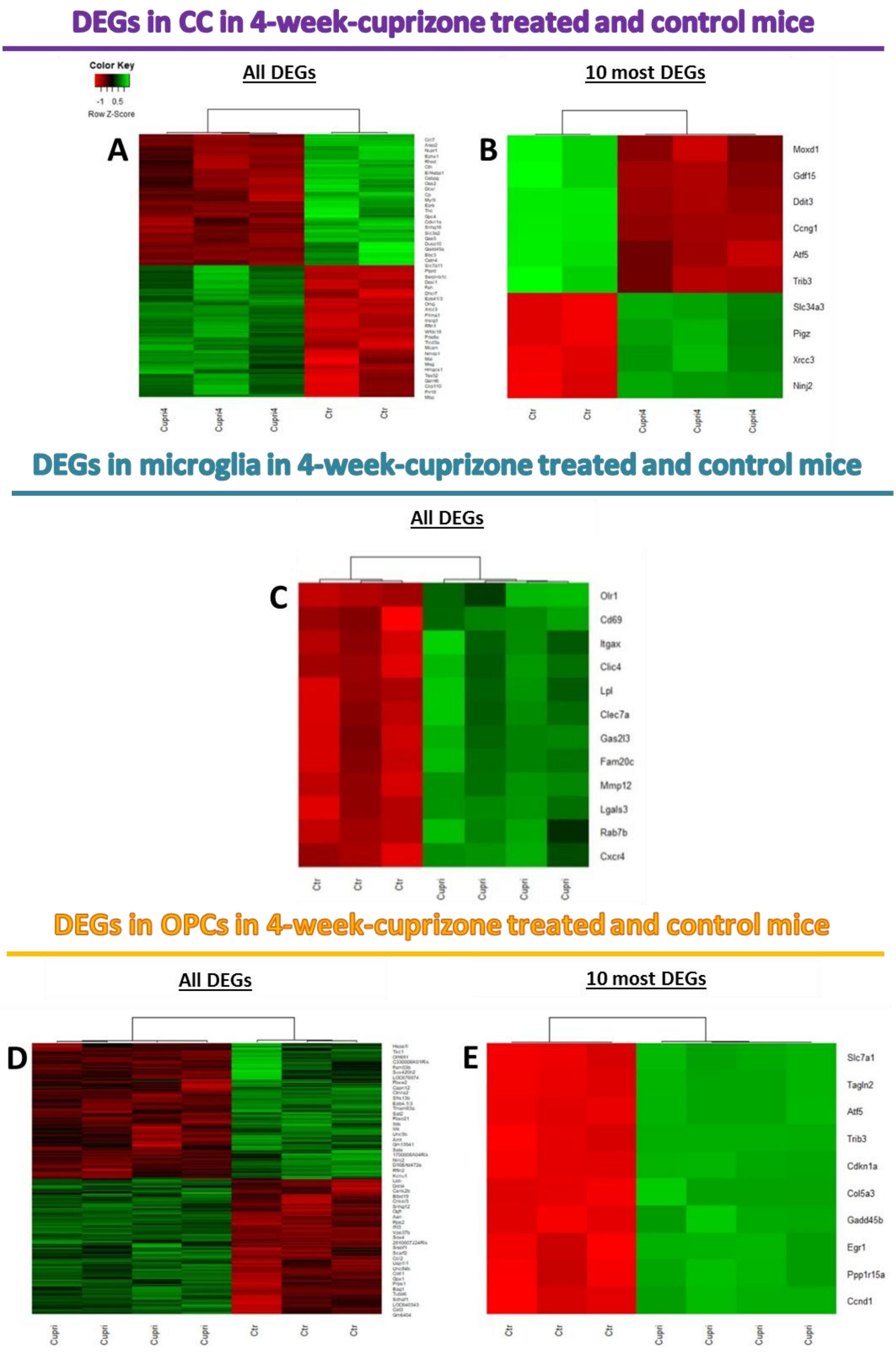
DEGs in CC in 4-week-cuprizone treated and control mice
Color Key
-1 0.5
Row Z-Score
All DEGs
10 most DEGs
A
Cupri4
Cupri4
Cupri4
Ctr
Ctr
B
Moxd1
Gdf15
Ddit3
Ccng1
Atf5
Trib3
Slc34a3
Pigz
Xrcc3
Ninj2
Ctr
Ctr
Cupri4
Cupri4
Cupri4
DEGs in microglia in 4-week-cuprizone treated and control mice
All DEGs
C
Olr1
Cd69
Itgax
Clic4
Lpl
Clec7a
Gas2l3
Fam20c
Mmp12
Lgals3
Rab7b
Cxcr4
Ctr
Ctr
Ctr
Cupri
Cupri
Cupri
Cupri
DEGs in OPCs in 4-week-cuprizone treated and control mice
All DEGs
10 most DEGs
D
Cupri
Cupri
Cupri
Cupri
Ctr
Ctr
Ctr
E
Slc7a1
Tagln2
Atf5
Trib3
Cdkn1a
Col5a3
Gadd45b
Egr1
Ppp1r15a
Ccnd1
Ctr
Ctr
Ctr
Cupri
Cupri
Cupri
Cupri

Figure S1. Sample clustering by expression of differentially expressed genes (DEGs) in *corpus callosum* (CC), microglia and oligodendrocyte progenitor cells (OPCs) samples of control mice and 4-week cuprizone-treated mice. Heatmap and hierarchical clustering were performed with the normalized and filtered expression data, using all DEGs and the 10 most DEGs. Color key indicates the relative expression level of genesacross all samples: red color represents an expression level below mean, green color represents expressionhigher than the mean. (A-B) Analysis of CC samples and DEGs. (C) Analysis of microglia samples and DEGs. (D-E) Analysis of OPCs samples and DEGs.

Figure S2

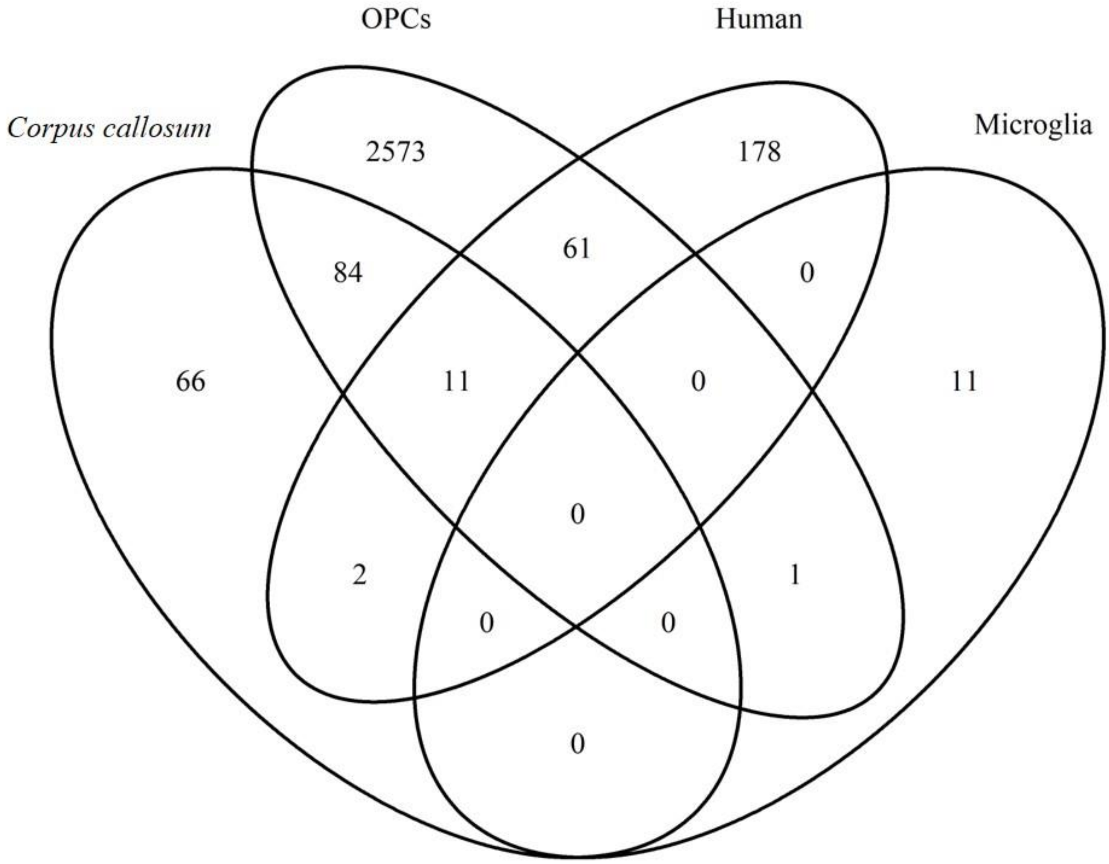

Figure S2. Common differentially expressed genes (DEGs) identified in white matter from multiple sclerosis (MS) samples and in corpus callosum (CC), microglia and oligodendrocyte progenitor cell (OPCs) samples from mice. Venn's diagram showing the number of common DEGs. Samples from CC and microglia were obtained after a 4-week treatment with cuprizone, while OPC samples were collected after a 5-week cuprizone treatment.

Supplementary Tables

Table S1. GEO studies using brain samples/cells from control and cuprizone-treated mice, and GEO studies using white matter samples from multiple sclerosis and control patients, where gene expression was analyzed with microarrays.

GEO Study	Platform	Cell type/brain region	Length of cuprizone treatment	Remyelination	Number of samples in control group	Number of samples in treatment/ MS group
GSE100663	Agilent wholemouse 4x44k	Mice corpus callosum	2/4 weeks	4 weeks of cuprizone treatment + 14 days norma l diet	3	3 (2 weeks)/4 (4 weeks)
GSE84113	Affymetri x Mouse Gene 1.0 ST	Mice microglia	4 weeks	---	2	3
GSE66926	Affymetrix Mouse Gene 1.0 ST	Mice microglia	4 weeks	---	1	1
GSE48872	Agilent whole mouse 4x44k	Mice OPCs	5 weeks	---	3	4
GSE38010	Affymetrix Human Genome U133 Plus 2.0 Array	Brain lesions from MS patients and white matter from healthy controls	---	---	2	5
GSE52139	Affymetrix Human Genome U133 Plus 2.0 Array	Spinal cord periplaque samples and normal appearing white matter (control) from MS patients	---	---	8	8

Table S2. Differentially expressed genes in *corpus callosum* of mice treated with cuprizone for 2 weeks compared to control.

Symbol	Gene Name	Log2 fold change	p-value	Adjusted p-value
Cdkn1a	cyclin-dependent kinase inhibitor1A (P21)	-4.32	1.89E-07	4.55E-03
Trib3	tribbles pseudokinase 3	-4.64	1.11E-06	1.34E-02
Gdf15	growth differentiation factor 15	-4.3	2.79E-06	1.63E-02
Pigz	phosphatidylinositol glycan anchorbiosynthesis, class Z	3.1	2.98E-06	1.63E-02
Tgm1	transglutaminase 1, K polypeptide	-3.13	3.43E-06	1.63E-02
B230206H07Rik	RIKEN cDNA B230206H07 gene	3.46	4.86E-06	1.63E-02
Ninj2	ninjurin 2	3.22	5.13E-06	1.63E-02
Eif4ebp1	eukaryotic translation initiationfactor 4E binding protein 1	-2.31	5.88E-06	1.63E-02
Slc34a3	solute carrier family 34 (sodiumphosphate), member 3	3.19	6.11E-06	1.63E-02
Atf5	activating transcription factor 5	-2.92	8.27E-06	1.89E-02
Sesn2	sestrin 2	-2.07	9.95E-06	1.89E-02
Cacna2d4	calcium channel, voltage-dependent, alpha 2/delta subunit 4	3.16	1.06E-05	1.89E-02
Tmprss5	transmembrane protease, serine 5(spinesin)	2.79	1.08E-05	1.89E-02
Xrcc3	X-ray repair complementingdefective repair in Chinese hamster cells 3	2.77	1.13E-05	1.89E-02
Gjc2	gap junction protein, gamma 2	3.2	1.25E-05	1.89E-02
Serpinb1a	serine (or cysteine) peptidaseinhibitor, clade B, member 1a	2.42	1.26E-05	1.89E-02
Carns1	carnosine synthase 1	2.58	1.52E-05	2.00E-02
Ppp1r14a	protein phosphatase 1, regulatoryinhibitor subunit 14A	4.29	1.60E-05	2.00E-02
Ldlr	low density lipoprotein receptor	2.33	1.81E-05	2.00E-02
Ccng1	cyclin G1	-1.9	1.81E-05	2.00E-02
Mucl1	mucin-like 1	2.41	1.90E-05	2.00E-02
Tmem125	transmembrane protein 125	4.25	1.88E-05	2.00E-02
Wfdc18	WAP four-disulfide core domain18	1.93	1.95E-05	2.00E-02
Atf3	activating transcription factor 3	-2.17	2.23E-05	2.00E-02
Nupr1	nuclear protein transcriptionregulator 1	-2.8	2.26E-05	2.00E-02
Maff	v-maf musculoaponeurotic fibrosarcoma oncogene family, protein F (avian)	-2.75	2.29E-05	2.00E-02
Moxd1	monooxygenase, DBH-like 1	-2.51	2.33E-05	2.00E-02
Cebpd	CCAAT/enhancer binding protein(C/EBP), delta	-1.96	2.37E-05	2.00E-02
Wfdc18	WAP four-disulfide core domain18	2	2.42E-05	2.00E-02
Tubb6	tubulin, beta 6 class V	-2.07	2.75E-05	2.20E-02
Klf4	Kruppel-like factor 4 (gut)	-1.81	3.07E-05	2.34E-02
Smtnl2	smoothelin-like 2	3.19	3.26E-05	2.34E-02
Adssl1	adenylosuccinate synthetase like 1	2.23	3.31E-05	2.34E-02
Fkbp5	FK506 binding protein 5	-1.94	3.31E-05	2.34E-02
Sgk2	serum/glucocorticoid regulated kinase 2	3.93	3.46E-05	2.38E-02
Rtkn2	rhotekin 2	2.23	3.79E-05	2.53E-02
Rab37	RAB37, member RAS oncogene family	2.18	4.38E-05	2.66E-02
Enpp2	ectonucleotide pyrophosphatase/phosphodiesterase 2	1.94	4.57E-05	2.66E-02
Ccp110	centriolar coiled coil protein 110	2.17	4.58E-05	2.66E-02
Klk6	kallikrein related-peptidase 6	4.47	4.59E-05	2.66E-02
Hmgcs1	3-hydroxy-3-methylglutaryl-Coenzyme A synthase 1	1.81	4.64E-05	2.66E-02
Serpinb1c	serine (or cysteine) peptidase inhibitor, clade B, member 1c	2.17	4.88E-05	2.73E-02
Slc7a5	solute carrier family 7 (cationic amino acid transporter, y+ system),member 5	-1.88	5.11E-05	2.79E-02
Gamt	guanidinoacetate methyltransferase	2.79	5.35E-05	2.81E-02
Nmral1	NmrA-like family domain containing 1	2.22	5.38E-05	2.81E-02
Eda2r	ectodysplasin A2 receptor	-2.45	5.73E-05	2.90E-02
Lctl	lactase-like	2.93	5.88E-05	2.90E-02
Slc17a6	solute carrier family 17 (sodium-	1.7	5.90E-05	2.90E-02

	dependent inorganic phosphatecotransporter), member 6			
Arrdc4	arrestin domain containing 4	-1.93	6.31E-05	2.97E-02
Ddit3	DNA-damage inducible transcript 3	-1.62	6.36E-05	2.97E-02
Gal3st1	galactose-3-O-sulfotransferase 1	2.49	6.81E-05	3.05E-02
Osmr	oncostatin M receptor	-1.52	6.84E-05	3.05E-02
Fndc11	fibronectin type III domain containing 11	1.72	7.33E-05	3.15E-02
Bcl6	B cell leukemia/lymphoma 6	-1.51	7.93E-05	3.25E-02
Plekhg1	pleckstrin homology domaincontaining, family G (with RhoGef domain) member 1	1.6	7.94E-05	3.25E-02
Map3k6	mitogen-activated protein kinase kinase kinase 6	-1.77	7.96E-05	3.25E-02
Gtse1	G two S phase expressed protein 1	-1.71	8.34E-05	3.27E-02
Cebpb	CCAAT/enhancer binding protein (C/EBP), beta	-1.51	8.38E-05	3.27E-02
Nes	nestin	-1.69	1.02E-04	3.80E-02
Hmox1	heme oxygenase 1	-1.77	1.02E-04	3.80E-02
Mast4	microtubule associatedserine/threonine kinase family member 4	1.55	1.03E-04	3.80E-02
Runx1	runt related transcription factor 1	-1.62	1.05E-04	3.80E-02
Nectin4	nectin cell adhesion molecule 4	2.95	1.07E-04	3.80E-02
Slc7a11	solute carrier family 7 (cationic amino acid transporter, y+ system),member 11	-2.01	1.09E-04	3.80E-02
Fgl1	fibrinogen-like protein 1	-1.46	1.11E-04	3.80E-02
Pdlim2	PDZ and LIM domain 2	3.15	1.14E-04	3.80E-02
Dcaf12l2	DDB1 and CUL4 associated factor 12-like 2	-1.57	1.15E-04	3.80E-02
Gas5	growth arrest specific 5	-1.43	1.16E-04	3.80E-02
Ifrd1	interferon-related developmental regulator 1	-1.41	1.19E-04	3.80E-02
Gpr62	G protein-coupled receptor 62	2.99	1.22E-04	3.80E-02
Cntn2	contactin 2	2.41	1.22E-04	3.80E-02
Pappa	pregnancy-associated plasma protein A	-2.27	1.23E-04	3.80E-02
Serpinb1b	serine (or cysteine) peptidase inhibitor, clade B, member 1b	1.84	1.23E-04	3.80E-02
Snhg16	small nucleolar RNA host gene 16	-1.36	1.28E-04	3.91E-02
Pcyt2	phosphate cytidylyltransferase 2, ethanolamine	1.54	1.40E-04	4.21E-02
Rps27l	ribosomal protein S27-like	-1.42	1.45E-04	4.32E-02
Emilin3	elastin microfibril interfacer 3	1.67	1.51E-04	4.42E-02
Bbc3	BCL2 binding component 3	-1.69	1.54E-04	4.46E-02
Tmem141	transmembrane protein 141	1.76	1.58E-04	4.51E-02
Slc3a2	solute carrier family 3 (activatorsof dibasic and neutral amino acid transport), member 2	-1.4	1.60E-04	4.51E-02
Prima1	proline rich membrane anchor 1	2.24	1.65E-04	4.51E-02
Epb41l3	erythrocyte membrane protein band 4.1 like 3	1.82	1.64E-04	4.51E-02
Prr18	proline rich 18	2.61	1.69E-04	4.58E-02
Lgals2	lectin, galactose-binding, soluble 2	1.81	1.76E-04	4.65E-02
Arid5a	AT rich interactive domain 5A (MRF1-like)	-1.97	1.78E-04	4.65E-02
Hid1	HID1 domain containing	1.55	1.81E-04	4.68E-02
Nfil3	nuclear factor, interleukin 3, regulated	-1.32	1.83E-04	4.68E-02
Scgb3a1	secretoglobin, family 3A, member 1	-1.57	1.87E-04	4.68E-02
Pde8a	phosphodiesterase 8A	2.26	1.92E-04	4.68E-02
Tnc	tenascin C	-2.06	1.93E-04	4.68E-02
Il12rb1	interleukin 12 receptor, beta 1	2.63	1.95E-04	4.68E-02
Lgi3	leucine-rich repeat LGI family, member 3	2.18	1.96E-04	4.68E-02
Mobp	myelin-associated oligodendrocytic	2.5	1.98E-04	4.68E-02

	basic protein			
Sspo	SCO-spondin	2.69	1.98E-04	4.68E-02
5033421B08Rik	RIKEN cDNA 5033421B08 gene	2.04	2.02E-04	4.73E-02
Srd5a1	steroid 5 alpha-reductase 1	1.54	2.09E-04	4.84E-02
Galnt6	polypeptide N-acetylgalactosaminyltransferase 6	2.22	2.18E-04	4.87E-02
Prkcq	protein kinase C, theta	1.62	2.20E-04	4.87E-02
Aoc1	amine oxidase, copper-containing 1	1.96	2.22E-04	4.87E-02
1110038B12Rik	RIKEN cDNA 1110038B12 gene	-1.33	2.25E-04	4.87E-02
Cables1	CDK5 and Abl enzyme substrate 1	-1.6	2.27E-04	4.87E-02
Fdps	farnesyl diphosphate synthetase	1.34	2.29E-04	4.87E-02
Mt2	metallothionein 2	-1.43	2.31E-04	4.87E-02
Sh3gl3	SH3-domain GRB2-like 3	1.65	2.31E-04	4.87E-02
Depdc1b	DEP domain containing 1B	2.25	2.38E-04	4.87E-02
Fah	fumarylacetoacetate hydrolase	1.72	2.35E-04	4.87E-02
Pllp	plasma membrane proteolipid	2.27	2.39E-04	4.87E-02
Gm4221	predicted gene 4221	1.92	2.39E-04	4.87E-02

Table S3. Differentially expressed genes in *corpus callosum* of mice treated with cuprizone for 4 weeks compared to control.

Symbol	Gene name	Log2 fold change	p-value	Adjusted p-value
Gdf15	growth differentiation factor 15	-3.86	3.37e-07	8.51e-03
Pigz	phosphatidylinositol glycan anchorbiosynthesis, class Z	2.54	1.35e-06	1.10e-02
Trib3	tribbles pseudokinase 3	-4.40	1.77e-06	1.10e-02
Ninj2	ninjurin 2	1.92	2.01e-06	1.10e-02
Ccng1	cyclin G1	-1.93	2.41e-06	1.10e-02
Slc34a3	solute carrier family 34 (sodiumphosphate), member 3	1.76	2.78e-06	1.10e-02
Atf5	activating transcription factor 5	-3.41	3.05e-06	1.10e-02
Xrcc3	X-ray repair complementing defectiverepair in Chinese hamster cells 3	1.91	4.10e-06	1.29e-02
Ddit3	DNA-damage inducible transcript 3	-1.70	5.40e-06	1.51e-02
Moxd1	monooxygenase, DBH-like 1	-2.59	7.51e-06	1.60e-02
Eda2r	ectodysplasin A2 receptor	-1.12	7.61e-06	1.60e-02
Smtnl2	smoothelin-like 2	1.65	8.68e-06	1.69e-02
Ppp1r14a	protein phosphatase 1, regulatoryinhibitor subunit 14A	3.67	9.42e-06	1.70e-02
Sesn2	sestrin 2	-2.20	1.05e-05	1.74e-02
Cdkn1a	cyclin-dependent kinase inhibitor 1A(P21)	-3.72	1.13e-05	1.74e-02
Tmem125	transmembrane protein 125	3.46	1.21e-05	1.74e-02
Nat8	N-acetyltransferase 8 (GCN5-related)	-1.57	1.24e-05	1.74e-02
Tmprss5	transmembrane protease, serine 5(spinesin)	1.65	1.34e-05	1.74e-02
Slc7a5	solute carrier family 7 (cationic amino acid transporter, y+ system), member 5	-1.73	1.38e-05	1.74e-02
Wfdc18	WAP four-disulfide core domain 18	1.87	1.47e-05	1.77e-02
Slc7a11	solute carrier family 7 (cationic amino acid transporter, y+ system), member 11	-2.37	1.75e-05	1.87e-02
Fibin	fin bud initiation factor homolog (zebrafish)	-1.70	1.82e-05	1.87e-02
Klf4	Kruppel-like factor 4 (gut)	-1.71	1.84e-05	1.87e-02
Eif4ebp1	eukaryotic translation initiation factor 4Ebinding protein 1	-2.33	1.85e-05	1.87e-02
Slc3a2	solute carrier family 3 (activators of dibasic and neutral amino acid transport), member 2	-1.54	1.94e-05	1.89e-02
Arap2	ArfGAP with RhoGAP domain, ankyrin repeat and PH domain 2	-1.39	2.08e-05	1.95e-02
Gzmm	granzyme M (lymphocyte met-ase 1)	-1.60	2.20e-05	1.98e-02
Sgk2	serum/glucocorticoid regulated kinase 2	2.93	2.78e-05	2.35e-02
Bbc3	BCL2 binding component 3	-1.77	3.04e-05	2.35e-02
Pappa	pregnancy-associated plasma protein A	-2.02	3.26e-05	2.35e-02

Nes	nestin	-1.85	3.28e-05	2.35e-02
Gas5	growth arrest specific 5	-1.46	3.29e-05	2.35e-02
Fdps	farnesyl diphosphate synthetase	1.28	3.37e-05	2.35e-02
Prima1	proline rich membrane anchor 1	1.25	3.40e-05	2.35e-02
Serpinb1a	serine (or cysteine) peptidase inhibitor, clade B, member 1a	1.98	3.59e-05	2.35e-02
Rhod	ras homolog family member D	-1.28	3.63e-05	2.35e-02
Ccl7	chemokine (C-C motif) ligand 7	-0.87	3.80e-05	2.39e-02
Cp	ceruloplasmin	-1.89	3.88e-05	2.39e-02
Mvd	mevalonate (diphospho) decarboxylase	1.23	4.04e-05	2.43e-02
Ldlr	low density lipoprotein receptor	1.83	4.19e-05	2.46e-02
Gjc2	gap junction protein, gamma 2	1.49	4.42e-05	2.49e-02
Ephx1	epoxide hydrolase 1, microsomal	-2.01	4.64e-05	2.49e-02
Ephx1	epoxide hydrolase 1, microsomal	-1.99	4.80e-05	2.49e-02
B230206H07Rik	RIKEN cDNA B230206H07 gene	1.50	4.81e-05	2.49e-02
Gtse1	G two S phase expressed protein 1	-1.01	4.83e-05	2.49e-02
Hmgcs1	3-hydroxy-3-methylglutaryl-Coenzyme A synthase 1	1.23	4.84e-05	2.49e-02
Snhg16	small nucleolar RNA host gene 16	-1.25	4.93e-05	2.49e-02
Galnt6	polypeptide N-acetylgalactosaminyltransferase 6	2.72	5.88e-05	2.79e-02
Gpr62	G protein-coupled receptor 62	2.50	5.98e-05	2.79e-02
Galnt6	polypeptide N-acetylgalactosaminyltransferase 6	1.22	6.01e-05	2.79e-02
Nupr1	nuclear protein transcription regulator 1	-3.63	6.02e-05	2.79e-02
Adamts1	a disintegrin-like and metallopeptidase (reprolysin type) with thrombospondintype 1 motif, 1	-1.59	6.07e-05	2.79e-02
Pcyt2	phosphate cytidylyltransferase 2, ethanolamine	1.28	6.19e-05	2.79e-02
Gadd45b	growth arrest and DNA-damage-inducible 45 beta	-1.31	6.43e-05	2.85e-02
Tnc	tenascin C	-1.71	6.56e-05	2.86e-02
Scgb3a1	secretoglobin, family 3A, member 1	-0.92	7.29e-05	3.02e-02
Nacad	NAC alpha domain containing	1.38	7.82e-05	3.19e-02
Klk6	kallikrein related-peptidase 6	2.91	8.19e-05	3.27e-02
Tlcd3a	TLC domain containing 3A	1.01	8.97e-05	3.47e-02
Tmeff1	transmembrane protein with EGF-likeand two follistatin-like domains 1	1.11	9.49e-05	3.58e-02
Fbln5	fibulin 5	-1.34	1.04e-04	3.77e-02
Dcxr	dicarbonyl L-xylulose reductase	-1.37	1.06e-04	3.77e-02
Mog	myelin oligodendrocyte glycoprotein	2.67	1.06e-04	3.77e-02
Aen	apoptosis enhancing nuclease	-1.37	1.08e-04	3.77e-02
Nmral1	NmrA-like family domain containing 1	1.92	1.08e-04	3.77e-02
Rftn1	raftlin lipid raft linker 1	1.20	1.09e-04	3.77e-02
Carns1	carnosine synthase 1	1.40	1.13e-04	3.81e-02
Ccp110	centriolar coiled coil protein 110	1.70	1.13e-04	3.81e-02
Eprs	glutamyl-prolyl-tRNA synthetase	-1.11	1.15e-04	3.82e-02
Nat8f5	N-acetyltransferase 8 (GCN5-related) family member 5	-1.40	1.19e-04	3.82e-02
Nkain1	Na+/K+ transporting ATPase interacting 1	1.65	1.20e-04	3.82e-02
Fzd1	frizzled class receptor 1	-1.74	1.25e-04	3.82e-02
Calcrl	calcitonin receptor-like	-1.31	1.27e-04	3.82e-02
Sox21	SRY (sex determining region Y)-box 21	-1.13	1.27e-04	3.82e-02
Epb41l3	erythrocyte membrane protein band 4.1 like 3	1.44	1.28e-04	3.82e-02
Pde8a	phosphodiesterase 8A	1.53	1.41e-04	4.01e-02
Dusp10	dual specificity phosphatase 10	-1.54	1.42e-04	4.01e-02
Rps27l	ribosomal protein S27-like	-1.64	1.43e-04	4.01e-02
Tgm1	transglutaminase 1, K polypeptide	-3.27	1.44e-04	4.01e-02
Rhog	ras homolog family member G	1.12	1.45e-04	4.01e-02
Arrdc4	arrestin domain containing 4	-1.54	1.46e-04	4.01e-02
Opalin	oligodendrocytic myelin paranodal andinner loop protein	3.02	1.48e-04	4.03e-02
Pla1a	phospholipase A1 member A	-0.87	1.52e-04	4.05e-02
Mapk8ip1	mitogen-activated protein kinase 8 interacting protein 1	1.49	1.52e-04	4.05e-02
Arid5a	AT rich interactive domain 5A (MRF1-like)	-1.72	1.56e-04	4.09e-02

Trp53inp1	transformation related protein 53 inducible nuclear protein 1	-1.28	1.58e-04	4.09e-02
Anln	anillin, actin binding protein	1.45	1.59e-04	4.09e-02
Sytl2	synaptotagmin-like 2	1.22	1.66e-04	4.15e-02
Ccl2	chemokine (C-C motif) ligand 2	-2.41	1.66e-04	4.15e-02
Gadd45a	growth arrest and DNA-damage-inducible 45 alpha	-1.81	1.67e-04	4.15e-02
Msmo1	methylsterol monoxygenase 1	1.08	1.68e-04	4.15e-02
Mal	myelin and lymphocyte protein, T cell differentiation protein	2.81	1.71e-04	4.15e-02
Prr18	proline rich 18	2.21	1.71e-04	4.15e-02
Synj2	synaptojanin 2	1.37	1.73e-04	4.15e-02
Ifrd1	interferon-related developmental regulator 1	-1.28	1.74e-04	4.15e-02
Adssl1	adenylosuccinate synthetase like 1	1.81	1.78e-04	4.17e-02
Kctd15	potassium channel tetramerisation domain containing 15	-1.13	1.82e-04	4.20e-02
Desi1	desumoylating isopeptidase 1	1.52	1.83e-04	4.20e-02
Ppp1r15a	protein phosphatase 1, regulatory subunit 15A	-1.37	1.86e-04	4.24e-02
Foxc1	forkhead box C1	-1.14	1.90e-04	4.25e-02
Gal3st1	galactose-3-O-sulfotransferase 1	1.77	1.91e-04	4.25e-02
Cetn4	centrin 4	-1.40	1.93e-04	4.25e-02
Maff	v-maf musculoaponeurotic fibrosarcoma oncogene family, protein F (avian)	-2.24	1.95e-04	4.25e-02
Cntn2	contactin 2	1.94	1.96e-04	4.25e-02
Tmem88b	transmembrane protein 88B	2.10	1.97e-04	4.25e-02
Cdca7	cell division cycle associated 7	1.13	2.03e-04	4.33e-02
Nipal4	NIPA-like domain containing 4	1.10	2.04e-04	4.33e-02
Cth	cystathionase (cystathionine gamma-lyase)	-1.02	2.07e-04	4.33e-02
Insig1	insulin induced gene 1	0.96	2.09e-04	4.33e-02
Cdk18	cyclin-dependent kinase 18	1.32	2.10e-04	4.33e-02
Grin2c	glutamate receptor, ionotropic, NMDA2C (epsilon 3)	1.19	2.11e-04	4.33e-02
Mgp	matrix Gla protein	-1.46	2.23e-04	4.47e-02
Ptprd	protein tyrosine phosphatase, receptor type, D	1.49	2.24e-04	4.47e-02
Lgi3	leucine-rich repeat LGI family, member 3	1.76	2.25e-04	4.47e-02
Poc1a	POC1 centriolar protein A	1.10	2.30e-04	4.47e-02
Oas2	2'-5' oligoadenylate synthetase 2	-1.20	2.32e-04	4.47e-02
Pllp	plasma membrane proteolipid	1.65	2.33e-04	4.47e-02
Tor3a	torsin family 3, member A	-1.37	2.33e-04	4.47e-02
Mcam	melanoma cell adhesion molecule	1.29	2.34e-04	4.47e-02
Fah	fumarylacetoacetate hydrolase	1.30	2.36e-04	4.47e-02
Lpin3	lipin 3	-0.81	2.41e-04	4.48e-02
Helt	helt bHLH transcription factor	0.78	2.49e-04	4.56e-02
Gpc4	glypican 4	-1.73	2.49e-04	4.56e-02
Cyb5r1	cytochrome b5 reductase 1	-1.42	2.51e-04	4.56e-02
Rgs3	regulator of G-protein signaling 3	1.04	2.56e-04	4.57e-02
Nfil3	nuclear factor, interleukin 3, regulated	-1.05	2.57e-04	4.57e-02
Mbp	myelin basic protein	2.20	2.60e-04	4.59e-02
Dlx2	distal-less homeobox 2	1.21	2.81e-04	4.84e-02
Dhcr7	7-dehydrocholesterol reductase	1.23	2.83e-04	4.84e-02
Ldlrad4	low density lipoprotein receptor class A domain containing 4	1.12	2.87e-04	4.84e-02
Ano4	anoctamin 4	0.86	2.88e-04	4.84e-02
Cyb5r2	cytochrome b5 reductase 2	-0.63	2.89e-04	4.84e-02
Paqr5	progestin and adipoQ receptor family member V	-1.17	2.89e-04	4.84e-02
Hfe	homeostatic iron regulator	-1.41	2.90e-04	4.84e-02
Sspo	SCO-spondin	1.22	2.91e-04	4.84e-02
Dcaf12l2	DDB1 and CUL4 associated factor 12-like 2	-1.27	2.94e-04	4.86e-02
Stard4	StAR-related lipid transfer (START) domain containing 4	1.23	3.02e-04	4.92e-02
Zfas1	zinc finger, NFX1-type containing 1, antisense RNA 1	-1.17	3.07e-04	4.92e-02
Rhbdd1	rhomboid domain containing 1	-1.12	3.12e-04	4.92e-02
Nxph4	neurexophilin 4	0.93	3.13e-04	4.92e-02

Plekhh1	pleckstrin homology domain containing, family H (with MyTH4 domain) member 1	1.86	3.13e-04	4.92e-02
Tpm1	tropomyosin 1, alpha	0.92	3.14e-04	4.92e-02
Phlda3	pleckstrin homology like domain, family A, member 3	-1.33	3.19e-04	4.93e-02
Fndc11	fibronectin type III domain containing 11	1.31	3.20e-04	4.93e-02
Ttll7	tubulin tyrosine ligase-like family, member 7	1.07	3.24e-04	4.96e-02
Runx1	runt related transcription factor 1	-1.06	3.28e-04	4.97e-02
Serpinb1c	serine (or cysteine) peptidase inhibitor,clade B, member 1c	0.90	3.34e-04	4.97e-02
Odc1	ornithine decarboxylase, structural 1	-0.92	3.34e-04	4.97e-02
Tex52	testis expressed 52	0.91	3.35e-04	4.97e-02
Adamtsl4	ADAMTS-like 4	0.92	3.38e-04	4.97e-02
Cerox1	cytoplasmic endogenous regulator of oxidative phosphorylation 1	0.66	3.44e-04	4.97e-02
Gamt	guanidinoacetate methyltransferase	2.33	3.44e-04	4.97e-02
Rasgrp3	RAS, guanyl releasing protein 3	1.08	3.48e-04	4.97e-02
Myl9	myosin, light polypeptide 9, regulatory	-1.26	3.49e-04	4.97e-02
Cebpg	CCAAT/enhancer binding protein (C/EBP), gamma	-1.13	3.49e-04	4.97e-02
1110038B12Rik	RIKEN cDNA 1110038B12 gene	-1.36	3.53e-04	4.97e-02
Pole4	polymerase (DNA-directed), epsilon 4 (p12 subunit)	-0.95	3.58e-04	4.97e-02
Cebpb	CCAAT/enhancer binding protein (C/EBP), beta	-1.33	3.59e-04	4.97e-02
Abca2	ATP-binding cassette, sub-family A (ABC1), member 2	1.57	3.63e-04	4.97e-02
Omg	oligodendrocyte myelin glycoprotein	0.92	3.63e-04	4.97e-02
Aplp1	amyloid beta (A4) precursor-like protein 1	1.21	3.68e-04	4.97e-02
Tppp	tubulin polymerization promoting protein	0.94	3.68e-04	4.97e-02
Tagln	transgelin	-1.76	3.73e-04	4.97e-02
Mast4	microtubule associated serine/threonine kinase family member 4	1.53	3.75e-04	4.97e-02
Rasl12	RAS-like, family 12	0.89	3.77e-04	4.97e-02
Serpinb1c	serine (or cysteine) peptidase inhibitor, clade B, member 1c	1.65	3.77e-04	4.97e-02

Table S4. Differentially expressed genes identified in the microglia obtained from mice treated with cuprizone for4 weeks compared with microglia obtained from control mice.

Symbol	Gene name	Log2 fold change	p-value	Adjusted p-value
Mmp12	matrix metallopeptidase 12	2.63	3.14E-07	5.52E-03
Lgals3	lectin, galactose binding, soluble 3	2.15	6.23E-07	5.52E-03
Fam20c	family with sequence similarity 20, member C	2.22	5.16E-06	2.73E-02
Gas2l3	growth arrest-specific 2 like 3	1.54	6.17E-06	2.73E-02
Cxcr4	chemokine (C-X-C motif) receptor 4	1.35	8.77E-06	3.11E-02
Clic4	chloride intracellular channel 4 (mitochondrial)	1.13	1.19E-05	3.43E-02
Clec7a	C-type lectin domain family 7, member a	2.47	1.44E-05	3.43E-02
Lpl	lipoprotein lipase	3.04	1.55E-05	3.43E-02
Cd69	CD69 antigen	1.27	1.82E-05	3.58E-02
Itgax	integrin alpha X	2.74	2.52E-05	4.34E-02

Rab7b	RAB7B, member RAS oncogene family	1.3 3	2.78E- 05	4.34E-02
Olr1	oxidized low density lipoprotein (lectin-like) receptor 1	1.8 3	2.94E- 05	4.34E-02

Table S5. Differentially expressed genes identified in the oligodendrocyte progenitor cells obtained from mice treated with cuprizone for 5 weeks compared with oligodendrocyte progenitor cells obtained from control mice. Due to the large number of differentially expressed genes only those with an adjusted p-value<0.001 are presented.

Symbol	Gene name	Log2 fold change	p-value	Adjusted p-value
Tagln2	*Mus musculus* transgelin 2 (Tagln2),mRNA [NM_178598]	5.16	1.79e-12	5.74e-08
Atf5	*Mus musculus* activating transcription factor 5 (Atf5), transcript variant 1,mRNA [NM_030693]	3.27	2.35e-11	3.76e-07
Slc7a1	*Mus musculus* solute carrier family 7(cationic amino acid transporter, y+ system), member 1 (Slc7a1), mRNA [NM_007513]	4.35	3.56e-11	3.80e-07
Col5a3	*Mus musculus* collagen, type V, alpha 3 (Col5a3), mRNA [NM_016919]	4.25	1.43e-10	1.15e-06
Gadd45b	*Mus musculus* growth arrest and DNA-damage-inducible 45 beta (Gadd45b), mRNA [NM_008655]	3.67	2.49e-10	1.60e-06
Cdkn1a	*Mus musculus* cyclin-dependent kinase inhibitor 1A (P21) (Cdkn1a), transcriptvariant 1, mRNA [NM_007669]	5.83	4.29e-10	2.29e-06
Ccnd1	*Mus musculus* cyclin D1 (Ccnd1), mRNA[NM_007631]	3.40	5.58e-10	2.38e-06
Trib3	*Mus musculus* tribbles homolog 3(Drosophila) (Trib3), mRNA [NM_175093]	7.23	5.93e-10	2.38e-06
Ppp1r15a	*Mus musculus* protein phosphatase 1, regulatory (inhibitor) subunit 15A (Ppp1r15a), mRNA [NM_008654]	2.96	7.51e-10	2.67e-06
Egr1	*Mus musculus* early growth response 1(Egr1), mRNA [NM_007913]	2.49	9.52e-10	3.05e-06
Nupr1	*Mus musculus* nuclear protein 1 (Nupr1),mRNA [NM_019738]	4.86	1.13e-09	3.22e-06
Ltbp4	*Mus musculus* latent transforming growthfactor beta binding protein 4 (Ltbp4), transcript variant 1, mRNA [NM_175641]	2.73	1.21e-09	3.22e-06
Bbc3	*Mus musculus* BCL2 binding component 3 (Bbc3), mRNA [NM_133234]	2.91	1.48e-09	3.64e-06
Fosb	*Mus musculus* FBJ osteosarcomaoncogene B (Fosb), mRNA [NM_008036]	2.54	3.51e-09	6.85e-06
Tubb2b	*Mus musculus* tubulin, beta 2B (Tubb2b), mRNA [NM_023716]	2.92	3.64e-09	6.85e-06
Socs3	*Mus musculus* suppressor of cytokine signaling 3 (Socs3), mRNA[NM_007707]	3.16	3.85e-09	6.85e-06
Vim	*Mus musculus* vimentin (Vim), mRNA [NM_011701]	3.44	3.21e-09	6.85e-06
Sox4	Transcription factor SOX-4 [Source:UniProtKB/Swiss-Prot;Acc:Q06831] [ENSMUST00000067230]	3.21	3.84e-09	6.85e-06
Kctd15	*Mus musculus* potassium channel tetramerisation domain containing 15 (Kctd15), mRNA [NM_146188]	2.95	5.25e-09	8.85e-06
Gadd45g	*Mus musculus* growth arrest and DNA- damage-inducible 45 gamma (Gadd45g), mRNA [NM_011817]	4.09	6.27e-09	1.01e-05
Tnfrsf12a	*Mus musculus* tumor necrosis factor receptor superfamily, member 12a (Tnfrsf12a), transcript variant 1, mRNA [NM_013749]	3.42	6.65e-09	1.01e-05
Asns	*Mus musculus* asparagine synthetase (Asns), mRNA [NM_012055]	4.47	7.35e-09	1.07e-05
Hist1h3d	*Mus musculus* histone cluster 1, H3d(Hist1h3d), mRNA [NM_178204]	1.78	8.52e-09	1.19e-05
Klf4	*Mus musculus* Kruppel-like factor 4 (gut) (Klf4), mRNA [NM_010637]	3.07	9.31e-09	1.24e-05

Lmo4	*Mus musculus* LIM domain only 4 (Lmo4), transcript variant 1, mRNA[NM_010723]	2.61	1.06e-08	1.35e-05
Tubb2a	*Mus musculus* tubulin, beta 2A (Tubb2a), mRNA [NM_009450]	2.52	1.10e-08	1.35e-05
Hmga1	*Mus musculus* high mobility group AT-hook 1 (Hmga1), transcript variant 1, mRNA [NM_016660]	2.66	1.20e-08	1.43e-05
Ptprz1	*Mus musculus* protein tyrosine phosphatase, receptor type Z, polypeptide1 (Ptprz1), mRNA [NM_001081306]	2.62	1.41e-08	1.52e-05
Gm12260	PREDICTED: *Mus musculus* similar tohistone H3 (LOC382523), mRNA [XM_905850]	2.01	1.46e-08	1.52e-05
Rtn1	*Mus musculus* reticulon 1 (Rtn1),transcript variant 1, mRNA [NM_153457]	3.15	1.47e-08	1.52e-05
Gm9315	PREDICTED: *Mus musculus* predicted gene, EG668714 (EG668714), mRNA[XM_001003263]	-3.25	1.69e-08	1.64e-05
Marcks	*Mus musculus* myristoylated alanine richprotein kinase C substrate (Marcks), mRNA [NM_008538]	3.84	1.79e-08	1.68e-05
Nfil3	*Mus musculus* nuclear factor, interleukin 3, regulated (Nfil3), mRNA[NM_017373]	2.10	1.86e-08	1.70e-05
Il33	*Mus musculus* interleukin 33 (Il33), transcript variant 1, mRNA[NM_001164724]	-1.81	2.24e-08	1.99e-05
Aen	*Mus musculus* apoptosis enhancingnuclease (Aen), transcript variant 1,mRNA [NM_026531]	2.47	2.59e-08	2.24e-05
Nrcam	*Mus musculus* neuron-glia-CAM-related cell adhesion molecule (Nrcam), transcript variant 1, mRNA [NM_176930]	2.55	2.81e-08	2.35e-05
Scrg1	*Mus musculus* scrapie responsive gene 1 (Scrg1), mRNA [NM_009136]	2.72	2.86e-08	2.35e-05
Serpinb1a	*Mus musculus* serine (or cysteine) peptidase inhibitor, clade B, member 1a (Serpinb1a), mRNA [NM_025429]	-1.88	3.04e-08	2.43e-05
Bcan	*Mus musculus* brevican (Bcan), transcript variant 1, mRNA [NM_007529]	3.27	3.35e-08	2.54e-05
Crip2	*Mus musculus* cysteine rich protein 2 (Crip2), mRNA [NM_024223]	2.10	3.41e-08	2.54e-05
Rcc2	*Mus musculus* regulator of chromosome condensation 2 (Rcc2), mRNA[NM_173867]	2.24	3.57e-08	2.60e-05
LOC634933	PREDICTED: *Mus musculus* similar to protein phosphatase 1, catalytic subunit(LOC634933), mRNA [XM_909811]	-2.90	3.81e-08	2.68e-05
Dnajb2	*Mus musculus* DnaJ (Hsp40) homolog,subfamily B, member 2 (Dnajb2), transcript variant 1, mRNA[NM_020266]	-1.85	3.85e-08	2.68e-05
Cmtm5	*Mus musculus* CKLF-like MARVELtransmembrane domain containing 5 (Cmtm5), mRNA [NM_026066]	-1.96	3.94e-08	2.68e-05
Cyp26b1	*Mus musculus* cytochrome P450, family26, subfamily b, polypeptide 1 (Cyp26b1), mRNA [NM_175475]	2.50	4.13e-08	2.75e-05
2610002J02Rik	*Mus musculus* RIKEN cDNA 2610002J02 gene (2610002J02Rik),mRNA [NM_001033134]	1.98	4.86e-08	3.18e-05
Fos	*Mus musculus* FBJ osteosarcoma oncogene (Fos), mRNA [NM_010234]	2.36	4.97e-08	3.19e-05
Abca1	*Mus musculus* ATP-binding cassette, sub-family A (ABC1), member 1(Abca1), mRNA [NM_013454]	2.24	5.28e-08	3.26e-05
Snhg1	*Mus musculus* small nucleolar RNA hostgene (non-protein coding) 1 (Snhg1), non-coding RNA [NR_002896]	1.93	5.34e-08	3.26e-05
Gm13889	*Mus musculus* predicted gene 13889 (Gm13889), mRNA [NM_001145034]	2.15	5.74e-08	3.38e-05
Itpr2	*Mus musculus* inositol 1,4,5-triphosphate receptor 2 (Itpr2), transcript variant 1,mRNA [NM_019923]	2.36	5.80e-08	3.38e-05

Btg1	*Mus musculus* B-cell translocation gene1, anti-proliferative (Btg1), mRNA [NM_007569]	3.03	6.03e-08	3.45e-05
Micall1	*Mus musculus* microtubule associatedmonoxygenase, calponin and LIM domain containing -like 1 (Micall1), mRNA [NM_177461]	-1.59	6.50e-08	3.65e-05
Tppp	*Mus musculus* tubulin polymerizationpromoting protein (Tppp), mRNA [NM_182839]	-1.53	6.77e-08	3.74e-05
Cpe	*Mus musculus* carboxypeptidase E (Cpe), mRNA [NM_013494]	1.88	6.99e-08	3.80e-05
H2-K1	*Mus musculus* histocompatibility 2, K1, K region (H2-K1), transcript variant 1,mRNA [NM_001001892]	2.11	7.26e-08	3.87e-05
Enoph1	*Mus musculus* enolase-phosphatase 1 (Enoph1), transcript variant 1, mRNA[NM_026421]	-1.63	7.37e-08	3.87e-05
Camk2n2	*Mus musculus* calcium/calmodulin- dependent protein kinase II inhibitor 2(Camk2n2), mRNA [NM_028420]	2.32	8.05e-08	4.11e-05
H2-D1	*Mus musculus* histocompatibility 2, D region locus 1 (H2-D1), mRNA [NM_010380]	2.84	8.08e-08	4.11e-05
Sgk2	*Mus musculus* serum/glucocorticoid regulated kinase 2 (Sgk2), mRNA[NM_013731]	-2.66	9.13e-08	4.47e-05
Ugt8a	*Mus musculus* UDP galactosyltransferase 8A (Ugt8a), mRNA [NM_011674]	-1.83	9.19e-08	4.47e-05
6330503K22Rik	*Mus musculus* RIKEN cDNA 6330503K22 gene (6330503K22Rik), mRNA [NM_182995]	-1.97	9.22e-08	4.47e-05
Msn	*Mus musculus* moesin (Msn), mRNA [NM_010833]	2.27	9.44e-08	4.51e-05
Gpr17	*Mus musculus* G protein-coupled receptor 17 (Gpr17), mRNA [NM_001025381]	2.93	9.61e-08	4.53e-05
H3f3b	*Mus musculus* H3 histone, family 3B (H3f3b), mRNA [NM_008211]	1.49	1.01e-07	4.55e-05
Anln	*Mus musculus* anillin, actin binding protein (Anln), mRNA [NM_028390]	-1.82	1.01e-07	4.55e-05
Tmeff1	*Mus musculus* transmembrane proteinwith EGF-like and two follistatin-likedomains 1 (Tmeff1), mRNA [NM_021436]	-1.77	1.02e-07	4.55e-05
Mical1	*Mus musculus* microtubule associated monoxygenase, calponin and LIM domain containing 1 (Mical1), transcript variant 1, mRNA [NM_138315]	2.04	1.00e-07	4.55e-05
S1pr5	*Mus musculus* sphingosine-1-phosphate receptor 5 (S1pr5), mRNA [NM_053190]	-1.79	1.13e-07	4.91e-05
Slc3a2	*Mus musculus* solute carrier family 3 (activators of dibasic and neutral aminoacid transport), member 2 (Slc3a2), transcript variant 2, mRNA [NM_008577]	1.76	1.25e-07	5.35e-05
Ephx1	*Mus musculus* epoxide hydrolase 1, microsomal (Ephx1), mRNA[NM_010145]	2.66	1.30e-07	5.48e-05
2410006H16Rik	*Mus musculus* RIKEN cDNA 2410006H16 gene (2410006H16Rik), non-coding RNA [NR_030738]	2.56	1.34e-07	5.48e-05
Chchd10	*Mus musculus* coiled-coil-helix-coiled-coil-helix domain containing 10 (Chchd10), mRNA [NM_175329]	2.85	1.35e-07	5.48e-05
Dusp6	*Mus musculus* dual specificity phosphatase 6 (Dusp6), mRNA[NM_026268]	2.49	1.35e-07	5.48e-05
Midn	*Mus musculus* midnolin (Midn), mRNA [NM_021565]	2.56	1.43e-07	5.53e-05
1810032O08Rik	*Mus musculus* RIKEN cDNA 1810032O08 gene (1810032O08Rik), transcript variant 3, non-coding RNA[NR_027821]	1.93	1.45e-07	5.53e-05
Trim47	*Mus musculus* tripartite motif-containing 47 (Trim47), mRNA [NM_172570]	2.15	1.42e-07	5.53e-05
3830612M24	*Mus musculus* 18 days pregnant adult female placenta and extra embryonic tissue cDNA, RIKEN full-length enrichedlibrary, clone:3830612M24 product:unclassifiable, full insert sequence. [AK028406]	2.88	1.43e-07	5.53e-05

Traf4	*Mus musculus* TNF receptor associated factor 4 (Traf4), mRNA [NM_009423]	2.47	1.44e-07	5.53e-05
Klf6	*Mus musculus* Kruppel-like factor 6 (Klf6), mRNA [NM_011803]	1.65	1.51e-07	5.71e-05
Adamtsl4	*Mus musculus* ADAMTS-like 4 (Adamtsl4), mRNA [NM_144899]	-2.48	1.59e-07	5.90e-05
Pppde2	*Mus musculus* PPPDE peptidase domain containing 2 (Pppde2), mRNA [NM_134095]	-2.17	1.60e-07	5.90e-05
Lmna	*Mus musculus* lamin A (Lmna), transcript variant 2, mRNA [NM_019390]	1.91	1.64e-07	5.90e-05
1500012F01Rik	*Mus musculus* RIKEN cDNA 1500012F01 gene (1500012F01Rik), mRNA [NM_001081005]	1.68	1.65e-07	5.90e-05
C4b	*Mus musculus* complement component4B (Childo blood group) (C4b), mRNA [NM_009780]	2.09	1.66e-07	5.90e-05
Prrg1	*Mus musculus* proline rich Gla (G-carboxyglutamic acid) 1 (Prrg1), transcript variant 1, mRNA [NM_027322]	-1.95	1.68e-07	5.90e-05
Efhd1	*Mus musculus* EF hand domaincontaining 1 (Efhd1), mRNA [NM_028889]	-1.93	1.75e-07	6.08e-05
Zfp703	*Mus musculus* zinc finger protein 703(Zfp703), transcript variant 2, mRNA [NM_001110508]	2.88	1.79e-07	6.17e-05
Aars	*Mus musculus* alanyl-tRNA synthetase (Aars), mRNA [NM_146217]	1.83	1.82e-07	6.22e-05
4930506M07Rik	*Mus musculus* RIKEN cDNA 4930506M07 gene (4930506M07Rik), transcript variant 2, mRNA[NM_175172]	-2.42	2.15e-07	7.10e-05
Ddit3	*Mus musculus* DNA-damage inducibletranscript 3 (Ddit3), mRNA [NM_007837]	2.26	2.15e-07	7.10e-05
Gzmm	*Mus musculus* granzyme M (lymphocytemet-ase 1) (Gzmm), mRNA [NM_008504]	1.78	2.35e-07	7.67e-05
Atf3	*Mus musculus* activating transcription factor 3 (Atf3), mRNA [NM_007498]	2.99	2.45e-07	7.92e-05
Pea15a	*Mus musculus* phosphoprotein enrichedin astrocytes 15A (Pea15a), transcript variant 2, mRNA [NM_011063]	-1.57	2.48e-07	7.92e-05
Sfxn3	*Mus musculus* sideroflexin 3 (Sfxn3), mRNA [NM_053197]	1.61	2.50e-07	7.92e-05
Prr5l	*Mus musculus* proline rich 5 like (Prr5l),transcript variant 2, mRNA [NM_175181]	-1.10	2.57e-07	8.06e-05
Stmn3	*Mus musculus* stathmin-like 3 (Stmn3),mRNA [NM_009133]	2.06	2.69e-07	8.38e-05
Padi2	*Mus musculus* peptidyl arginine deiminase, type II (Padi2), mRNA [NM_008812]	-1.75	2.74e-07	8.42e-05
Synj2	*Mus musculus* synaptojanin 2 (Synj2),transcript variant 3, mRNA [NM_011523]	-2.01	2.76e-07	8.42e-05
Tap2	*Mus musculus* transporter 2, ATP-binding cassette, sub-family B (MDR/TAP) (Tap2), mRNA [NM_011530]	2.09	3.10e-07	9.37e-05
B2m	*Mus musculus* beta-2 microglobulin (B2m), mRNA [NM_009735]	2.18	3.14e-07	9.39e-05
Sept7	*Mus musculus* septin 7 (Sept7), mRNA [NM_009859]	-1.23	3.30e-07	9.61e-05
1810041L15Rik	*Mus musculus* RIKEN cDNA 1810041L15 gene (1810041L15Rik), mRNA [NM_001163145]	2.79	3.38e-07	9.76e-05
Arap2	*Mus musculus* ArfGAP with RhoGAP domain, ankyrin repeat and PH domain 2(Arap2), mRNA [NM_178407]	1.73	3.54e-07	1.00e-04
Slco3a1	*Mus musculus* solute carrier organic anion transporter family, member 3a1 (Slco3a1), transcript variant 2, mRNA [NM_001038643]	-1.58	3.59e-07	1.00e-04

Egr2	*Mus musculus* early growth response 2 (Egr2), mRNA [NM_010118]	2.47	3.64e-07	1.00e-04
Brd2	*Mus musculus* bromodomain containing 2(Brd2), transcript variant 2, mRNA [NM_001025387]	1.30	3.64e-07	1.00e-04
Slc1a1	*Mus musculus* solute carrier family 1(neuronal/epithelial high affinity glutamate transporter, system Xag), member 1 (Slc1a1), mRNA [NM_009199]	2.43	3.65e-07	1.00e-04
Moxd1	*Mus musculus* monooxygenase, DBH-like 1 (Moxd1), mRNA [NM_021509]	4.18	3.58e-07	1.00e-04
Bnip3l	*Mus musculus* BCL2/adenovirus E1Binteracting protein 3-like (Bnip3l), mRNA [NM_009761]	-1.14	3.69e-07	1.00e-04
Epb4.1l3	*Mus musculus* erythrocyte protein band4.1-like 3 (Epb4.1l3), mRNA [NM_013813]	-1.74	4.05e-07	1.08e-04
Mt2	*Mus musculus* metallothionein 2 (Mt2), mRNA [NM_008630]	3.35	4.37e-07	1.15e-04
D16Ertd 472e	*Mus musculus* DNA segment, Chr 16,ERATO Doi 472, expressed (D16Ertd472e), mRNA [NM_025967]	-1.33	4.61e-07	1.19e-04
Hmgcs1	*Mus musculus* 3-hydroxy-3- methylglutaryl-Coenzyme A synthase 1 (Hmgcs1), mRNA [NM_145942]	-1.74	4.75e-07	1.22e-04
Kcnip3	*Mus musculus* Kv channel interacting protein 3, calsenilin (Kcnip3), transcriptvariant 2, mRNA [NM_001111331]	2.78	4.89e-07	1.23e-04
Chpf	*Mus musculus* chondroitin polymerizing factor (Chpf), transcript variant 2, mRNA [NM_001001565]	1.59	4.93e-07	1.23e-04
Eif4ebp1	*Mus musculus* eukaryotic translationinitiation factor 4E binding protein 1 (Eif4ebp1), mRNA [NM_007918]	3.88	4.94e-07	1.23e-04
Slc5a11	*Mus musculus* solute carrier family 5 (sodium/glucose cotransporter), member11 (Slc5a11), mRNA [NM_146198]	-1.96	5.27e-07	1.29e-04
Slc38a1	*Mus musculus* solute carrier family 38, member 1 (Slc38a1), transcript variant 1,mRNA [NM_134086]	2.12	5.50e-07	1.33e-04
Sort1	*Mus musculus* sortilin 1 (Sort1), mRNA [NM_019972]	-1.43	5.55e-07	1.34e-04
Aspa	*Mus musculus* aspartoacylase (Aspa), mRNA [NM_023113]	-1.49	5.68e-07	1.34e-04
Entpd5	*Mus musculus* ectonucleoside triphosphate diphosphohydrolase 5 (Entpd5), transcript variant 2, mRNA [NM_001026214]	-1.66	5.67e-07	1.34e-04
Ptprd	*Mus musculus* protein tyrosine phosphatase, receptor type, D (Ptprd),transcript variant b, mRNA [NM_011211]	-2.27	5.68e-07	1.34e-04
H2-Q7	*Mus musculus* histocompatibility 2, Q region locus 7 (H2-Q7), mRNA[NM_010394]	2.33	6.04e-07	1.39e-04
Gm7035	*Mus musculus* predicted gene 7035(Gm7035), non-coding RNA [NR_004446]	1.51	6.26e-07	1.43e-04
Nelf	*Mus musculus* nasal embryonic LHRH factor (Nelf), transcript variant 1, mRNA [NM_001039386]	-1.15	7.13e-07	1.61e-04
Dock6	*Mus musculus* dedicator of cytokinesis 6 (Dock6), mRNA [NM_177030]	1.59	7.26e-07	1.62e-04
Cebpb	*Mus musculus* CCAAT/enhancer binding protein (C/EBP), beta (Cebpb), mRNA [NM_009883]	2.33	7.27e-07	1.62e-04
Gdf1	*Mus musculus* growth differentiationfactor 1 (Gdf1), transcript variant 2, mRNA [NM_008107]	1.61	7.42e-07	1.64e-04
Gpt	*Mus musculus* glutamic pyruvic transaminase, soluble (Gpt), mRNA [NM_182805]	-1.77	7.75e-07	1.70e-04
Crlf2	*Mus musculus* cytokine receptor-likefactor 2 (Crlf2),	1.81	7.87e-07	1.72e-04

	transcript variant 1, mRNA [NM_001164735]			
Snhg12	*Mus musculus* small nucleolar RNA hostgene 12 (Snhg12), non-coding RNA [NR_029468]	1.80	8.03e-07	1.74e-04
Jph4	*Mus musculus* junctophilin 4 (Jph4), transcript variant a, mRNA[NM_177049]	-3.81	8.19e-07	1.76e-04
H2-T23	*Mus musculus* histocompatibility 2, Tregion locus 23 (H2-T23), mRNA [NM_010398]	1.92	8.49e-07	1.81e-04
Dip2a	*Mus musculus* DIP2 disco-interactingprotein 2 homolog A (Drosophila) (Dip2a), mRNA [NM_001081419]	-1.40	8.52e-07	1.81e-04
Foxn3	*Mus musculus* forkhead box N3 (Foxn3), mRNA [NM_183186]	-1.41	8.97e-07	1.89e-04
Tmem176b	*Mus musculus* transmembrane protein 176B (Tmem176b), transcript variant 1, mRNA [NM_023056]	2.76	9.23e-07	1.93e-04
Gars	*Mus musculus* glycyl-tRNA synthetase(Gars), mRNA [NM_180678]	1.05	9.83e-07	2.05e-04
Jam3	*Mus musculus* junction adhesion molecule 3 (Jam3), mRNA [NM_023277]	-0.98	1.07e-06	2.20e-04
Atp1b3	*Mus musculus* ATPase, Na+/K+ transporting, beta 3 polypeptide (Atp1b3),mRNA [NM_007502]	-1.27	1.07e-06	2.20e-04
Fzd1	*Mus musculus* frizzled homolog 1(Drosophila) (Fzd1), mRNA [NM_021457]	2.26	1.12e-06	2.28e-04
Gdf15	*Mus musculus* growth differentiation factor 15 (Gdf15), mRNA [NM_011819]	5.28	1.12e-06	2.28e-04
Rab34	*Mus musculus* RAB34, member of RAS oncogene family (Rab34), transcriptvariant 1, mRNA [NM_033475]	1.69	1.13e-06	2.28e-04
Eml2	*Mus musculus* echinoderm microtubuleassociated protein like 2 (Eml2), transcript variant 1, mRNA [NM_028153]	-1.66	1.19e-06	2.39e-04
Rasl12	*Mus musculus* RAS-like, family 12 (Rasl12), transcript variant 1, mRNA[NM_001033158]	-1.93	1.21e-06	2.40e-04
Rhoc	*Mus musculus* ras homolog gene family, member C (Rhoc), mRNA [NM_007484]	1.44	1.23e-06	2.42e-04
Itih3	*Mus musculus* inter-alpha trypsin inhibitor, heavy chain 3 (Itih3), mRNA [NM_008407]	-2.29	1.32e-06	2.57e-04
Nipa1	*Mus musculus* non imprinted in Prader-Willi/Angelman syndrome 1 homolog (human) (Nipa1), mRNA [NM_153578]	-1.76	1.31e-06	2.57e-04
Cars	*Mus musculus* cysteinyl-tRNA synthetase (Cars), mRNA [NM_013742]	1.43	1.35e-06	2.60e-04
Daam1	*Mus musculus* dishevelled associated activator of morphogenesis 1 (Daam1),transcript variant 1, mRNA [NM_026102]	-1.85	1.36e-06	2.60e-04
4930506C21Rik	*Mus musculus* adult male testis cDNA, RIKEN full-length enriched library, clone:4930506C21 product:unclassifiable, full insert sequence. [AK015714] clone:4930506C21	-2.15	1.43e-06	2.72e-04
Ctnna2	*Mus musculus* catenin (cadherin associated protein), alpha 2 (Ctnna2),transcript variant 2, mRNA [NM_009819]	-1.29	1.46e-06	2.76e-04
Cebpd	*Mus musculus* CCAAT/enhancer bindingprotein (C/EBP), delta (Cebpd), mRNA [NM_007679]	2.49	1.49e-06	2.77e-04
Opalin	*Mus musculus* oligodendrocytic myelinparanodal and inner loop protein (Opalin), mRNA [NM_153520]	-1.99	1.50e-06	2.77e-04
Ciapin1	*Mus musculus* cytokine induced apoptosis inhibitor 1 (Ciapin1), mRNA [NM_134141]	1.38	1.50e-06	2.77e-04
Ptpro	*Mus musculus* protein tyrosine phosphatase, receptor type, O (Ptpro),transcript variant 1, mRNA [NM_011216]	1.77	1.52e-06	2.77e-04

Trim59	*Mus musculus* tripartite motif-containing 59 (Trim59), mRNA [NM_025863]	-1.11	1.52e-06	2.77e-04
Gadd45a	*Mus musculus* growth arrest and DNA- damage-inducible 45 alpha (Gadd45a),mRNA [NM_007836]	2.07	1.53e-06	2.77e-04
Cdkn1c	*Mus musculus* cyclin-dependent kinaseinhibitor 1C (P57) (Cdkn1c), transcript variant 2, mRNA [NM_009876]	1.62	1.58e-06	2.84e-04
Rnf13	*Mus musculus* ring finger protein 13 (Rnf13), transcript variant 1, mRNA[NM_001113413]	-1.09	1.62e-06	2.88e-04
Trim2	*Mus musculus* tripartite motif-containing 2 (Trim2), mRNA [NM_030706]	-0.91	1.61e-06	2.88e-04
Adamts1	*Mus musculus* a disintegrin-like and metallopeptidase (reprolysin type) withthrombospondin type 1 motif, 1 (Adamts1), mRNA [NM_009621]	1.36	1.63e-06	2.88e-04
Chmp7	*Mus musculus* CHMP family, member 7 (Chmp7), mRNA [NM_134078]	-1.27	1.64e-06	2.89e-04
Fa2h	*Mus musculus* fatty acid 2-hydroxylase (Fa2h), mRNA [NM_178086]	-1.43	1.69e-06	2.96e-04
Dos	*Mus musculus* downstream of Stk11 (Dos), mRNA [NM_015761]	1.52	1.80e-06	3.14e-04
Vps37b	*Mus musculus* vacuolar protein sorting 37B (yeast) (Vps37b), mRNA[NM_177876]	1.52	1.83e-06	3.17e-04
Sema5a	*Mus musculus* sema domain, seven thrombospondin repeats (type 1 and type 1-like), transmembrane domain (TM) andshort cytoplasmic domain, (semaphorin) 5A (Sema5a), mRNA [NM_009154]	2.11	1.84e-06	3.17e-04
Pmp22	*Mus musculus* peripheral myelin protein 22 (Pmp22), mRNA [NM_008885]	-1.34	1.87e-06	3.20e-04
Rufy3	*Mus musculus* RUN and FYVE domain containing 3 (Rufy3), mRNA[NM_027530]	-1.05	1.88e-06	3.20e-04
Ier5l	*Mus musculus* immediate early response 5-like (Ier5l), mRNA [NM_030244]	2.96	1.89e-06	3.21e-04
Slc25a13	*Mus musculus* solute carrier family 25 (mitochondrial carrier, adenine nucleotidetranslocator), member 13 (Slc25a13), nuclear gene encoding mitochondrialprotein, mRNA [NM_015829]	-1.83	1.93e-06	3.24e-04
Ldlr	*Mus musculus* low density lipoprotein receptor (Ldlr), mRNA [NM_010700]	-1.61	2.00e-06	3.34e-04
6430527G	*Mus musculus* RIKEN cDNA	1.53	2.08e-06	3.45e-04
18Rik	6430527G18 gene (6430527G18Rik), mRNA [NM_145836]			
Timp3	*Mus musculus* tissue inhibitor of metalloproteinase 3 (Timp3), mRNA [NM_011595]	2.30	2.09e-06	3.45e-04
Phyhd1	*Mus musculus* phytanoyl-CoA dioxygenase domain containing 1 (Phyhd1), mRNA [NM_172267]	2.29	2.11e-06	3.47e-04
Il18	*Mus musculus* interleukin 18 (Il18),mRNA [NM_008360]	-1.21	2.13e-06	3.48e-04
Pcdhga9	*Mus musculus* protocadherin gammasubfamily A, 9 (Pcdhga9), mRNA [NM_033592]	1.25	2.17e-06	3.53e-04
Aldh3b1	*Mus musculus* aldehyde dehydrogenase 3family, member B1 (Aldh3b1), mRNA [NM_026316]	-1.37	2.20e-06	3.57e-04
Shroom2	*Mus musculus* shroom family member 2 (Shroom2), mRNA [NM_172441]	-1.24	2.24e-06	3.57e-04
LOC683 95	PREDICTED: *Mus musculus* RIKENcDNA 0610037M15 gene, transcript variant 2 (0610037M15Rik), mRNA [XM_903697]	2.30	2.24e-06	3.57e-04
Sesn2	*Mus musculus* sestrin 2 (Sesn2), mRNA [NM_144907]	1.34	2.25e-06	3.57e-04
Chac1	*Mus musculus* ChaC, cation transport regulator-like 1 (E. coli) (Chac1), mRNA [NM_026929]	3.98	2.23e-06	3.57e-04
Fyn	*Mus musculus* Fyn proto-oncogene (Fyn), transcript variant 1, mRNA[NM_001122893]	1.63	2.32e-06	3.65e-04
Sertad1	*Mus musculus* SERTA domaincontaining 1 (Sertad1), mRNA	2.02	2.37e-06	3.69e-04

	[NM_018820]			
Piga	*Mus musculus* phosphatidylinositolglycan anchor biosynthesis, class A (Piga), mRNA [NM_011081]	-1.72	2.38e-06	3.69e-04
Bcat1	*Mus musculus* branched chain aminotransferase 1, cytosolic (Bcat1),transcript variant 2, mRNA [NM_007532]	1.47	2.40e-06	3.70e-04
Erbb2ip	*Mus musculus* Erbb2 interacting protein(Erbb2ip), transcript variant 1, mRNA [NM_001005868]	-1.28	2.44e-06	3.72e-04
Thyn1	*Mus musculus* thymocyte nuclear protein 1 (Thyn1), mRNA [NM_144543]	1.09	2.44e-06	3.72e-04
Cntn1	*Mus musculus* contactin 1 (Cntn1), transcript variant 1, mRNA[NM_001159647]	1.33	2.51e-06	3.80e-04
Rcbtb1	*Mus musculus* regulator of chromosomecondensation (RCC1) and BTB (POZ) domain containing protein 1 (Rcbtb1), mRNA [NM_027764]	-1.34	2.52e-06	3.80e-04
Arsg	*Mus musculus* arylsulfatase G (Arsg),transcript variant 1, mRNA [NM_028710]	-0.95	2.52e-06	3.80e-04
Per1	*Mus musculus* period homolog 1 (Drosophila) (Per1), transcript variant 1,mRNA [NM_011065]	2.21	2.56e-06	3.84e-04
Cdk5	*Mus musculus* cyclin-dependent kinase 5 (Cdk5), mRNA [NM_007668]	-2.21	2.57e-06	3.84e-04
Lap3	*Mus musculus* leucine aminopeptidase 3 (Lap3), mRNA [NM_024434]	-1.06	2.60e-06	3.86e-04
Odc1	*Mus musculus* ornithine decarboxylase,structural 1 (Odc1), mRNA [NM_013614]	1.65	2.65e-06	3.91e-04
Cdc42bpa	*Mus musculus* CDC42 binding protein kinase alpha (Cdc42bpa), mRNA [NM_001033285]	-1.27	2.70e-06	3.98e-04
Ninj2	*Mus musculus* ninjurin 2 (Ninj2), mRNA [NM_016718]	-2.65	2.73e-06	4.00e-04
Cotl1	*Mus musculus* coactosin-like 1 (Dictyostelium) (Cotl1), mRNA [NM_028071]	2.14	2.75e-06	4.00e-04
Atpgd1	*Mus musculus* ATP-grasp domain containing 1 (Atpgd1), mRNA[NM_134148]	-3.23	2.80e-06	4.04e-04
Dusp1	*Mus musculus* dual specificity phosphatase 1 (Dusp1), mRNA [NM_013642]	1.81	2.80e-06	4.04e-04
Tuba1c	*Mus musculus* tubulin, alpha 1C (Tuba1c), mRNA [NM_009448]	2.25	2.85e-06	4.07e-04
Hhip	*Mus musculus* Hedgehog-interacting protein (Hhip), mRNA [NM_020259]	-1.39	2.84e-06	4.07e-04
Cyfip2	*Mus musculus* cytoplasmic FMR1 interacting protein 2 (Cyfip2), mRNA [NM_133769]	1.48	2.87e-06	4.08e-04
Bmp4	*Mus musculus* bone morphogenetic protein 4 (Bmp4), mRNA [NM_007554]	2.32	2.88e-06	4.08e-04
Hexim1	*Mus musculus* hexamethylene bis- acetamide inducible 1 (Hexim1), mRNA [NM_138753]	1.55	2.94e-06	4.13e-04
Rps2	*Mus musculus* ribosomal protein S2 (Rps2), mRNA [NM_008503]	0.96	3.02e-06	4.22e-04
Plec1	*Mus musculus* plectin 1 (Plec1), transcript variant 13, mRNA[NM_001163540]	2.19	3.09e-06	4.26e-04
Mthfd2	*Mus musculus* methylenetetrahydrofolatedehydrogenase (NAD+ dependent), methenyltetrahydrofolate cyclohydrolase(Mthfd2), nuclear gene encoding mitochondrial protein, mRNA [NM_008638]	1.50	3.07e-06	4.26e-04
Cnksr3	*Mus musculus* Cnksr family member 3 (Cnksr3), mRNA [NM_172546]	1.79	3.10e-06	4.26e-04
Gale	*Mus musculus* galactose-4-epimerase, UDP (Gale), mRNA [NM_178389]	1.37	3.09e-06	4.26e-04
Gm7159	PREDICTED: *Mus musculus* predicted gene, EG635497 (EG635497), misc RNA[XR_002030]	1.19	3.13e-06	4.26e-04

5730469 M10Rik	*Mus musculus* RIKEN cDNA 5730469M10 gene (5730469M10Rik), mRNA [NM_027464]	-1.59	3.13e-06	4.26e-04
Nudt4	*Mus musculus* nudix (nucleoside diphosphate linked moiety X)-type motif 4 (Nudt4), mRNA [NM_027722]	-1.40	3.14e-06	4.26e-04
Dpysl4	*Mus musculus* dihydropyrimidinase-like 4 (Dpysl4), mRNA [NM_011993]	2.78	3.16e-06	4.28e-04
Evl	*Mus musculus* Ena-vasodilator stimulated phosphoprotein (Evl), transcript variant 1,mRNA [NM_001163394]	1.41	3.29e-06	4.41e-04
A230001 M10Rik	*Mus musculus* adult male hypothalamuscDNA, RIKEN full-length enriched library, clone:A230012K17 product:unclassifiable, full insert sequence. [AK038444]	-1.58	3.37e-06	4.49e-04
Hopx	*Mus musculus* HOP homeobox (Hopx),transcript variant 1, mRNA [NM_175606]	-1.64	3.41e-06	4.51e-04
Anxa2	*Mus musculus* annexin A2 (Anxa2), mRNA [NM_007585]	2.48	3.40e-06	4.51e-04
Rell1	*Mus musculus* RELT-like 1 (Rell1), mRNA [NM_145923]	1.24	3.44e-06	4.53e-04
Limch1	*Mus musculus* LIM and calponin homology domains 1 (Limch1), mRNA [NM_001001980]	-1.36	3.50e-06	4.55e-04
Jam2	*Mus musculus* junction adhesion molecule 2 (Jam2), mRNA [NM_023844]	1.41	3.49e-06	4.55e-04
Gm4892	PREDICTED: *Mus musculus* similar toQM protein (LOC638133), mRNA [XM_914040]	0.78	3.54e-06	4.59e-04
Zbtb7b	*Mus musculus* zinc finger and BTB domain containing 7B (Zbtb7b), mRNA[NM_009565]	1.24	3.57e-06	4.61e-04
Smtnl2	*Mus musculus* smoothelin-like 2 (Smtnl2), mRNA [NM_177776]	-2.17	3.59e-06	4.61e-04
Gm8432	PREDICTED: *Mus musculus* predicted gene, EG667040 (EG667040), misc RNA [XR_001962]	1.62	3.65e-06	4.68e-04
Cmtm3	*Mus musculus* CKLF-like MARVELtransmembrane domain containing 3 (Cmtm3), mRNA [NM_024217]	1.64	3.67e-06	4.68e-04
Emp1	*Mus musculus* epithelial membrane protein 1 (Emp1), mRNA [NM_010128]	3.45	3.68e-06	4.68e-04
LOC100 047340	PREDICTED: *Mus musculus* hypothetical protein LOC100047340(LOC100047340), mRNA [XM_001477942]	1.77	3.74e-06	4.68e-04
9630013A 20Rik	*Mus musculus* RIKEN cDNA 9630013A20 gene (9630013A20Rik), non-coding RNA [NR_015539]	2.60	3.74e-06	4.68e-04
Txnip	*Mus musculus* thioredoxin interactingprotein (Txnip), transcript variant 1, mRNA [NM_001009935]	1.58	3.71e-06	4.68e-04
Stk40	*Mus musculus* serine/threonine kinase 40(Stk40), transcript variant 1, mRNA [NM_001145827]	1.56	3.74e-06	4.68e-04
Hspa9	*Mus musculus* heat shock protein 9(Hspa9), nuclear gene encoding mitochondrial protein, mRNA [NM_010481]	1.05	3.77e-06	4.70e-04
Gprc5b	*Mus musculus* G protein-coupled receptor, family C, group 5, member B (Gprc5b), mRNA [NM_022420]	-0.97	3.80e-06	4.72e-04
Glod4	*Mus musculus* glyoxalase domain containing 4 (Glod4), mRNA[NM_026029]	-1.23	3.83e-06	4.73e-04
Nmral1	*Mus musculus* NmrA-like family domaincontaining 1 (Nmral1), mRNA [NM_026393]	-1.65	3.85e-06	4.73e-04
Mrps18b	*Mus musculus* mitochondrial ribosomalprotein S18B (Mrps18b), nuclear gene encoding mitochondrial protein, mRNA[NM_025878]	1.24	3.85e-06	4.73e-04
Grm3	*Mus musculus* glutamate receptor,metabotropic 3 (Grm3), mRNA [NM_181850]	-1.79	3.87e-06	4.73e-04

Serpina3n	*Mus musculus* serine (or cysteine) peptidase inhibitor, clade A, member 3N (Serpina3n), mRNA [NM_009252]	2.30	3.90e-06	4.74e-04
Ppp1r14b	*Mus musculus* protein phosphatase 1, regulatory (inhibitor) subunit 14B (Ppp1r14b), mRNA [NM_008889]	1.74	3.91e-06	4.74e-04
Prr18	*Mus musculus* proline rich region 18(Prr18), transcript variant 1, mRNA [NM_178774]	-1.02	3.95e-06	4.75e-04
Irf1	*Mus musculus* interferon regulatoryfactor 1 (Irf1), transcript variant 1, mRNA [NM_008390]	1.82	3.98e-06	4.77e-04
Tle6	*Mus musculus* transducin-like enhancerof split 6, homolog of Drosophila E(spl) (Tle6), mRNA [NM_053254]	1.45	3.99e-06	4.77e-04
Angptl6	*Mus musculus* angiopoietin-like 6 (Angptl6), mRNA [NM_145154]	1.76	4.09e-06	4.86e-04
S100a6	*Mus musculus* S100 calcium binding protein A6 (calcyclin) (S100a6), mRNA [NM_011313]	2.33	4.11e-06	4.87e-04
Lgals3	*Mus musculus* lectin, galactose binding,soluble 3 (Lgals3), transcript variant 1, mRNA [NM_001145953]	2.76	4.16e-06	4.90e-04
B9d2	*Mus musculus* B9 protein domain 2 (B9d2), mRNA [NM_172148]	1.27	4.24e-06	4.96e-04
Dynll2	*Mus musculus* dynein light chain LC8-type 2 (Dynll2), transcript variant 1, mRNA [NM_026556]	1.28	4.29e-06	4.98e-04
Clmn	*Mus musculus* calmin (Clmn), transcript variant 1, mRNA [NM_053155]	-1.73	4.32e-06	4.99e-04
Ppp1cc	*Mus musculus* protein phosphatase 1, catalytic subunit, gamma isoform(Ppp1cc), mRNA [NM_013636]	-3.30	4.33e-06	4.99e-04
Rnf141	RING finger protein 141 (Zinc finger protein 230) [Source:UniProtKB/Swiss-Prot;Acc:Q99MB7] [ENSMUST00000106682]	-1.11	4.49e-06	5.15e-04
Pdlim1	*Mus musculus* PDZ and LIM domain 1 (elfin) (Pdlim1), mRNA [NM_016861]	-1.46	4.55e-06	5.20e-04
Gm5100	PREDICTED: *Mus musculus* predicted gene, EG329126 (EG329126), misc RNA[XR_001880]	1.13	4.62e-06	5.27e-04
Sv2a	*Mus musculus* synaptic vesicle glycoprotein 2 a (Sv2a), mRNA [NM_022030]	-1.37	4.67e-06	5.29e-04
Ermn	*Mus musculus* ermin, ERM-like protein (Ermn), mRNA [NM_029972]	-1.39	4.70e-06	5.29e-04
LOC100 047749	PREDICTED: *Mus musculus* similar tocAMP-specific cyclic nucleotide phosphodiesterase PDE8; MMPDE8 (LOC100047749), mRNA [XM_001478817]	-1.25	4.70e-06	5.29e-04
Cntf	*Mus musculus* ciliary neurotrophic factor (Cntf), mRNA [NM_170786]	1.69	4.74e-06	5.29e-04
Gm11230	PREDICTED: *Mus musculus* similar toribosomal protein (LOC100039979), mRNA [XM_001474183]	1.22	4.76e-06	5.29e-04
Gjc2	*Mus musculus* gap junction protein, gamma 2 (Gjc2), transcript variant 2,mRNA [NM_175452]	-1.80	4.77e-06	5.29e-04
Srcin1	*Mus musculus* SRC kinase signalinginhibitor 1 (Srcin1), mRNA [NM_018873]	-1.69	4.99e-06	5.52e-04
RP23-480B19.1 0	PREDICTED: *Mus musculus* similar to histone 2a, transcript variant 2 (Rp23-480b19.10), mRNA [XM_978341]	2.48	5.05e-06	5.56e-04
Rasal1	*Mus musculus* RAS protein activator like 1 (GAP1 like) (Rasal1), mRNA[NM_013832]	-2.13	5.37e-06	5.83e-04
Gm4838	PREDICTED: *Mus musculus* predictedgene, EG225416 (EG225416), mRNA [XM_140295]	1.57	5.36e-06	5.83e-04
Btg2	*Mus musculus* B-cell translocation gene2, anti-proliferative (Btg2), mRNA	1.55	5.36e-06	5.83e-04

	[NM_007570]			
Gm10653	*Mus musculus* predicted gene 10653(Gm10653), non-coding RNA [NR_003965]	0.97	5.40e-06	5.83e-04
Sepx1	*Mus musculus* selenoprotein X 1 (Sepx1), mRNA [NM_013759]	-1.07	5.42e-06	5.83e-04
Gm7204	PREDICTED: *Mus musculus* predictedgene, EG637273, transcript variant 1 (EG637273), mRNA [XM_917437]	-1.36	5.48e-06	5.86e-04
Btg3	*Mus musculus* B-cell translocation gene 3 (Btg3), mRNA [NM_009770]	1.46	5.49e-06	5.86e-04
Odz4	*Mus musculus* odd Oz/ten-m homolog 4 (Drosophila) (Odz4), mRNA[NM_011858]	1.68	5.60e-06	5.97e-04
Hspb1	*Mus musculus* heat shock protein 1 (Hspb1), mRNA [NM_013560]	1.62	5.66e-06	5.99e-04
Susd4	*Mus musculus* sushi domain containing 4 (Susd4), mRNA [NM_144796]	1.92	5.67e-06	5.99e-04
Sh3bp5l	*Mus musculus* SH3 binding domain protein 5 like (Sh3bp5l), transcript variant1, mRNA [NM_001161338]	-1.01	5.72e-06	6.03e-04
Sc4mol	*Mus musculus* sterol-C4-methyl oxidase-like (Sc4mol), mRNA [NM_025436]	-1.43	5.78e-06	6.07e-04
Rftn1	*Mus musculus* raftlin lipid raft linker 1 (Rftn1), mRNA [NM_181397]	-2.10	5.85e-06	6.12e-04
Gm8420	PREDICTED: *Mus musculus* similar toribosomal protein L15 (LOC667014), mRNA [XM_001473655]	0.97	5.89e-06	6.15e-04
Ifrd1	*Mus musculus* interferon-related developmental regulator 1 (Ifrd1), mRNA [NM_013562]	1.19	6.06e-06	6.29e-04
Tmem98	*Mus musculus* transmembrane protein 98 (Tmem98), mRNA [NM_029537]	-1.44	6.07e-06	6.29e-04
Rps19	*Mus musculus* ribosomal protein S19 (Rps19), mRNA [NM_023133]	1.62	6.12e-06	6.31e-04
Iglon5	*Mus musculus* IgLON family member 5 (Iglon5), mRNA [NM_001164518]	2.01	6.39e-06	6.53e-04
Pla2g4a	*Mus musculus* phospholipase A2, group IVA (cytosolic, calcium-dependent)(Pla2g4a), mRNA [NM_008869]	-1.28	6.40e-06	6.53e-04
Mthfd1l	*Mus musculus* methylenetetrahydrofolatedehydrogenase (NADP+ dependent) 1- like (Mthfd1l), nuclear gene encoding mitochondrial protein, transcript variant 2, mRNA [NM_172308]	1.39	6.43e-06	6.54e-04
Sdpr	*Mus musculus* serum deprivation response (Sdpr), mRNA [NM_138741]	-1.31	6.45e-06	6.54e-04
Scpep1	*Mus musculus* serine carboxypeptidase 1 (Scpep1), mRNA [NM_029023]	1.41	6.53e-06	6.60e-04
Kcna1	*Mus musculus* potassium voltage-gatedchannel, shaker-related subfamily, member 1 (Kcna1), mRNA [NM_010595]	-1.39	6.67e-06	6.72e-04
Gm2590	PREDICTED: *Mus musculus* hypothetical protein LOC100040086(LOC100040086), mRNA [XM_001474060]	-2.04	6.69e-06	6.72e-04
Ctss	*Mus musculus* cathepsin S (Ctss), mRNA [NM_021281]	2.84	6.84e-06	6.85e-04
Ppp2r2c	*Mus musculus* protein phosphatase 2 (formerly 2A), regulatory subunit B (PR52), gamma isoform (Ppp2r2c), mRNA [NM_172994]	-1.54	6.91e-06	6.90e-04
Pcgf5	*Mus musculus* polycomb group ring finger 5 (Pcgf5), mRNA [NM_029508]	1.47	6.96e-06	6.93e-04
Tet1	*Mus musculus* tet oncogene 1 (Tet1), mRNA [NM_027384]	-1.55	7.00e-06	6.95e-04
Aif1l	*Mus musculus* allograft inflammatoryfactor 1-like (Aif1l), mRNA [NM_145144]	-1.42	7.03e-06	6.96e-04
Aprt	*Mus musculus* adenine phosphoribosyl transferase (Aprt), mRNA [NM_009698]	1.65	7.15e-06	7.05e-04
Prom1	*Mus musculus* prominin 1 (Prom1),transcript variant 2,	2.02	7.21e-06	7.09e-04

	mRNA [NM_001163577]			
1190002H23Rik	*Mus musculus* RIKEN cDNA 1190002H23 gene (1190002H23Rik), mRNA [NM_025427]	1.99	7.28e-06	7.13e-04
Hhatl	*Mus musculus* hedgehog acyltransferase-like (Hhatl), transcript variant 1, mRNA [NM_029095]	-2.17	7.46e-06	7.29e-04
Tsc22d3	*Mus musculus* TSC22 domain family, member 3 (Tsc22d3), transcript variant 1, mRNA [NM_001077364]	-1.56	7.73e-06	7.53e-04
Rhoj	*Mus musculus* ras homolog gene family, member J (Rhoj), mRNA [NM_023275]	1.39	7.77e-06	7.54e-04
H2-Q6	*Mus musculus* histocompatibility 2, Qregion locus 6 (H2-Q6), mRNA [NM_207648]	2.19	7.79e-06	7.54e-04
Pacsin3	*Mus musculus* protein kinase C andcasein kinase substrate in neurons 3 (Pacsin3), mRNA [NM_028733]	-1.67	7.91e-06	7.61e-04
Ano4	*Mus musculus* anoctamin 4 (Ano4), mRNA [NM_178773]	-1.34	7.93e-06	7.61e-04
Ppp1r14a	*Mus musculus* protein phosphatase 1,regulatory (inhibitor) subunit 14A (Ppp1r14a), mRNA [NM_026731]	-1.34	8.14e-06	7.73e-04
Rpl10a	*Mus musculus* ribosomal protein L10A (Rpl10a), mRNA [NM_011287]	0.94	8.16e-06	7.73e-04
Cept1	*Mus musculus* choline/ethanolaminephosphotransferase1 (Cept1), mRNA [NM_133869]	-0.91	8.18e-06	7.73e-04
Fam171a1	*Mus musculus* family with sequence similarity 171, member A1 (Fam171a1),mRNA [NM_001081161]	-1.28	8.39e-06	7.87e-04
Pcdh10	*Mus musculus* protocadherin 10 (Pcdh10), transcript variant 2, mRNA [NM_001098172]	1.31	8.40e-06	7.87e-04
Sema4f	*Mus musculus* sema domain, immunoglobulin domain (Ig), TM domain, and short cytoplasmic domain(Sema4f), transcript variant 1, mRNA [NM_011350]	1.42	8.45e-06	7.90e-04
Tnr	*Mus musculus* tenascin R (Tnr), mRNA [NM_022312]	1.96	8.59e-06	7.98e-04
Il17rb	*Mus musculus* interleukin 17 receptor B (Il17rb), mRNA [NM_019583]	-1.48	8.67e-06	7.99e-04
Expi	*Mus musculus* extracellular proteinase inhibitor (Expi), mRNA [NM_007969]	-2.55	8.63e-06	7.99e-04
Psen2	*Mus musculus* presenilin 2 (Psen2), transcript variant 2, mRNA[NM_001128605]	-1.22	8.69e-06	7.99e-04
Prdx1	*Mus musculus* peroxiredoxin 1 (Prdx1), mRNA [NM_011034]	-1.41	8.72e-06	7.99e-04
Epas1	*Mus musculus* endothelial PAS domain protein 1 (Epas1), mRNA [NM_010137]	-1.16	8.73e-06	7.99e-04
Zfp622	*Mus musculus* zinc finger protein 622 (Zfp622), mRNA [NM_144523]	0.99	8.81e-06	8.04e-04
Rps18	*Mus musculus* ribosomal protein S18 (Rps18), mRNA [NM_011296]	1.22	8.91e-06	8.11e-04
Abcb10	*Mus musculus* ATP-binding cassette, sub-family B (MDR/TAP), member 10(Abcb10), nuclear gene encoding mitochondrial protein, mRNA [NM_019552]	-1.25	8.93e-06	8.11e-04
Ecel1	*Mus musculus* endothelin converting enzyme-like 1 (Ecel1), mRNA [NM_021306]	2.31	9.09e-06	8.15e-04
Tubb2c	*Mus musculus* tubulin, beta 2C (Tubb2c), mRNA [NM_146116]	1.05	9.07e-06	8.15e-04
Tubb5	*Mus musculus* tubulin, beta 5 (Tubb5), mRNA [NM_011655]	1.37	9.08e-06	8.15e-04
Plcl1	*Mus musculus* phospholipase C-like 1 (Plcl1), mRNA [NM_001114663]	-1.26	9.13e-06	8.15e-04
Myh14	*Mus musculus* myosin, heavy polypeptide 14 (Myh14), mRNA [NM_028021]	-1.41	9.14e-06	8.15e-04

Rassf2	*Mus musculus* Ras association (RalGDS/AF-6) domain family member 2<br>(Rassf2), mRNA [NM_175445]	-0.91	9.17e-06	8.16e-04
Sc5d	*Mus musculus* sterol-C5-desaturase (fungal ERG3, delta-5-desaturase) homolog (S. cerevisae) (Sc5d), mRNA [NM_172769]	-1.36	9.32e-06	8.27e-04
Crlf3	*Mus musculus* cytokine receptor-like<br>factor 3 (Crlf3), mRNA [NM_018776]	-1.56	9.54e-06	8.44e-04
Fam181b	*Mus musculus* family with sequence similarity 181, member B (Fam181b),<br>mRNA [NM_021427]	3.79	9.89e-06	8.70e-04
Arhgef10l	*Mus musculus* Rho guanine nucleotide exchange factor (GEF) 10-like<br>(Arhgef10l), transcript variant 1, mRNA[NM_172415]	1.95	1.01e-05	8.82e-04
Mtvr2	*Mus musculus* mammary tumor virus receptor 2 (Mtvr2), transcript variant 1,<br>mRNA [NM_181452]	1.23	1.01e-05	8.82e-04
Tiparp	*Mus musculus* TCDD-inducible<br>poly(ADP-ribose) polymerase (Tiparp),mRNA [NM_178892]	1.21	1.02e-05	8.87e-04
Dock5	*Mus musculus* dedicator of cytokinesis 5<br>(Dock5), mRNA [NM_177780]	-1.89	1.04e-05	9.01e-04
Pitpnm1	*Mus musculus* phosphatidylinositol transfer protein, membrane-associated 1(Pitpnm1), transcript variant 2, mRNA [NM_001136078]	1.47	1.05e-05	9.07e-04
Gm8225	PREDICTED: *Mus musculus* predictedgene, EG666668 (EG666668), mRNA<br>[XM_985281]	1.05	1.05e-05	9.08e-04
Gsto1	*Mus musculus* glutathione S-transferaseomega 1 (Gsto1), mRNA [NM_010362]	2.08	1.06e-05	9.10e-04
Gm8842	PREDICTED: *Mus musculus* predictedgene, EG667847, transcript variant 2<br>(EG667847), mRNA [XM_001003664]	0.75	1.06e-05	9.10e-04
Dhrs3	*Mus musculus* dehydrogenase/reductase (SDR family) member 3 (Dhrs3), mRNA<br>[NM_011303]	1.41	1.06e-05	9.10e-04
Lrrc1	*Mus musculus* leucine rich repeat<br>containing 1 (Lrrc1), transcript variant 2,mRNA [NM_172528]	-1.05	1.06e-05	9.10e-04
Degs1	*Mus musculus* degenerative spermatocytehomolog 1 (Drosophila) (Degs1), mRNA<br>[NM_007853]	-0.96	1.07e-05	9.10e-04
2810408A 11Rik	*Mus musculus* RIKEN cDNA<br>2810408A11 gene (2810408A11Rik),mRNA [NM_027419]	1.35	1.07e-05	9.10e-04
Pdlim4	*Mus musculus* PDZ and LIM domain 4<br>(Pdlim4), mRNA [NM_019417]	4.24	1.08e-05	9.12e-04
Trio	*Mus musculus* triple functional domain<br>(PTPRF interacting) (Trio), mRNA[NM_001081302]	2.17	1.09e-05	9.21e-04
Eif3c	*Mus musculus* eukaryotic translationinitiation factor 3, subunit C (Eif3c),<br>mRNA [NM_146200]	1.05	1.11e-05	9.31e-04
Lrp1	*Mus musculus* low density lipoprotein receptor-related protein 1 (Lrp1), mRNA<br>[NM_008512]	1.57	1.14e-05	9.35e-04
LOC1000039646	PREDICTED: *Mus musculus* similar topolyprotein (LOC100039646), mRNA<br>[XM_001472835]	-1.89	1.12e-05	9.35e-04
1700047M11Rik	*Mus musculus* RIKEN cDNA 1700047M11 gene (1700047M11Rik),<br>non-coding RNA [NR_015458]	-1.36	1.12e-05	9.35e-04
Nup62	*Mus musculus* nucleoporin 62 (Nup62),<br>mRNA [NM_053074]	1.35	1.12e-05	9.35e-04
Ccdc86	*Mus musculus* coiled-coil domaincontaining 86 (Ccdc86), mRNA<br>[NM_023731]	1.34	1.13e-05	9.35e-04
Fbxo25	*Mus musculus* F-box protein 25<br>(Fbxo25), mRNA [NM_025785]	-1.13	1.13e-05	9.35e-04
Idi1	*Mus musculus* isopentenyl-diphosphatedelta isomerase (Idi1), mRNA	-1.66	1.14e-05	9.35e-04

	[NM_145360]			
Herc4	*Mus musculus* hect domain and RLD 4 (Herc4), mRNA [NM_026101]	-1.45	1.14e-05	9.35e-04
Sorl1	*Mus musculus* sortilin-related receptor, LDLR class A repeats-containing (Sorl1),mRNA [NM_011436]	-1.54	1.15e-05	9.35e-04
Dtx4	*Mus musculus* deltex 4 homolog(Drosophila) (Dtx4), mRNA [NM_172442]	1.16	1.15e-05	9.35e-04
Prkcz	*Mus musculus* protein kinase C, zeta (Prkcz), transcript variant 1, mRNA[NM_008860]	-1.26	1.15e-05	9.39e-04
Bex2	*Mus musculus* brain expressed X-linked 2 (Bex2), mRNA [NM_009749]	1.76	1.18e-05	9.56e-04
Pdrg1	*Mus musculus* p53 and DNA damage regulated 1 (Pdrg1), mRNA[NM_178939]	0.90	1.18e-05	9.56e-04
Pex1	*Mus musculus* peroxisomal biogenesis factor 1 (Pex1), mRNA [NM_027777]	-1.24	1.19e-05	9.62e-04
Elovl5	*Mus musculus* ELOVL family member 5,elongation of long chain fatty acids (yeast) (Elovl5), mRNA [NM_134255]	-0.98	1.20e-05	9.69e-04
Klf10	*Mus musculus* Kruppel-like factor 10 (Klf10), mRNA [NM_013692]	1.57	1.23e-05	9.86e-04
Synm	*Mus musculus* synemin, intermediatefilament protein (Synm), transcript variant 1, mRNA [NM_201639]	-1.42	1.23e-05	9.86e-04
Nod1	*Mus musculus* nucleotide-binding oligomerization domain containing 1 (Nod1), mRNA [NM_172729]	-1.35	1.23e-05	9.86e-04
Tsc22d1	*Mus musculus* TSC22 domain family, member 1 (Tsc22d1), transcript variant 2, mRNA [NM_009366]	1.51	1.26e-05	9.96e-04
Rps15	*Mus musculus* ribosomal protein S15 (Rps15), mRNA [NM_009091]	0.91	1.26e-05	9.96e-04
Pcolce	*Mus musculus* procollagen C-endopeptidase enhancer protein (Pcolce),mRNA [NM_008788]	1.33	1.26e-05	9.96e-04
Phlda1	*Mus musculus* pleckstrin homology-like domain, family A, member 1 (Phlda1),mRNA [NM_009344]	1.12	1.26e-05	9.96e-04

Table S6. Common differentially expressed genes identified in the 4-week demyelinated *corpus callosum* and in the oligodendrocyte progenitor cell analysis.

**Symbol**	**Gene name**
Gdf15	growth differentiation factor 15
Pigz	phosphatidylinositol glycan anchor biosynthesis, class Z
Trib3	tribbles pseudokinase 3
Ninj2	ninjurin 2
Ccng1	cyclin G1
Slc34a3	solute carrier family 34 (sodium phosphate), member 3
Atf5	activating transcription factor 5
Xrcc3	X-ray repair complementing defective repair in Chinese hamster cells 3
Ddit3	DNA-damage inducible transcript 3
Moxd1	monooxygenase, DBH-like 1
Smtnl2	smoothelin-like 2
Ppp1r14a	protein phosphatase 1, regulatory inhibitor subunit 14A
Sesn2	sestrin 2
Cdkn1a	cyclin-dependent kinase inhibitor 1A (P21)
Tmem125	transmembrane protein 125
Tmprss5	transmembrane protease, serine 5 (spinesin)
Slc7a5	solute carrier family 7 (cationic amino acid transporter, y+ system), member 5
Klf4	Kruppel-like factor 4 (gut)
Eif4ebp1	eukaryotic translation initiation factor 4E binding protein 1
Slc3a2	solute carrier family 3 (activators of dibasic and neutral amino acid transport), member 2
Arap2	ArfGAP with RhoGAP domain, ankyrin repeat and PH domain 2
Gzmm	granzyme M (lymphocyte met-ase 1)
Sgk2	serum/glucocorticoid regulated kinase 2

Bbc3	BCL2 binding component 3
Nes	nestin
Gas5	growth arrest specific 5
Fdps	farnesyl diphosphate synthetase
Prima1	proline rich membrane anchor 1
Serpinb1a	serine (or cysteine) peptidase inhibitor, clade B, member 1a
Ldlr	low density lipoprotein receptor
Gjc2	gap junction protein, gamma 2
Ephx1	epoxide hydrolase 1, microsomal
Ephx1	epoxide hydrolase 1, microsomal
B230206H07Rik	RIKEN cDNA B230206H07 gene
Hmgcs1	3-hydroxy-3-methylglutaryl-Coenzyme A synthase 1
Galnt6	polypeptide N-acetylgalactosaminyltransferase 6
Galnt6	polypeptide N-acetylgalactosaminyltransferase 6
Nupr1	nuclear protein transcription regulator 1
Adamts1	a disintegrin-like and metallopeptidase (reprolysin type) with thrombospondin type 1 motif, 1
Pcyt2	phosphate cytidylyltransferase 2, ethanolamine
Gadd45b	growth arrest and DNA-damage-inducible 45 beta
Nacad	NAC alpha domain containing
Klk6	kallikrein related-peptidase 6
Tmeff1	transmembrane protein with EGF-like and two follistatin-like domains 1
Mog	myelin oligodendrocyte glycoprotein
Aen	apoptosis enhancing nuclease
Nmral1	NmrA-like family domain containing 1
Rftn1	raftlin lipid raft linker 1
Eprs	glutamyl-prolyl-tRNA synthetase
Nkain1	Na+/K+ transporting ATPase interacting 1
Fzd1	frizzled class receptor 1
Rps27l	ribosomal protein S27-like
Rhog	ras homolog family member G
Arrdc4	arrestin domain containing 4
Opalin	oligodendrocytic myelin paranodal and inner loop protein
Anln	anillin, actin binding protein
Sytl2	synaptotagmin-like 2
Ccl2	chemokine (C-C motif) ligand 2
Gadd45a	growth arrest and DNA-damage-inducible 45 alpha
Prr18	proline rich 18
Synj2	synaptojanin 2
Ifrd1	interferon-related developmental regulator 1
Adssl1	adenylosuccinate synthetase like 1
Kctd15	potassium channel tetramerisation domain containing 15
Ppp1r15a	protein phosphatase 1, regulatory subunit 15A
Gal3st1	galactose-3-O-sulfotransferase 1
Maff	v-maf musculoaponeurotic fibrosarcoma oncogene family, protein F (avian)
Cntn2	contactin 2
Nipal4	NIPA-like domain containing 4
Cdk18	cyclin-dependent kinase 18
Mgp	matrix Gla protein
Ptprd	protein tyrosine phosphatase, receptor type, D
Lgi3	leucine-rich repeat LGI family, member 3
Tor3a	torsin family 3, member A
Mcam	melanoma cell adhesion molecule
Fah	fumarylacetoacetate hydrolase
Rgs3	regulator of G-protein signaling 3
Nfil3	nuclear factor, interleukin 3, regulated
Mbp	myelin basic protein
Dhcr7	7-dehydrocholesterol reductase
Ano4	anoctamin 4
Cyb5r2	cytochrome b5 reductase 2
Plekhh1	pleckstrin homology domain containing, family H (with MyTH4 domain) member 1
Ttll7	tubulin tyrosine ligase-like family, member 7
Runx1	runt related transcription factor 1
Odc1	ornithine decarboxylase, structural 1
Adamtsl4	ADAMTS-like 4

Gamt	guanidinoacetate methyltransferase
Rasgrp3	RAS, guanyl releasing protein 3
Cebpg	CCAAT/enhancer binding protein (C/EBP), gamma
1110038B12Rik	RIKEN cDNA 1110038B12 gene
Cebpb	CCAAT/enhancer binding protein (C/EBP), beta
Abca2	ATP-binding cassette, sub-family A (ABC1), member 2
Omg	oligodendrocyte myelin glycoprotein
Tppp	tubulin polymerization promoting protein
Mast4	microtubule associated serine/threonine kinase family member 4
Rasl12	RAS-like, family 12

Table S7. Differentially expressed genes in human samples from multiple sclerosis plaques compared to control white matter from brain and spinal cord.

Symbol	Gene name	Log2 fold change	p-value	Adjusted p-value
SIRT2	sirtuin 2	-1.02	3.04e-07	7.07e-03
NLGN3	neuroligin 3	-0.98	5.34e-07	7.07e-03
LDB3	LIM domain binding 3	-0.71	1.01e-06	7.07e-03
HAPLN2	hyaluronan and proteoglycan link protein 2	-1.43	1.02e-06	7.07e-03
PACS2	phosphofurin acidic cluster sorting protein 2	-1.23	1.11e-06	7.07e-03
CDYL2	chromodomain Y like 2	1.45	1.68e-06	7.07e-03
ARHGAP23	Rho GTPase activating protein 23	-1.08	2.41e-06	7.07e-03
RAPGEF5	Rap guanine nucleotide exchange factor 5	-1.13	2.60e-06	7.07e-03
PPP3CA	protein phosphatase 3 catalytic subunit alpha	1.98	2.72e-06	7.07e-03
RECQL	RecQ like helicase	0.97	2.84e-06	7.07e-03
BACE1	beta-secretase 1	-1.22	3.00e-06	7.07e-03
TTYH2	tweety family member 2	-1.09	3.50e-06	7.07e-03
SHTN1	shootin 1	-0.69	3.58e-06	7.07e-03
HS2ST1	heparan sulfate 2-O-sulfotransferase 1	1.77	3.59e-06	7.07e-03
RFFL	ring finger and FYVE like domain containing E3 ubiquitin protein ligase	-1.11	3.81e-06	7.07e-03
PPP3CA	protein phosphatase 3 catalytic subunit alpha	2.70	3.86e-06	7.07e-03
BACE1	beta-secretase 1	-1.17	4.01e-06	7.07e-03
LDLRAP1	low density lipoprotein receptor adaptor protein 1	-1.43	4.08e-06	7.07e-03
BVES	blood vessel epicardial substance	-1.19	4.20e-06	7.07e-03
PAQR4	progestin and adipoQ receptor family member 4	-1.17	4.32e-06	7.07e-03
WIPF1	WAS/WASL interacting protein family member 1	-1.00	4.62e-06	7.19e-03
MTSS1	MTSS I-BAR domain containing 1	-0.89	6.88e-06	1.02e-02
SORT1	sortilin 1	-0.62	7.95e-06	1.05e-02
GJC2	gap junction protein gamma 2	-0.95	8.04e-06	1.05e-02
PLOD3	procollagen-lysine,2-oxoglutarate 5-dioxygenase 3	-1.01	1.08e-05	1.27e-02
FA2H	fatty acid 2-hydroxylase	-1.69	1.08e-05	1.27e-02
LHPP	phospholysine	-1.34	1.08e-05	1.27e-02

	phosphohistidine inorganic pyrophosphate phosphatase			
PKP4	plakophilin 4	-0.99	1.33e-05	1.43e-02
PRIMA1	proline rich membrane anchor 1	-1.71	1.35e-05	1.43e-02
ELAVL3	ELAV like RNA binding protein 3	-1.10	1.36e-05	1.43e-02
WBP4	WW domain binding protein 4	1.86	1.51e-05	1.47e-02
BRCC3	BRCA1/BRCA2-containing complex subunit 3	1.19	1.53e-05	1.47e-02
PNMA2	PNMA family member 2	1.68	1.56e-05	1.47e-02
AHNAK2	AHNAK nucleoprotein 2	1.26	1.57e-05	1.47e-02
SLC25A13	solute carrier family 25 member 13	-1.42	1.87e-05	1.67e-02
PLEKHB1	pleckstrin homology domain containing B1	-0.78	1.91e-05	1.67e-02
RILPL1	Rab interacting lysosomal protein like 1	-0.84	1.94e-05	1.67e-02
GLTP	glycolipid transfer protein	-1.38	2.14e-05	1.79e-02
TP53INP2	tumor protein p53 inducible nuclear protein 2	-1.30	2.31e-05	1.89e-02
PPM1L	protein phosphatase, Mg2+/Mn2+ dependent 1L	1.27	2.62e-05	2.07e-02
MBP	myelin basic protein	-1.26	2.77e-05	2.07e-02
ARHGEF3	Rho guanine nucleotide exchange factor 3	1.56	2.77e-05	2.07e-02
CERS6	ceramide synthase 6	1.27	2.79e-05	2.07e-02
CANX	calnexin	1.45	2.92e-05	2.12e-02
LIPE	lipase E, hormone sensitive type	-1.13	3.00e-05	2.13e-02
CDK18	cyclin dependent kinase 18	-1.29	3.09e-05	2.15e-02
DPYSL5	dihydropyrimidinase like 5	-1.08	3.20e-05	2.18e-02
BOK	BCL2 family apoptosis regulator BOK	-1.49	3.29e-05	2.19e-02
ATP6V1C1	ATPase H+ transporting V1 subunit C1	1.08	3.54e-05	2.29e-02
UNC5C	unc-5 netrin receptor C	-1.24	3.71e-05	2.29e-02
KIF1C	kinesin family member 1C	-0.91	3.71e-05	2.29e-02
GNG7	G protein subunit gamma 7	-0.96	3.74e-05	2.29e-02
SNX22	sorting nexin 22	-0.95	3.83e-05	2.29e-02
ARHGAP26	Rho GTPase activating protein 26	1.75	3.85e-05	2.29e-02
AFMID	arylformamidase	-0.68	3.96e-05	2.31e-02
FAM102A	family with sequence similarity 102 member A	-0.96	4.05e-05	2.31e-02
PIP4K2A	phosphatidylinositol-5-phosphate 4-kinase type 2 alpha	-0.95	4.10e-05	2.31e-02
S1PR5	sphingosine-1-phosphate receptor 5	-1.78	4.27e-05	2.35e-02
FRMD6	FERM domain containing 6	1.65	4.35e-05	2.35e-02
SOX10	SRY-box transcription factor 10	-1.17	4.46e-05	2.35e-02
ZNF770	zinc finger protein 770	1.45	4.46e-05	2.35e-02
FCSK	fucose kinase	-0.87	4.53e-05	2.35e-02
UBIAD1	UbiA prenyltransferase domain containing 1	-0.58	4.74e-05	2.41e-02
MAPK8IP1	mitogen-activated protein kinase 8 interacting protein 1	-1.27	4.92e-05	2.41e-02
SSBP2	single stranded DNA binding protein 2	1.17	4.95e-05	2.41e-02
LPAR1	lysophosphatidic acid receptor 1	-1.40	5.16e-05	2.41e-02

LCN2	lipocalin 2	-0.45	5.20e-05	2.41e-02
JPT2	Jupiter microtubule associated homolog 2	-1.30	5.24e-05	2.41e-02
PHF20L1	PHD finger protein 20 like 1	2.10	5.28e-05	2.41e-02
PLEKHH1	pleckstrin homology, MyTH4 and FERM domain containing H1	-1.61	5.52e-05	2.41e-02
LMF1	lipase maturation factor 1	-1.07	5.54e-05	2.41e-02
DBNDD2	dysbindin domain containing 2	-1.17	5.59e-05	2.41e-02
TLE6	TLE family member 6, subcortical maternal complex member	-0.37	5.64e-05	2.41e-02
SH3PXD2A	SH3 and PX domains 2A	-0.63	5.65e-05	2.41e-02
MARCKSL 1	MARCKS like 1	-0.90	5.74e-05	2.41e-02
TMEM63A	transmembrane protein 63A	-0.94	5.90e-05	2.44e-02
EFHD1	EF-hand domain family member D1	-0.95	6.01e-05	2.45e-02
APC	APC regulator of WNT signaling pathway	1.61	6.22e-05	2.51e-02
HAGLR	HOXD antisense growth-associated long non-coding RNA	-1.64	6.32e-05	2.52e-02
FAM234A	family with sequence similarity 234 member A	-0.95	6.47e-05	2.52e-02
SF3B1	splicing factor 3b subunit 1	1.20	6.47e-05	2.52e-02
OLIG1	oligodendrocyte transcription factor 1	-1.37	6.67e-05	2.54e-02
XK	X-linked Kx blood group	1.74	6.81e-05	2.54e-02
ARHGEF37	Rho guanine nucleotide exchange factor 37	-1.28	6.86e-05	2.54e-02
AIF1L	allograft inflammatory factor 1 like	-1.45	6.89e-05	2.54e-02
NACAD	NAC alpha domain containing	-1.02	6.93e-05	2.54e-02
PAIP2B	poly(A) binding protein interacting protein 2B	-1.49	7.15e-05	2.56e-02
CD22	CD22 molecule	-0.91	7.19e-05	2.56e-02
RASSF2	Ras association domain family member 2	-1.47	7.58e-05	2.62e-02
MAP4	microtubule associated protein 4	-0.91	7.61e-05	2.62e-02
TMEM150C	transmembrane protein 150C	1.57	7.77e-05	2.65e-02
VWA1	von Willebrand factor A domain containing 1	-0.79	7.88e-05	2.65e-02
APLP1	amyloid beta precursor like protein 1	-1.04	8.27e-05	2.70e-02
PRKCSH	protein kinase C substrate 80K-H	-0.98	8.35e-05	2.70e-02
REEP3	receptor accessory protein 3	-1.12	8.92e-05	2.83e-02
MBNL2	muscleblind like splicing regulator 2	-0.86	9.05e-05	2.85e-02
CEP85	centrosomal protein 85	-0.55	9.29e-05	2.86e-02
SLC6A19	solute carrier family 6 member 19	-0.51	9.32e-05	2.86e-02
CDKN1C	cyclin dependent kinase inhibitor 1C	-1.16	9.37e-05	2.86e-02
FCHO1	FCH and mu domain containing endocytic adaptor 1	-0.57	9.53e-05	2.87e-02
SYNJ2	synaptojanin 2	-1.48	9.64e-05	2.87e-02

SEPTIN6	septin 6	1.05	9.69e-05	2.87e-02
LLGL1	LLGL scribble cell polarity complex component 1	-0.86	9.76e-05	2.87e-02
LRRC8D	leucine rich repeat containing 8 VRAC subunit D	-1.14	9.91e-05	2.87e-02
ATP11A	ATPase phospholipid transporting 11A	-0.54	9.94e-05	2.87e-02
CADPS2	calcium dependent secretion activator 2	1.85	1.05e-04	3.00e-02
LZTS2	leucine zipper tumor suppressor 2	-0.62	1.06e-04	3.00e-02
CAPRIN2	caprin family member 2	1.19	1.07e-04	3.01e-02
MTPN	myotrophin	0.65	1.08e-04	3.01e-02
TJAP1	tight junction associated protein 1	-0.94	1.09e-04	3.01e-02
ST3GAL4	ST3 beta-galactoside alpha-2,3-sialyltransferase 4	-0.53	1.14e-04	3.09e-02
SPARCL1	SPARC like 1	0.97	1.16e-04	3.09e-02
TMEM125	transmembrane protein 125	-2.01	1.16e-04	3.09e-02
PTPRH	protein tyrosine phosphatase receptor type H	-0.93	1.18e-04	3.09e-02
KIF6	kinesin family member 6	-0.80	1.19e-04	3.09e-02
GAB2	GRB2 associated binding protein 2	-0.82	1.19e-04	3.09e-02
SMAD5	SMAD family member 5	1.84	1.20e-04	3.09e-02
SEMA4D	semaphorin 4D	-1.56	1.20e-04	3.09e-02
ZNF148	zinc finger protein 148	1.35	1.22e-04	3.09e-02
PIAS1	protein inhibitor of activated STAT 1	0.99	1.23e-04	3.09e-02
SLC44A1	solute carrier family 44 member 1	-0.89	1.30e-04	3.19e-02
MYRF	myelin regulatory factor	-1.27	1.31e-04	3.19e-02
SP3	Sp3 transcription factor	1.75	1.32e-04	3.20e-02
SEMA6A	semaphorin 6A	-0.81	1.36e-04	3.23e-02
SLC48A1	solute carrier family 48 member 1	-0.80	1.38e-04	3.23e-02
TRIM62	tripartite motif containing 62	-0.57	1.38e-04	3.23e-02
PAQR6	progestin and adipoQ receptor family member 6	-0.77	1.39e-04	3.23e-02
GABRG1	gamma-aminobutyric acid type A receptor subunit gamma1	1.91	1.39e-04	3.23e-02
SEPTIN8	septin 8	-1.23	1.42e-04	3.24e-02
NBN	nibrin	1.09	1.43e-04	3.25e-02
CPD	carboxypeptidase D	-1.11	1.45e-04	3.27e-02
DHCR24	24-dehydrocholesterol reductase	-1.06	1.47e-04	3.28e-02
PGPEP1	pyroglutamyl-peptidase I	-0.74	1.47e-04	3.28e-02
AGBL5	ATP/GTP binding protein like 5	1.04	1.50e-04	3.31e-02
RBBP9	RB binding protein 9, serine hydrolase	-1.10	1.51e-04	3.31e-02
NKAIN2	sodium/potassium transporting ATPase interacting 2	-1.18	1.52e-04	3.31e-02
PLEKHA5	pleckstrin homology domain containing A5	1.95	1.53e-04	3.31e-02
OBI1	ORC ubiquitin ligase 1	1.17	1.54e-04	3.31e-02
DNTTIP2	deoxynucleotidyltransferase terminal interacting protein 2	1.11	1.55e-04	3.31e-02
PLCL2	phospholipase C like 2	1.67	1.58e-04	3.35e-02
LRRC6	leucine rich repeat containing 6	1.10	1.61e-04	3.36e-02

PTPRO	protein tyrosine phosphatase receptor type O	1.38	1.62e-04	3.36e-02
NCAM1	neural cell adhesion molecule 1	-1.33	1.62e-04	3.36e-02
SLC5A11	solute carrier family 5 member 11	-1.58	1.64e-04	3.39e-02
ZHX1	zinc fingers and homeoboxes 1	1.10	1.65e-04	3.39e-02
JAM3	junctional adhesion molecule 3	-0.76	1.67e-04	3.41e-02
RBMS3	RNA binding motif single stranded interacting protein 3	3.41	1.69e-04	3.43e-02
SLC6A9	solute carrier family 6 member 9	-1.01	1.70e-04	3.44e-02
ZNF488	zinc finger protein 488	-1.11	1.71e-04	3.44e-02
NKX2-2	NK2 homeobox 2	-1.79	1.73e-04	3.45e-02
PXK	PX domain containing serine/threonine kinase like	-1.07	1.75e-04	3.46e-02
INTS6	integrator complex subunit 6	1.22	1.76e-04	3.46e-02
TMC6	transmembrane channel like 6	-1.23	1.79e-04	3.50e-02
PBX1	PBX homeobox 1	1.07	1.83e-04	3.53e-02
SEC23A	Sec23 homolog A, COPII coat complex component	1.29	1.84e-04	3.54e-02
DAAM1	dishevelled associated activator of morphogenesis 1	2.16	1.86e-04	3.56e-02
POLR2J4	RNA polymerase II subunit J4, pseudogene	-0.91	1.88e-04	3.58e-02
C10orf90	chromosome 10 open reading frame 90	-1.37	1.92e-04	3.62e-02
GPR37	G protein-coupled receptor 37	-1.13	1.93e-04	3.62e-02
RASAL1	RAS protein activator like 1	-0.32	1.97e-04	3.64e-02
TRIM69	tripartite motif containing 69	0.76	1.97e-04	3.64e-02
PPM1A	protein phosphatase, Mg2+/Mn2+ dependent 1A	1.31	1.97e-04	3.64e-02
RCN1	reticulocalbin 1	1.15	1.99e-04	3.64e-02
TBC1D24	TBC1 domain family member 24	0.84	2.01e-04	3.64e-02
GM2A	GM2 ganglioside activator	-0.92	2.01e-04	3.64e-02
FGD6	FYVE, RhoGEF and PH domain containing 6	0.52	2.03e-04	3.64e-02
CHMP3	charged multivesicular body protein 3	1.37	2.04e-04	3.64e-02
SETDB2	SET domain bifurcated histone lysine methyltransferase 2	0.80	2.08e-04	3.69e-02
MOBP	myelin associated oligodendrocyte basic protein	-1.43	2.10e-04	3.71e-02
UGT8	UDP glycosyltransferase 8	-1.61	2.12e-04	3.72e-02
PEX16	peroxisomal biogenesis factor 16	-0.61	2.13e-04	3.72e-02
CNTN2	contactin 2	-1.25	2.24e-04	3.90e-02
NHS	NHS actin remodeling regulator	1.62	2.27e-04	3.91e-02
TRAK2	trafficking kinesin protein 2	-0.94	2.30e-04	3.91e-02
FAM124A	family with sequence similarity 124 member A	-1.02	2.30e-04	3.91e-02
KANK1	KN motif and ankyrin repeat domains 1	-0.57	2.32e-04	3.91e-02
ATP2B1	ATPase plasma membrane Ca2+ transporting 1	1.68	2.33e-04	3.91e-02
CDC42EP2	CDC42 effector protein 2	-0.75	2.35e-04	3.91e-02
SKIL	SKI like proto-oncogene	1.64	2.35e-04	3.91e-02
PPIG	peptidylprolyl isomerase G	1.50	2.40e-04	3.98e-02
ABI2	abl interactor 2	0.97	2.45e-04	4.01e-02

SCD	stearoyl-CoA desaturase	-1.13	2.47e-04	4.01e-02
GPATCH2	G-patch domain containing 2	1.42	2.48e-04	4.01e-02
PPP2R5C	protein phosphatase 2 regulatory subunit B'gamma	1.13	2.48e-04	4.01e-02
SEMA4C	semaphorin 4C	-0.60	2.50e-04	4.02e-02
PLPP2	phospholipid phosphatase 2	-1.30	2.51e-04	4.02e-02
FOXC2	forkhead box C2	-0.54	2.54e-04	4.03e-02
TPRN	taperin	-0.78	2.55e-04	4.03e-02
SHROOM3	shroom family member 3	0.83	2.57e-04	4.03e-02
FGD5-AS1	FGD5 antisense RNA 1	-0.49	2.59e-04	4.03e-02
RPS6KA2	ribosomal protein S6 kinase A2	-0.68	2.61e-04	4.03e-02
ANKRD50	ankyrin repeat domain 50	1.53	2.61e-04	4.03e-02
TMEM161A	transmembrane protein 161A	-0.62	2.64e-04	4.03e-02
KAZN	kazrin, periplakin interacting protein	-0.59	2.64e-04	4.03e-02
SLC7A2	solute carrier family 7 member 2	1.59	2.65e-04	4.03e-02
TUBB4A	tubulin beta 4A class IVa	-1.03	2.68e-04	4.04e-02
MFHAS1	malignant fibrous histiocytoma amplified sequence 1	0.94	2.70e-04	4.04e-02
CHADL	chondroadherin like	-1.26	2.70e-04	4.04e-02
CLMN	calmin	-0.79	2.72e-04	4.07e-02
PCBP4	poly(rC) binding protein 4	-0.90	2.74e-04	4.08e-02
MAVS	mitochondrial antiviral signaling protein	-0.53	2.77e-04	4.09e-02
PPP1CB	protein phosphatase 1 catalytic subunit beta	0.90	2.78e-04	4.09e-02
BCAS1	brain enriched myelin associated protein 1	-1.05	2.80e-04	4.10e-02
GPIHBP1	glycosylphosphatidylinositol anchored high density lipoprotein binding protein 1	-1.79	2.83e-04	4.11e-02
RAB3GAP1	RAB3 GTPase activating protein catalytic subunit 1	1.03	2.86e-04	4.11e-02
SMAD1	SMAD family member 1	2.13	2.86e-04	4.11e-02
FEZ1	fasciculation and elongation protein zeta 1	-0.84	2.87e-04	4.11e-02
KITLG	KIT ligand	2.01	2.94e-04	4.17e-02
ASPA	aspartoacylase	-0.92	2.94e-04	4.17e-02
TNS1	tensin 1	-0.98	2.97e-04	4.18e-02
GUCY1B1	guanylate cyclase 1 soluble subunit beta 1	0.90	3.04e-04	4.27e-02
SDHAF1	succinate dehydrogenase complex assembly factor 1	-0.79	3.09e-04	4.30e-02
PIEZO2	piezo type mechanosensitive ion channel component 2	-1.54	3.10e-04	4.30e-02
NDUFS7	NADH:ubiquinone oxidoreductase core subunit S7	-0.82	3.10e-04	4.30e-02
SEC63	SEC63 homolog, protein translocation regulator	1.10	3.15e-04	4.34e-02
DNAH17	dynein axonemal heavy chain 17	-1.83	3.16e-04	4.34e-02
SNTB2	syntrophin beta 2	1.51	3.28e-04	4.47e-02
CBX5	chromobox 5	0.94	3.30e-04	4.47e-02
ANKLE2	ankyrin repeat and LEM domain containing 2	-0.79	3.31e-04	4.47e-02
GJB1	gap junction protein beta 1	-1.14	3.32e-04	4.47e-02
JUP	junction plakoglobin	-0.70	3.34e-04	4.47e-02
SNX13	sorting nexin 13	1.87	3.38e-04	4.50e-02

NCEH1	neutral cholesterol ester hydrolase 1	2.43	3.41e-04	4.53e-02
NENF	neudesin neurotrophic factor	-0.85	3.43e-04	4.54e-02
CCDC14	coiled-coil domain containing 14	1.53	3.46e-04	4.56e-02
TTLL11	tubulin tyrosine ligase like 11	-0.92	3.48e-04	4.56e-02
EPHA5	EPH receptor A5	1.61	3.49e-04	4.56e-02
GNAL	G protein subunit alpha L	1.53	3.50e-04	4.56e-02
BBS7	Bardet-Biedl syndrome 7	1.56	3.59e-04	4.64e-02
H2AJ	H2A.J histone	-0.98	3.63e-04	4.67e-02
ARHGEF2	Rho/Rac guanine nucleotide exchange factor 2	-0.89	3.64e-04	4.67e-02
NDUFA4L2	NDUFA4 mitochondrial complex associated like 2	-0.62	3.67e-04	4.68e-02
QKI	QKI, KH domain containing RNA binding	-1.65	3.71e-04	4.70e-02
GTF2IRD2B	GTF2I repeat domain containing 2B	-0.66	3.72e-04	4.70e-02
AKAP8L	A-kinase anchoring protein 8 like	-0.36	3.75e-04	4.70e-02
WNK1	WNK lysine deficient protein kinase 1	-0.75	3.75e-04	4.70e-02
SSH3	slingshot protein phosphatase 3	-0.48	3.78e-04	4.70e-02
FRY	FRY microtubule binding protein	1.40	3.78e-04	4.70e-02
GAL3ST1	galactose-3-O-sulfotransferase 1	-0.79	3.84e-04	4.75e-02
LINC01315	long intergenic non-protein coding RNA 1315	-1.05	3.85e-04	4.75e-02
EFR3A	EFR3 homolog A	1.59	3.87e-04	4.75e-02
NR2F1-AS1	NR2F1 antisense RNA 1	1.06	3.88e-04	4.75e-02
RASGRP3	RAS guanyl releasing protein 3	-1.52	3.95e-04	4.82e-02
ZNF391	zinc finger protein 391	1.46	4.02e-04	4.88e-02
CLDN11	claudin 11	-0.75	4.04e-04	4.88e-02
ANXA11	annexin A11	0.64	4.07e-04	4.88e-02
PCGF5	polycomb group ring finger 5	1.91	4.08e-04	4.88e-02

YIF1B	Yip1 interacting factor homolog B, membrane trafficking protein	-0.94	4.15e-04	4.95e-02
MVB12B	multivesicular body subunit 12B	-0.94	4.17e-04	4.95e-02
NR3C2	nuclear receptor subfamily 3 group C member 2	1.16	4.18e-04	4.95e-02
TRIM41	tripartite motif containing 41	-0.81	4.24e-04	4.98e-02
CERS2	ceramide synthase 2	-1.01	4.24e-04	4.98e-02
BCL2L11	BCL2 like 11	-0.48	4.26e-04	4.99e-02
SNRPC	small nuclear ribonucleoprotein polypeptide C	-0.73	4.28e-04	4.99e-02

Table S8. Common differentially expressed genes identified in the human multiple sclerosis and in the oligodendrocyte progenitor cell analyses.

**Symbol**	**Gene name**
S1pr5	*Mus musculus* sphingosine-1-phosphate receptor 5 (S1pr5), mRNA [NM_053190]
Efhd1	*Mus musculus* EF hand domain containing 1 (Efhd1), mRNA [NM_028889]
Synj2	*Mus musculus* synaptojanin 2 (Synj2), transcript variant 3, mRNA [NM_011523]
Slc5a11	*Mus musculus* solute carrier family 5 (sodium/glucose cotransporter), member 11 (Slc5a11), mRNA [NM_146198]
Sort1	*Mus musculus* sortilin 1 (Sort1), mRNA [NM_019972]
Aspa	*Mus musculus* aspartoacylase (Aspa), mRNA [NM_023113]
Jam3	*Mus musculus* junction adhesion molecule 3 (Jam3), mRNA [NM_023277]
Daam1	*Mus musculus* dishevelled associated activator of morphogenesis 1 (Daam1), transcript variant 1, mRNA [NM_026102]
Ptpro	*Mus musculus* protein tyrosine phosphatase, receptor type, O (Ptpro), transcript variant 1, mRNA [NM_011216]
Cdkn1c	*Mus musculus* cyclin-dependent kinase inhibitor 1C (P57) (Cdkn1c), transcript variant 2, mRNA [NM_009876]
Fa2h	*Mus musculus* fatty acid 2-hydroxylase (Fa2h), mRNA [NM_178086]
Slc25a13	*Mus musculus* solute carrier family 25 (mitochondrial carrier, adenine nucleotide translocator), member 13 (Slc25a13), nuclear gene encoding mitochondrial protein, mRNA [NM_015829]
Tle6	*Mus musculus* transducin-like enhancer of split 6, homolog of Drosophila E(spl) (Tle6), mRNA [NM_053254]
Clmn	*Mus musculus* calmin (Clmn), transcript variant 1, mRNA [NM_053155]
Gjc2	*Mus musculus* gap junction protein, gamma 2 (Gjc2), transcript variant 2, mRNA [NM_175452]
Rasal1	*Mus musculus* RAS protein activator like 1 (GAP1 like) (Rasal1), mRNA [NM_013832]
Pcgf5	*Mus musculus* polycomb group ring finger 5 (Pcgf5), mRNA [NM_029508]
Aif1l	*Mus musculus* allograft inflammatory factor 1-like (Aif1l), mRNA [NM_145144]
Rassf2	*Mus musculus* Ras association (RalGDS/AF-6) domain family member 2 (Rassf2), mRNA [NM_175445]
Cdk18	*Mus musculus* cyclin-dependent kinase 18 (Cdk18), mRNA [NM_008795]
Wnk1	*Mus musculus* WNK lysine deficient protein kinase 1 (Wnk1), transcript variant 1, mRNA [NM_198703]

Kank1	*Mus musculus* KN motif and ankyrin repeat domains 1 (Kank1), mRNA [NM_181404]
Elavl3	*Mus musculus* ELAV (embryonic lethal, abnormal vision, Drosophila)-like 3 (Hu antigen C) (Elavl3), mRNA [NM_010487]
Mobp	*Mus musculus* myelin-associated oligodendrocytic basic protein (Mobp), transcript variant 3, mRNA [NM_001039365]
Fez1	*Mus musculus* fasciculation and elongation protein zeta 1 (zygin I) (Fez1), mRNA [NM_183171]
Kif6	*Mus musculus* kinesin family member 6 (Kif6), mRNA [NM_177052]
Prima1	*Mus musculus* proline rich membrane anchor 1 (Prima1), mRNA [NM_133364]
Slc44a1	*Mus musculus* solute carrier family 44, member 1 (Slc44a1), transcript variant 1, mRNA [NM_133891]
Paqr4	*Mus musculus* progestin and adipoQ receptor family member IV (Paqr4), mRNA [NM_023824]
Epha5	*Mus musculus* Eph receptor A5 (Epha5), mRNA [NM_007937]
Paqr6	*Mus musculus* progestin and adipoQ receptor family member VI (Paqr6), mRNA [NM_198410]
Gal3st1	*Mus musculus* galactose-3-O-sulfotransferase 1 (Gal3st1), mRNA [NM_016922]
Rapgef5	*Mus musculus* Rap guanine nucleotide exchange factor (GEF) 5 (Rapgef5), mRNA [NM_175930]
Cdc42ep2	*Mus musculus* CDC42 effector protein (Rho GTPase binding) 2 (Cdc42ep2), mRNA [NM_026772]
Fam102a	*Mus musculus* family with sequence similarity 102, member A (Fam102a), mRNA [NM_153560]
Reep3	*Mus musculus* receptor accessory protein 3 (Reep3), mRNA [NM_178606]
Cpd	*Mus musculus* carboxypeptidase D (Cpd), mRNA [NM_007754]
Rasgrp3	*Mus musculus* RAS, guanyl releasing protein 3 (Rasgrp3), transcript variant 2, mRNA [NM_207246]
Tmem125	*Mus musculus* transmembrane protein 125 (Tmem125), mRNA [NM_172383]
Tmem63a	*Mus musculus* transmembrane protein 63a (Tmem63a), mRNA [NM_144794]
Lpar1	*Mus musculus* lysophosphatidic acid receptor 1 (Lpar1), transcript variant 2, mRNA [NM_172989]
Plekhh1	*Mus musculus* pleckstrin homology domain containing, family H (with MyTH4 domain) member 1 (Plekhh1), mRNA [NM_181073]
Tmem161a	*Mus musculus* transmembrane protein 161A (Tmem161a), mRNA [NM_145597]
Atp11a	*Mus musculus* ATPase, class VI, type 11A (Atp11a), mRNA [NM_015804]
Cntn2	*Mus musculus* contactin 2 (Cntn2), mRNA [NM_177129]
Jup	*Mus musculus* junction plakoglobin (Jup), mRNA [NM_010593]
Arhgef2	*Mus musculus* rho/rac guanine nucleotide exchange factor (GEF) 2 (Arhgef2), mRNA [NM_008487]
Tmc6	*Mus musculus* transmembrane channel-like gene family 6 (Tmc6), transcript variant 2, mRNA [NM_181321]
Chadl	*Mus musculus* chondroadherin-like (Chadl), mRNA [NM_001164320]
Lzts2	*Mus musculus* leucine zipper, putative tumor suppressor 2 (Lzts2), transcript variant 1, mRNA [NM_145503]
Cd22	*Mus musculus* CD22 antigen (Cd22), transcript variant 1, mRNA [NM_001043317]
Plekhb1	*Mus musculus* pleckstrin homology domain containing, family B (evectins) member 1 (Plekhb1), transcript variant 1, mRNA [NM_013746]
Slc6a9	*Mus musculus* solute carrier family 6 (neurotransmitter transporter, glycine), member 9 (Slc6a9), mRNA [NM_008135]
Ttyh2	*Mus musculus* tweety homolog 2 (Drosophila) (Ttyh2), mRNA [NM_053273]
Sdhaf1	*Mus musculus* succinate dehydrogenase complex assembly factor 1 (Sdhaf1), mRNA [NM_001033140]
Hapln2	*Mus musculus* hyaluronan and proteoglycan link protein 2 (Hapln2), mRNA [NM_022031]
Pip4k2a	*Mus musculus* phosphatidylinositol-5-phosphate 4-kinase, type II, alpha (Pip4k2a), mRNA [NM_008845]
St3gal4	*Mus musculus* ST3 beta-galactoside alpha-2,3-sialyltransferase 4 (St3gal4), mRNA [NM_009178]
Cadps2	*Mus musculus* Ca2+dependent activator protein for secretion 2 (Cadps2), mRNA [NM_153163]
Ppp2r5c	*Mus musculus* protein phosphatase 2, regulatory subunit B (B56), gamma isoform (Ppp2r5c), transcript variant 4, mRNA [NM_001135001]

Lmf1	*Mus musculus* lipase maturation factor 1 (Lmf1), mRNA [NM_029624]
Fgd6	*Mus musculus* FYVE, RhoGEF and PH domain containing 6 (Fgd6), mRNA [NM_053072]
Sirt2	*Mus musculus* sirtuin 2 (silent mating type information regulation 2, homolog) 2 (S. cerevisiae) (Sirt2), transcript variant 1, mRNA [NM_022432]
Mbp	*Mus musculus* myelin basic protein (Mbp), transcript variant 7, mRNA [NM_010777]
Yif1b	*Mus musculus* Yip1 interacting factor homolog B (S. cerevisiae) (Yif1b), transcript variant 1, mRNA [NM_029887]
Nacad	*Mus musculus* NAC alpha domain containing (Nacad), mRNA [NM_001081652]
Rbbp9	*Mus musculus* retinoblastoma binding protein 9 (Rbbp9), mRNA [NM_015754]
Nkain2	*Mus musculus* Na transporting ATPase interacting 2 (Nkain2), transcript variant 2, mRNA [NM_001025286]
Gltp	*Mus musculus* glycolipid transfer protein (Gltp), mRNA [NM_019821]
Ldb3	*Mus musculus* LIM domain binding 3 (Ldb3), transcript variant 1, mRNA [NM_011918]
Rilpl1	*Mus musculus* Rab interacting lysosomal protein-like 1 (Rilpl1), mRNA [NM_021430]
Mbnl2	*Mus musculus* muscleblind-like 2 (Mbnl2), transcript variant 1, mRNA [NM_175341]

Table S9. Common differentially expressed genes identified in the human multiple sclerosis and in the *corpus callosum* analyses.

Symbol	Gene name
Tmem125	transmembrane protein 125
Prima1	proline rich membrane anchor 1
Gjc2	gap junction protein, gamma 2
Nacad	NAC alpha domain containing
Mapk8ip1	mitogen-activated protein kinase 8 interacting protein 1
Synj2	synaptojanin 2
Gal3st1	galactose-3-O-sulfotransferase 1
Cntn2	contactin 2
Cdk18	cyclin-dependent kinase 18
Mbp	myelin basic protein
Plekhh1	pleckstrin homology domain containing, family H (with MyTH4 domain) member 1
Rasgrp3	RAS, guanyl releasing protein 3
Aplp1	amyloid beta (A4) precursor-like protein 1

Table S10. Common differentially expressed genes identified in the human multiple sclerosis analysis, in the 4-week *corpus callosum* analysis and in the oligodendrocyte progenitor cells analysis.

Symbol	Description
Synj2	*Mus musculus* synaptojanin 2 (Synj2), transcript variant 3, mRNA [NM_011523]
Gjc2	*Mus musculus* gap junction protein, gamma 2 (Gjc2), transcript variant 2, mRNA [NM_175452]
Cdk18	*Mus musculus* cyclin-dependent kinase 18 (Cdk18), mRNA [NM_008795]
Prima1	*Mus musculus* proline rich membrane anchor 1 (Prima1), mRNA [NM_133364]
Gal3st1	*Mus musculus* galactose-3-O-sulfotransferase 1 (Gal3st1), mRNA [NM_016922]
Rasgrp3	*Mus musculus* RAS, guanyl releasing protein 3 (Rasgrp3), transcript variant 2, mRNA [NM_207246]

Tmem125	*Mus musculus* transmembrane protein 125 (Tmem125), mRNA [NM_172383]
Plekhh1	*Mus musculus* pleckstrin homology domain containing, family H (with MyTH4 domain) member 1 (Plekhh1), mRNA [NM_181073]
Cntn2	*Mus musculus* contactin 2 (Cntn2), mRNA [NM_177129]
Mbp	*Mus musculus* myelin basic protein (Mbp), transcript variant 7, mRNA [NM_010777]
Nacad	*Mus musculus* NAC alpha domain containing (Nacad), mRNA [NM_001081652]

Table S11. Differentially expressed genes in *corpus callosum* from mice which underwent remyelination compared to mice treated with cuprizone for 2 weeks.

Symbol	Gene Name	Log2 fold change	p-value	Adjusted p-value
D16Ertd472e	DNA segment, Chr 16, ERATODoi 472, expressed	1.97	4.11E-06	2.74E-02
Aoc1	amine oxidase, copper-containing 1	1.66	4.95E-06	2.74E-02
Krt15	keratin 15	1.76	6.14E-06	2.74E-02
Serpinb1c	serine (or cysteine) peptidase inhibitor, clade B, member 1c	1.92	7.42E-06	2.74E-02
Slain1	SLAIN motif family, member 1	2.02	1.05E-05	2.74E-02
Cxcl10	chemokine (C-X-C motif) ligand 10	1.3	1.35E-05	2.74E-02
Sntn	sentan, cilia apical structure protein	1.63	1.36E-05	2.74E-02
Lrig3	leucine-rich repeats and immunoglobulin-like domains 3	1.56	1.39E-05	2.74E-02
Ppfibp2	PTPRF interacting protein, bindingprotein 2 (liprin beta 2)	1.95	1.58E-05	2.74E-02
Dusp15	dual specificity phosphatase-like 15	1.29	1.65E-05	2.74E-02
Gng11	guanine nucleotide binding protein(G protein), gamma 11	2.07	1.65E-05	2.74E-02
Pllp	plasma membrane proteolipid	1.34	1.71E-05	2.74E-02
Wnt3	wingless-type MMTV integrationsite family, member 3	2.03	1.73E-05	2.74E-02
Mcam	melanoma cell adhesion molecule	1.47	1.74E-05	2.74E-02
D16Ertd472e	DNA segment, Chr 16, ERATO Doi 472, expressed	1.96	1.77E-05	2.74E-02
Creb5	cAMP responsive element binding protein 5	1.47	1.97E-05	2.74E-02
Insc	INSC spindle orientation adaptorprotein	2.11	2.33E-05	2.74E-02
Bfsp2	beaded filament structural protein 2, phakinin	2.08	2.43E-05	2.74E-02
Pls1	plastin 1 (I-isoform)	2.26	2.74E-05	2.74E-02
Otud7b	OTU domain containing 7B	1.56	2.93E-05	2.74E-02
St6galnac3	ST6 (alpha-N-acetyl-neuraminyl-2,3-beta-galactosyl-1,3)-N- acetylgalactosaminide alpha-2,6-sialyltransferase 3	1.74	3.07E-05	2.74E-02
Plekhg3	pleckstrin homology domain containing, family G (with RhoGef domain) member 3	2.64	3.90E-05	2.74E-02

P3h4	prolyl 3-hydroxylase familymember 4 (non-enzymatic)	1.95	4.06E-05	2.74E-02
P3h4	prolyl 3-hydroxylase familymember 4 (non-enzymatic)	1.61	4.07E-05	2.74E-02
Slc4a2	solute carrier family 4 (anionexchanger), member 2	1.78	4.13E-05	2.74E-02
Ifit3	interferon-induced protein withtetratricopeptide repeats 3	1.15	4.40E-05	2.74E-02
Sox8	SRY (sex determining region Y)-box 8	1.49	4.55E-05	2.74E-02
Plaat3	phospholipase A and acyltransferase 3	2.05	4.58E-05	2.74E-02
Sox10	SRY (sex determining region Y)-box 10	1.38	4.64E-05	2.74E-02
Sox10	SRY (sex determining region Y)-box 10	1.96	4.71E-05	2.74E-02
Elovl7	ELOVL family member 7, elongation of long chain fatty acids(yeast)	2.22	4.71E-05	2.74E-02
Plekhh1	pleckstrin homology domain containing, family H (with MyTH4domain) member 1	1.63	4.81E-05	2.74E-02
Lrrc74b	leucine rich repeat containing 74B	1.62	4.85E-05	2.74E-02
Emilin3	elastin microfibril interfacer 3	1.58	4.90E-05	2.74E-02
Prkcq	protein kinase C, theta	1.7	5.07E-05	2.74E-02
Tnfaip6	tumor necrosis factor alpha induced protein 6	2.38	5.08E-05	2.74E-02
Gab1	growth factor receptor boundprotein 2-associated protein 1	1.58	5.21E-05	2.74E-02
Bfsp2	beaded filament structural protein 2, phakinin	1.82	5.32E-05	2.74E-02
Uox	urate oxidase	1.53	5.34E-05	2.74E-02
Il12rb1	interleukin 12 receptor, beta 1	2.51	5.37E-05	2.74E-02
Gjb1	gap junction protein, beta 1	1.84	5.50E-05	2.74E-02
Ppfibp2	PTPRF interacting protein, bindingprotein 2 (liprin beta 2)	1.85	5.69E-05	2.74E-02
Elovl7	ELOVL family member 7, elongation of long chain fatty acids (yeast)	2.35	6.07E-05	2.74E-02
Dock5	dedicator of cytokinesis 5	2.27	6.21E-05	2.74E-02
Tmem163	transmembrane protein 163	1.69	6.27E-05	2.74E-02
Fkbp5	FK506 binding protein 5	-1.76	6.39E-05	2.74E-02
Gsn	gelsolin	2.45	6.54E-05	2.74E-02
Mrgprf	MAS-related GPR, member F	1.36	6.66E-05	2.74E-02
Sema6a	sema domain, transmembrane domain (TM), and cytoplasmic domain, (semaphorin) 6A	1.9	6.77E-05	2.74E-02
B3gnt9	UDP-GlcNAc:betaGal beta-1,3-N-acetylglucosaminyltransferase 9	2.35	6.83E-05	2.74E-02
Emilin2	elastin microfibril interfacer 2	1.34	6.83E-05	2.74E-02
Prkcq	protein kinase C, theta	2.15	6.84E-05	2.74E-02
Cnksr3	Cnksr family member 3	1.37	7.29E-05	2.74E-02

Cd8b1	CD8 antigen, beta chain 1	1.55	7.33E-05	2.74E-02
Cd59a	CD59a antigen	1.51	7.37E-05	2.74E-02
Car2	carbonic anhydrase 2	2.57	7.44E-05	2.74E-02
Iigp1	interferon inducible GTPase 1	1.21	7.50E-05	2.74E-02
Rgs3	regulator of G-protein signaling 3	1.67	7.56E-05	2.74E-02
Serinc5	serine incorporator 5	1.57	7.80E-05	2.74E-02
Rgs3	regulator of G-protein signaling 3	1.14	7.89E-05	2.74E-02
Rnf43	ring finger protein 43	1.62	8.05E-05	2.74E-02
Enpp6	ectonucleotide pyrophosphatase/phosphodiesterase6	1.67	8.07E-05	2.74E-02
Kif13a	kinesin family member 13A	1.02	8.08E-05	2.74E-02
D16Ertd472e	DNA segment, Chr 16, ERATO Doi 472, expressed	1.56	8.13E-05	2.74E-02
Enpp3	ectonucleotide pyrophosphatase/phosphodiesterase3	1.04	8.15E-05	2.74E-02
Unc5b	unc-5 netrin receptor B	2.39	8.25E-05	2.74E-02
Klhl4	kelch-like 4	1.65	8.27E-05	2.74E-02
Trim59	tripartite motif-containing 59	2.54	8.30E-05	2.74E-02
Gpr37	G protein-coupled receptor 37	2.48	8.40E-05	2.74E-02
Insc	INSC spindle orientation adaptor protein	2.39	8.40E-05	2.74E-02
E330034G19Rik	RIKEN cDNA E330034G19 gene	1.26	8.43E-05	2.74E-02
Bche	butyrylcholinesterase	1.78	8.56E-05	2.74E-02
Gramd3	GRAM domain containing 3	1.3	8.67E-05	2.74E-02
Lpar1	lysophosphatidic acid receptor 1	2.77	8.76E-05	2.74E-02
5031410I06Rik	RIKEN cDNA 5031410I06 gene	1.27	9.25E-05	2.74E-02
Plekhh1	pleckstrin homology domain containing, family H (with MyTH4domain) member 1	2.56	9.36E-05	2.74E-02
Fgfr2	fibroblast growth factor receptor 2	1.37	9.34E-05	2.74E-02
Rhobtb3	Rho-related BTB domain containing 3	1.44	9.44E-05	2.74E-02
Nipal4	NIPA-like domain containing 4	2.22	9.46E-05	2.74E-02
Chdh	choline dehydrogenase	1.45	9.47E-05	2.74E-02
Gtsf1l	gametocyte specific factor 1-like	1.38	9.48E-05	2.74E-02
Efhd1	EF hand domain containing 1	1.89	9.53E-05	2.74E-02
Ado	2-aminoethanethiol (cysteamine) dioxygenase	1.36	9.55E-05	2.74E-02
Trp53bp2	transformation related protein 53binding protein 2	1.25	9.61E-05	2.74E-02
Lgals2	lectin, galactose-binding, soluble 2	1.3	9.63E-05	2.74E-02
Aif1l	allograft inflammatory factor 1-like	1.85	9.70E-05	2.74E-02
Sspn	sarcospan	1.55	9.77E-05	2.74E-02
Tmem98	transmembrane protein 98	2.61	9.88E-05	2.74E-02
E030046B03Rik	RIKEN cDNA E030046B03 gene	1.2	1.00E-04	2.75E-02
Cryab	crystallin, alpha B	2.2	1.04E-04	2.81E-02
Arhgef10	Rho guanine nucleotide exchange factor (GEF) 10	1.63	1.09E-04	2.81E-02

Eml1	echinoderm microtubule associated protein like 1	1.4	1.10E-04	2.81E-02
Gjc3	gap junction protein, gamma 3	1.96	1.11E-04	2.81E-02
Nkg7	natural killer cell group 7 sequence	1.22	1.14E-04	2.81E-02
Btd	biotinidase	1.18	1.14E-04	2.81E-02
Gprc5b	G protein-coupled receptor, familyC, group 5, member B	1.54	1.14E-04	2.81E-02
Arrdc3	arrestin domain containing 3	1.49	1.17E-04	2.81E-02
Tmcc3	transmembrane and coiled coil domains 3	1.76	1.18E-04	2.81E-02
Enpp4	ectonucleotide pyrophosphatase/phosphodiesterase 4	1.55	1.19E-04	2.81E-02
Thbs4	thrombospondin 4	1.56	1.20E-04	2.81E-02
St18	suppression of tumorigenicity 18	1.58	1.20E-04	2.81E-02
Plekhb1	pleckstrin homology domain containing, family B (evectins)member 1	2.38	1.24E-04	2.81E-02
Adamts4	a disintegrin-like and metallopeptidase (reprolysin type) with thrombospondin type 1 motif,4	2.2	1.24E-04	2.81E-02
Fam183b	family with sequence similarity 183, member B	1.72	1.25E-04	2.81E-02
Cxcr6	chemokine (C-X-C motif) receptor 6	1.19	1.26E-04	2.81E-02
Fa2h	fatty acid 2-hydroxylase	3.35	1.28E-04	2.81E-02
Ermp1	endoplasmic reticulum metallopeptidase 1	1.34	1.28E-04	2.81E-02
Prrg1	proline rich Gla (G- carboxyglutamic acid) 1	2.07	1.30E-04	2.81E-02
Sirt2	sirtuin 2	1.33	1.35E-04	2.84E-02
Rftn2	raftlin family member 2	1.16	1.36E-04	2.84E-02
Phldb1	pleckstrin homology like domain,family B, member 1	2.5	1.38E-04	2.85E-02
Rnf122	ring finger protein 122	1.77	1.41E-04	2.87E-02
Ddc	dopa decarboxylase	1.98	1.41E-04	2.87E-02
Fnbp1	formin binding protein 1	1.59	1.45E-04	2.88E-02
Slc44a1	solute carrier family 44, member 1	2.5	1.49E-04	2.88E-02
Tmbim1	transmembrane BAX inhibitor motif containing 1	1.51	1.49E-04	2.88E-02
1600029I14Rik	RIKEN cDNA 1600029I14 gene	2.31	1.52E-04	2.88E-02
Crnde	colorectal neoplasia differentiallyexpressed (non-protein coding)	1.23	1.53E-04	2.88E-02
Scd2	stearoyl-Coenzyme A desaturase 2	1.2	1.53E-04	2.88E-02
Ugt8a	UDP galactosyltransferase 8A	3.24	1.54E-04	2.88E-02
Msx1	msh homeobox 1	1.3	1.56E-04	2.88E-02
Fbxo32	F-box protein 32	1.47	1.56E-04	2.88E-02
Tppp3	tubulin polymerization-promotingprotein family member 3	2.41	1.57E-04	2.88E-02
Lrrc1	leucine rich repeat containing 1	1.22	1.58E-04	2.88E-02
Ephb1	Eph receptor B1	1.15	1.58E-04	2.88E-02

Carhsp1	calcium regulated heat stable protein 1	1.92	1.59E-04	2.88E-02
Tnni1	troponin I, skeletal, slow 1	1.86	1.61E-04	2.88E-02
Fah	fumarylacetoacetate hydrolase	1.84	1.62E-04	2.88E-02
Plpp2	phospholipid phosphatase 2	1.19	1.70E-04	2.98E-02
Sh3tc2	SH3 domain and tetratricopeptiderepeats 2	1.58	1.71E-04	2.98E-02
Pde8a	phosphodiesterase 8A	2.51	1.73E-04	2.98E-02
Mkrn3	makorin, ring finger protein, 3	1.23	1.73E-04	2.98E-02
Slc12a2	solute carrier family 12, member 2	1.76	1.75E-04	2.98E-02
Oxsr1	oxidative-stress responsive 1	1.24	1.76E-04	2.98E-02
Dbndd2	dysbindin (dystrobrevin bindingprotein 1) domain containing 2	1.67	1.83E-04	2.98E-02
Erich3	glutamate rich 3	1.5	1.84E-04	2.98E-02
Fam216b	family with sequence similarity 216, member B	1.76	1.84E-04	2.98E-02
Olig1	oligodendrocyte transcription factor 1	1.86	1.84E-04	2.98E-02
Tlcd3a	TLC domain containing 3A	1.34	1.87E-04	2.98E-02
Fbxo36	F-box protein 36	1.57	1.87E-04	2.98E-02
Fam166b	family with sequence similarity 166, member B	1.74	1.90E-04	2.98E-02
Jam3	junction adhesion molecule 3	1.68	1.90E-04	2.98E-02
Myo1d	myosin ID	1.7	1.91E-04	2.98E-02
Sox2ot	SOX2 overlapping transcript (non-protein coding)	2.1	1.93E-04	2.98E-02
Cmtm5	CKLF-like MARVEL transmembrane domain containing5	1.99	1.93E-04	2.98E-02
Ncam1	neural cell adhesion molecule 1	1.57	1.92E-04	2.98E-02
Gal3st1	galactose-3-O-sulfotransferase 1	2.44	1.95E-04	2.99E-02
Olfr1341	olfactory receptor 1341	3.32	1.96E-04	2.99E-02
Trim13	tripartite motif-containing 13	1.62	2.01E-04	2.99E-02
Efs	embryonal Fyn-associated substrate	1.12	2.01E-04	2.99E-02
Serpinb1b	serine (or cysteine) peptidase inhibitor, clade B, member 1b	2.13	2.01E-04	2.99E-02
Il17rb	interleukin 17 receptor B	1.86	2.01E-04	2.99E-02
Fhdc1	FH2 domain containing 1	1.18	2.03E-04	2.99E-02
Arsg	arylsulfatase G	2.34	2.06E-04	2.99E-02
Vipr2	vasoactive intestinal peptide receptor 2	1.47	2.05E-04	2.99E-02
A930003A15Rik	RIKEN cDNA A930003A15 gene	1.53	2.05E-04	2.99E-02
Serpind1	serine (or cysteine) peptidaseinhibitor, clade D, member 1	2.69	2.18E-04	3.09E-02
Tprn	taperin	2.07	2.17E-04	3.09E-02
Tmem229a	transmembrane protein 229A	1.29	2.17E-04	3.09E-02
Kcnk13	potassium channel, subfamily K, member 13	1.52	2.21E-04	3.10E-02
1500015L24Rik	RIKEN cDNA 1500015L24 gene	1.45	2.22E-04	3.10E-02
Mobp	myelin-associated oligodendrocytic basic protein	3.39	2.26E-04	3.10E-02

Cd3d	CD3 antigen, delta polypeptide	1.28	2.24E-04	3.10E-02
Smim1	small integral membrane protein 1	1.28	2.26E-04	3.10E-02
Car14	carbonic anhydrase 14	2.66	2.29E-04	3.12E-02
Ccdc33	coiled-coil domain containing 33	1.56	2.36E-04	3.15E-02
Nkain1	Na+/K+ transporting ATPase interacting 1	1.29	2.37E-04	3.15E-02
Rassf9	Ras association (RalGDS/AF-6)domain family (N-terminal) member 9	1.32	2.38E-04	3.15E-02
Hhip	Hedgehog-interacting protein	1.23	2.38E-04	3.15E-02
Apod	apolipoprotein D	2.21	2.40E-04	3.15E-02
Hhatl	hedgehog acyltransferase-like	1.6	2.39E-04	3.15E-02
Gpr62	G protein-coupled receptor 62	2.79	2.41E-04	3.15E-02
Csrp1	cysteine and glycine-rich protein 1	2.08	2.46E-04	3.17E-02
Tjp2	tight junction protein 2	1.23	2.50E-04	3.17E-02
Cnp	2',3'-cyclic nucleotide 3' phosphodiesterase	2.97	2.54E-04	3.17E-02
Olfml1	olfactomedin-like 1	1.62	2.52E-04	3.17E-02
Marchf8	membrane associated ring-CH-type finger 8	1.4	2.53E-04	3.17E-02
Rffl	ring finger and FYVE like domaincontaining protein	1.84	2.53E-04	3.17E-02
Edil3	EGF-like repeats and discoidin I-like domains 3	1.35	2.58E-04	3.18E-02
Gjc2	gap junction protein, gamma 2	2.24	2.61E-04	3.18E-02
Gss	glutathione synthetase	1.31	2.61E-04	3.18E-02
Tmem88b	transmembrane protein 88B	3.02	2.63E-04	3.18E-02
Dhx40	DEAH (Asp-Glu-Ala-His) box polypeptide 40	0.99	2.63E-04	3.18E-02
Fndc11	fibronectin type III domain containing 11	1.41	2.63E-04	3.18E-02
Tsnaxip1	translin-associated factor X (Tsnax)interacting protein 1	0.94	2.66E-04	3.19E-02
Cobll1	Cobl-like 1	1.27	2.67E-04	3.19E-02
Nol3	nucleolar protein 3 (apoptosis repressor with CARD domain)	1.16	2.69E-04	3.19E-02
Psrc1	proline/serine-rich coiled-coil 1	-1.18	2.70E-04	3.19E-02
Cavin3	caveolae associated 3	1.02	2.72E-04	3.20E-02
Cpox	coproporphyrinogen oxidase	1.42	2.75E-04	3.22E-02
Myb	myeloblastosis oncogene	1.48	2.75E-04	3.22E-02
Rnf7	ring finger protein 7	1	2.77E-04	3.22E-02
Usp18	ubiquitin specific peptidase 18	1.5	2.79E-04	3.22E-02
Ms4a4b	membrane-spanning 4-domains,subfamily A, member 4B	1.46	2.85E-04	3.27E-02
Itgb7	integrin beta 7	1	2.87E-04	3.28E-02
Ddr1	discoidin domain receptor family, member 1	1.62	2.92E-04	3.29E-02
Prima1	proline rich membrane anchor 1	1.81	2.92E-04	3.29E-02
Pcolce2	procollagen C-endopeptidase enhancer 2	1.52	2.96E-04	3.30E-02
Plin5	perilipin 5	1.88	3.00E-04	3.30E-02

Mfhas1	malignant fibrous histiocytomaamplified sequence 1	-0.79	3.00E-04	3.30E-02
Apln	apelin	1.04	3.00E-04	3.30E-02
Ak7	adenylate kinase 7	1.4	3.06E-04	3.33E-02
Mag	myelin-associated glycoprotein	3.13	3.10E-04	3.33E-02
Sgk2	serum/glucocorticoid regulated kinase 2	3.36	3.11E-04	3.33E-02
Kank1	KN motif and ankyrin repeat domains 1	1.15	3.09E-04	3.33E-02
Tspan15	tetraspanin 15	1.73	3.09E-04	3.33E-02
Septin4	septin 4	2.34	3.16E-04	3.33E-02
Snx33	sorting nexin 33	1.77	3.14E-04	3.33E-02
Golga7	golgi autoantigen, golgin subfamilya, 7	1.29	3.20E-04	3.35E-02
Ly6g6d	lymphocyte antigen 6 complex, locus G6D	1.51	3.22E-04	3.35E-02
Slc20a2	solute carrier family 20, member 2	1.08	3.23E-04	3.35E-02
Tcf7l2	transcription factor 7 like 2, T cellspecific, HMG box	1.1	3.24E-04	3.35E-02
Abhd4	abhydrolase domain containing 4	0.88	3.28E-04	3.38E-02
Kat2b	K(lysine) acetyltransferase 2B	0.98	3.34E-04	3.43E-02
Qdpr	quinoid dihydropteridine reductase	2.48	3.38E-04	3.44E-02
Fam83d	family with sequence similarity 83,member D	1.04	3.37E-04	3.44E-02
Dnajb2	DnaJ heat shock protein family(Hsp40) member B2	1.7	3.41E-04	3.46E-02
Sort1	sortilin 1	1.04	3.44E-04	3.46E-02
Cilk1	ciliogenesis associated kinase 1	1.37	3.47E-04	3.46E-02
Trim36	tripartite motif-containing 36	2	3.48E-04	3.46E-02
Ctsk	cathepsin K	1.13	3.49E-04	3.47E-02
Nkd1	naked cuticle 1	1.61	3.51E-04	3.47E-02
Cep97	centrosomal protein 97	1.35	3.58E-04	3.51E-02
Sccpdh	saccharopine dehydrogenase (putative)	1.37	3.60E-04	3.51E-02
Ppp1r32	protein phosphatase 1, regulatory	1.71	3.62E-04	3.51E-02
	subunit 32			
Syt9	synaptotagmin IX	1.18	3.67E-04	3.51E-02
Fmnl2	formin-like 2	0.82	3.68E-04	3.51E-02
Lef1	lymphoid enhancer binding factor 1	1.05	3.70E-04	3.52E-02
Tspan2	tetraspanin 2	2.94	3.72E-04	3.52E-02
Gstm7	glutathione S-transferase, mu 7	1.42	3.81E-04	3.59E-02
En2	engrailed 2	1.13	3.82E-04	3.59E-02
Spg20	spastic paraplegia 20, spartin(Troyer syndrome) homolog (human)	1.3	3.85E-04	3.60E-02
Mboat1	membrane bound O-acyltransferasedomain containing 1	2.2	3.88E-04	3.61E-02
Mtarc1	mitochondrial amidoxime reducing component 1	1.39	3.89E-04	3.61E-02

Cyp2j11	cytochrome P450, family 2,subfamily j, polypeptide 11	1.09	3.90E-04	3.61E-02
S1pr5	sphingosine-1-phosphate receptor 5	3.1	3.95E-04	3.61E-02
Ptp4a1	protein tyrosine phosphatase 4a1	0.81	3.94E-04	3.61E-02
Acap1	ArfGAP with coiled-coil, ankyrinrepeat and PH domains 1	0.86	4.00E-04	3.61E-02
Hs3st1	heparan sulfate (glucosamine) 3-O-sulfotransferase 1	1.38	4.01E-04	3.61E-02
Ifit1	interferon-induced protein withtetratricopeptide repeats 1	1.06	4.01E-04	3.61E-02
Bpgm	2,3-bisphosphoglycerate mutase	1.36	4.01E-04	3.61E-02
Padi2	peptidyl arginine deiminase, type II	2.11	4.03E-04	3.62E-02
Ptk2b	PTK2 protein tyrosine kinase 2 beta	-1.51	4.07E-04	3.63E-02
Dusp26	dual specificity phosphatase 26 (putative)	1.09	4.11E-04	3.66E-02
Map4	microtubule-associated protein 4	1.2	4.14E-04	3.67E-02
Slitrk6	SLIT and NTRK-like family, member 6	1.33	4.27E-04	3.72E-02
Stxbp3	syntaxin binding protein 3	1.25	4.29E-04	3.72E-02
Il1rap	interleukin 1 receptor accessory protein	1.57	4.30E-04	3.72E-02
Gm10791	predicted gene 10791	1.28	4.32E-04	3.72E-02
Rgma	repulsive guidance molecule family member A	0.85	4.43E-04	3.76E-02
Spag17	sperm associated antigen 17	1.16	4.44E-04	3.76E-02
Rab33b	RAB33B, member RAS oncogene family	0.92	4.44E-04	3.76E-02
Bicc1	BicC family RNA binding protein 1	1.3	4.45E-04	3.76E-02
Adi1	acireductone dioxygenase 1	1.14	4.46E-04	3.76E-02
Myo6	myosin VI	1	4.52E-04	3.77E-02
Mia	melanoma inhibitory activity	1.97	4.56E-04	3.79E-02
Lims2	LIM and senescent cell antigen like domains 2	1.01	4.55E-04	3.79E-02
Dnah11	dynein, axonemal, heavy chain 11	1.37	4.62E-04	3.81E-02
Serpinb1a	serine (or cysteine) peptidase inhibitor, clade B, member 1a	3.08	4.72E-04	3.84E-02
Cfap61	cilia and flagella associated protein 61	1.21	4.70E-04	3.84E-02
Frmd8	FERM domain containing 8	1.82	4.74E-04	3.84E-02
Selenbp2	selenium binding protein 2	0.97	4.73E-04	3.84E-02
Smad7	SMAD family member 7	1.2	4.74E-04	3.84E-02
Lsm11	U7 snRNP-specific Sm-like protein LSM11	-0.91	4.76E-04	3.84E-02
Tgtp1	T cell specific GTPase 1	1.47	4.78E-04	3.84E-02
Clic4	chloride intracellular channel 4 (mitochondrial)	1.46	4.78E-04	3.84E-02
Elf5	E74-like factor 5	1.26	4.81E-04	3.85E-02
Ift43	intraflagellar transport 43	0.96	4.83E-04	3.85E-02
Sema6d	sema domain, transmembrane domain (TM), and cytoplasmic domain, (semaphorin) 6D	1.17	4.85E-04	3.86E-02

Wscd1	WSC domain containing 1	1.1	4.91E-04	3.89E-02
Fermt2	fermitin family member 2	0.92	4.99E-04	3.91E-02
Kif1c	kinesin family member 1C	1.13	4.99E-04	3.91E-02
Mbp	myelin basic protein	3.06	5.07E-04	3.92E-02
Cdhr4	cadherin-related family member 4	2.38	5.08E-04	3.92E-02
Rdx	radixin	1.44	5.04E-04	3.92E-02
Plekhg1	pleckstrin homology domain containing, family G (with RhoGef domain) member 1	1.83	5.05E-04	3.92E-02
5430414B12Rik	RIKEN cDNA 5430414B12 gene	1.25	5.05E-04	3.92E-02
Fam177a	family with sequence similarity 177, member A	0.98	5.10E-04	3.92E-02
Lzts2	leucine zipper, putative tumor suppressor 2	1.23	5.14E-04	3.94E-02
Enpp2	ectonucleotide pyrophosphatase/phosphodiesterase2	2.75	5.19E-04	3.96E-02
Odf3b	outer dense fiber of sperm tails 3B	1.79	5.28E-04	4.02E-02
Acsl1	acyl-CoA synthetase long-chain family member 1	1.12	5.33E-04	4.04E-02
Nfe2l3	nuclear factor, erythroid derived 2, like 3	1.85	5.37E-04	4.05E-02
Tmem63a	transmembrane protein 63a	2.47	5.42E-04	4.05E-02
Sec14l4	SEC14-like lipid binding 4	1.21	5.39E-04	4.05E-02
Scd1	stearoyl-Coenzyme A desaturase 1	1.77	5.42E-04	4.05E-02
2700046A07Rik	RIKEN cDNA 2700046A07 gene	1.73	5.47E-04	4.06E-02
Rhpn2	rhophilin, Rho GTPase binding protein 2	1.35	5.48E-04	4.06E-02
Cntn2	contactin 2	2.06	5.57E-04	4.08E-02
Pacs2	phosphofurin acidic cluster sorting protein 2	1.88	5.63E-04	4.08E-02
Anln	anillin, actin binding protein	2.54	5.63E-04	4.08E-02
Josd2	Josephin domain containing 2	1.5	5.62E-04	4.08E-02
Cfap157	cilia and flagella associated protein 157	1.31	5.63E-04	4.08E-02
Nkx6-2	NK6 homeobox 2	2.92	5.69E-04	4.08E-02
Mycbpap	MYCBP associated protein	1.48	5.67E-04	4.08E-02
Gstt3	glutathione S-transferase, theta 3	1.48	5.68E-04	4.08E-02
Cdc42ep1	CDC42 effector protein (Rho	1.56	5.69E-04	4.08E-02

	GTPase binding) 1			
Tpm1	tropomyosin 1, alpha	0.99	5.71E-04	4.09E-02
Camk4	calcium/calmodulin-dependent protein kinase IV	-1.24	5.75E-04	4.10E-02
Eci1	enoyl-Coenzyme A delta isomerase 1	0.81	5.77E-04	4.11E-02
Tmeff2	transmembrane protein with EGF- like and two follistatin-like domains 2	1.49	5.80E-04	4.11E-02
1190005I06Rik	RIKEN cDNA 1190005I06 gene	1.22	5.81E-04	4.11E-02
Pde1c	phosphodiesterase 1C	0.85	5.84E-04	4.12E-02
Misp3	MISP family member 3	1.88	5.86E-04	4.12E-02
Galnt6	polypeptide N-acetylgalactosaminyltransferase 6	1.63	5.88E-04	4.12E-02
Efna1	ephrin A1	0.92	5.92E-04	4.14E-02
Rdh5	retinol dehydrogenase 5	1.59	6.01E-04	4.19E-02
Lss	lanosterol synthase	0.93	6.03E-04	4.19E-02
Bcas1	breast carcinoma amplified sequence 1	2.15	6.10E-04	4.20E-02
Cdr2	cerebellar degeneration-related 2	1.42	6.08E-04	4.20E-02
Foxn3	forkhead box N3	1.38	6.09E-04	4.20E-02
Shisa4	shisa family member 4	0.99	6.15E-04	4.22E-02
Ywhaq	tyrosine 3-monooxygenase/tryptophan 5- monooxygenase activation proteintheta	0.89	6.22E-04	4.25E-02
Larp6	La ribonucleoprotein domain family, member 6	1.11	6.25E-04	4.25E-02
1110017D15Rik	RIKEN cDNA 1110017D15 gene	1.5	6.25E-04	4.25E-02
Ifit3b	interferon-induced protein with tetratricopeptide repeats 3B	1.07	6.30E-04	4.26E-02
Ccdc13	coiled-coil domain containing 13	1.66	6.31E-04	4.26E-02
E130308A19Rik	RIKEN cDNA E130308A19 gene	1.15	6.32E-04	4.26E-02
Mob3b	MOB kinase activator 3B	1.48	6.37E-04	4.27E-02
Chn2	chimerin 2	1.24	6.37E-04	4.27E-02
Cyp51	cytochrome P450, family 51	1.06	6.45E-04	4.32E-02
1700094D03Rik	RIKEN cDNA 1700094D03 gene	1.09	6.50E-04	4.33E-02
Nsdhl	NAD(P) dependent steroid dehydrogenase-like	0.93	6.57E-04	4.33E-02
Dusp16	dual specificity phosphatase 16	1.09	6.60E-04	4.33E-02
Pigz	phosphatidylinositol glycan anchorbiosynthesis, class Z	2.39	6.67E-04	4.33E-02
Per2	period circadian clock 2	-1.19	6.67E-04	4.33E-02
Dnaaf1	dynein, axonemal assembly factor 1	1.13	6.68E-04	4.33E-02
Gbp2	guanylate binding protein 2	1.29	6.69E-04	4.33E-02
Cd82	CD82 antigen	2.41	6.74E-04	4.33E-02
Tnfrsf13c	tumor necrosis factor receptorsuperfamily, member 13c	1.03	6.75E-04	4.33E-02
Efcab14	EF-hand calcium binding domain 14	1.16	6.75E-04	4.33E-02
Lbr	lamin B receptor	0.87	6.76E-04	4.33E-02

Slc5a11	solute carrier family 5 (sodium/glucose cotransporter), member 11	2.03	6.80E-04	4.34E-02
Wipi1	WD repeat domain, phosphoinositide interacting 1	1.19	6.82E-04	4.34E-02
Cldn34c1	claudin 34C1	1.07	6.83E-04	4.34E-02
Cyp2j13	cytochrome P450, family 2,subfamily j, polypeptide 13	0.97	6.84E-04	4.34E-02
Nmral1	NmrA-like family domain containing 1	1.98	6.88E-04	4.35E-02
Tmem125	transmembrane protein 125	3.39	6.98E-04	4.35E-02
Ninj2	ninjurin 2	2.21	7.02E-04	4.35E-02
Gphn	gephyrin	1.02	6.96E-04	4.35E-02
Gatm	glycine amidinotransferase (L-arginine:glycine amidinotransferase)	1.9	7.04E-04	4.35E-02
Cpm	carboxypeptidase M	2.04	7.06E-04	4.35E-02
Cers2	ceramide synthase 2	2.01	7.22E-04	4.40E-02
Gbp8	guanylate-binding protein 8	0.87	7.21E-04	4.40E-02
Baiap2	brain-specific angiogenesis inhibitor 1-associated protein 2	-1.05	7.29E-04	4.43E-02
Hepacam	hepatocyte cell adhesion molecule	0.79	7.36E-04	4.43E-02
Clcn5	chloride channel, voltage-sensitive 5	0.93	7.36E-04	4.43E-02
Cdh20	cadherin 20	1.12	7.37E-04	4.43E-02
Aspa	aspartoacylase	3.21	7.40E-04	4.44E-02
Gng8	guanine nucleotide binding protein(G protein), gamma 8	2.5	7.43E-04	4.44E-02
Slc6a9	solute carrier family 6 (neurotransmitter transporter, glycine), member 9	1.54	7.45E-04	4.44E-02
Macrod1	mono-ADP ribosylhydrolase 1	0.94	7.49E-04	4.45E-02
Plp1	proteolipid protein (myelin) 1	3.38	7.71E-04	4.50E-02
Ermn	ermin, ERM-like protein	2.48	7.75E-04	4.50E-02
Zfp474	zinc finger protein 474	1.63	7.69E-04	4.50E-02
Usp54	ubiquitin specific peptidase 54	1.08	7.69E-04	4.50E-02
Fxyd1	FXYD domain-containing ion transport regulator 1	1.42	7.74E-04	4.50E-02
Fam161a	family with sequence similarity 161, member A	1.15	7.75E-04	4.50E-02
Slc35a2	solute carrier family 35 (UDP- galactose transporter), member A2	0.91	7.78E-04	4.51E-02
Mog	myelin oligodendrocyte glycoprotein	3.27	7.82E-04	4.52E-02
Rras2	related RAS viral (r-ras) oncogene 2	0.74	7.86E-04	4.54E-02
Olig2	oligodendrocyte transcription factor 2	1.45	7.93E-04	4.56E-02
Prr18	proline rich 18	3.24	7.98E-04	4.56E-02
Adamtsl1	ADAMTS-like 1	1.19	7.99E-04	4.56E-02
Lrrn1	leucine rich repeat protein 1, neuronal	1.43	8.00E-04	4.56E-02
Clip1	CAP-GLY domain containing	-0.84	8.01E-04	4.56E-02

	linker protein 1			
Srd5a1	steroid 5 alpha-reductase 1	1.61	8.09E-04	4.57E-02
Rsad2	radical S-adenosyl methioninedomain containing 2	1.14	8.11E-04	4.57E-02
Pard3	par-3 family cell polarity regulator	1	8.11E-04	4.57E-02
Gm16124	predicted gene 16124	0.82	8.18E-04	4.59E-02
Cerox1	cytoplasmic endogenous regulatorof oxidative phosphorylation 1	1.05	8.20E-04	4.59E-02
Ccdc146	coiled-coil domain containing 146	1.1	8.21E-04	4.59E-02
Mpzl1	myelin protein zero-like 1	0.93	8.22E-04	4.59E-02
Cyp39a1	cytochrome P450, family 39,subfamily a, polypeptide 1	1.11	8.32E-04	4.63E-02
Lhx1os	LIM homeobox 1, opposite strand	1.46	8.34E-04	4.63E-02
Rcbtb1	regulator of chromosome condensation (RCC1) and BTB (POZ) domain containing protein 1	1.38	8.35E-04	4.63E-02
Fbxo7	F-box protein 7	1.16	8.38E-04	4.64E-02
Efnb3	ephrin B3	2.39	8.45E-04	4.66E-02
Rgs16	regulator of G-protein signaling 16	0.91	8.44E-04	4.66E-02
Akap14	A kinase (PRKA) anchor protein 14	1.39	8.50E-04	4.67E-02
Ak3	adenylate kinase 3	0.73	8.54E-04	4.69E-02
Add2	adducin 2 (beta)	-0.91	8.69E-04	4.75E-02
Litaf	LPS-induced TN factor	1.86	8.76E-04	4.76E-02
H2-Aa	histocompatibility 2, class II antigen A, alpha	1.22	8.76E-04	4.76E-02
Spata24	spermatogenesis associated 24	1.05	8.77E-04	4.76E-02
BC034090	cDNA sequence BC034090	1.19	8.79E-04	4.76E-02
Kazn	kazrin, periplakin interacting protein	1.24	8.85E-04	4.78E-02
Fam107b	family with sequence similarity 107, member B	1.2	8.86E-04	4.78E-02
Cd74	CD74 antigen (invariant polypeptide of major histocompatibility complex, class IIantigen-associated)	1.24	8.92E-04	4.78E-02
Tmprss5	transmembrane protease, serine 5 (spinesin)	2.14	9.02E-04	4.80E-02
Endod1	endonuclease domain containing 1	1.07	8.97E-04	4.80E-02
Mtmr10	myotubularin related protein 10	1.03	9.02E-04	4.80E-02
Piga	phosphatidylinositol glycan anchorbiosynthesis, class A	1.52	9.03E-04	4.80E-02
Lyst	lysosomal trafficking regulator	-0.88	9.14E-04	4.85E-02
Lap3	leucine aminopeptidase 3	1.05	9.18E-04	4.85E-02
Lrrfip1	leucine rich repeat (in FLII) interacting protein 1	-0.94	9.18E-04	4.85E-02
Abhd5	abhydrolase domain containing 5	0.86	9.21E-04	4.85E-02
Rassf8	Ras association (RalGDS/AF-6)domain family (N-terminal) member 8	0.99	9.24E-04	4.86E-02
Casq2	calsequestrin 2	0.9	9.28E-04	4.86E-02

Ppp2r3a	protein phosphatase 2, regulatorysubunit B", alpha	1.06	9.30E-04	4.86E-02
2010001K21Rik	RIKEN cDNA 2010001K21 gene	1.31	9.34E-04	4.86E-02
Cfap70	cilia and flagella associated protein 70	1.26	9.35E-04	4.86E-02
Traip	TRAF-interacting protein	-1.31	9.39E-04	4.86E-02
Lrguk	leucine-rich repeats and guanylatekinase domain containing	0.92	9.40E-04	4.86E-02
Sntb2	syntrophin, basic 2	-1.14	9.54E-04	4.92E-02
Card19	caspase recruitment domain family,member 19	1.28	9.58E-04	4.92E-02
Slc34a3	solute carrier family 34 (sodiumphosphate), member 3	2.15	9.61E-04	4.93E-02
Slc17a6	solute carrier family 17 (sodium-dependent inorganic phosphate cotransporter), member 6	1.22	9.67E-04	4.93E-02
Rarres2	retinoic acid receptor responder(tazarotene induced) 2	1.84	9.75E-04	4.93E-02
Mal	myelin and lymphocyte protein, Tcell differentiation protein	3.67	9.75E-04	4.93E-02
Tjp3	tight junction protein 3	1.26	9.70E-04	4.93E-02
Gkap1	G kinase anchoring protein 1	0.83	9.76E-04	4.93E-02
Pkp4	plakophilin 4	1.3	9.78E-04	4.93E-02
Slit3	slit guidance ligand 3	-1.57	9.85E-04	4.96E-02
St3gal4	ST3 beta-galactoside alpha-2,3-sialyltransferase 4	1	9.91E-04	4.97E-02

Table S 12. Differentially expressed genes in *corpus callosum* from mice which underwent remyelination compared to mice treated with cuprizone for 4 weeks.

Symbol	Gene Name	Log2 fold change	p-value	Adjusted p-value
Ninj2	ninjurin 2	1.86	6.35E-07	4.60E-03
Mog	myelin oligodendrocyte glycoprotein	3.13	7.39E-07	4.60E-03
Cyp3a13	cytochrome P450, family 3,subfamily a, polypeptide 13	1.34	7.49E-07	4.60E-03
Trib3	tribbles pseudokinase 3	-3.16	7.56E-07	4.60E-03
Gm5067	ribosome biogenesis regulatory protein homolog	2.93	1.52E-06	7.10E-03
Sh3gl3	SH3-domain GRB2-like 3	1.87	2.47E-06	7.10E-03
Msmo1	methylsterol monoxygenase 1	1.47	2.58E-06	7.10E-03
E330037M01Rik	RIKEN cDNA E330037M01 gene	3.05	2.84E-06	7.10E-03
Plekhh1	pleckstrin homology domain containing, family H (with MyTH4domain) member 1	2.28	2.93E-06	7.10E-03
Insig1	insulin induced gene 1	1.39	3.13E-06	7.10E-03
Col11a2	collagen, type XI, alpha 2	1.42	3.21E-06	7.10E-03
Ccp110	centriolar coiled coil protein 110	1.52	3.73E-06	7.58E-03
B230206H07Rik	RIKEN cDNA B230206H07 gene	1.6	4.85E-06	8.28E-03

Speer4cos	spermatogenesis associated glutamate (E)-rich protein 4C, opposite strand transcript	2.03	5.67E-06	8.28E-03
Nupr1	nuclear protein transcription regulator 1	-2.91	6.28E-06	8.28E-03
Mal	myelin and lymphocyte protein, Tcell differentiation protein	3.82	7.10E-06	8.28E-03
Sgk2	serum/glucocorticoid regulated kinase 2	3.07	7.23E-06	8.28E-03
Piga	phosphatidylinositol glycan anchorbiosynthesis, class A	1.48	7.31E-06	8.28E-03
Fa2h	fatty acid 2-hydroxylase	3.3	7.94E-06	8.28E-03
Zdhhc9	zinc finger, DHHC domain containing 9	1.35	8.24E-06	8.28E-03
Ano4	anoctamin 4	0.98	8.30E-06	8.28E-03
Carns1	carnosine synthase 1	1.42	8.46E-06	8.28E-03
Anln	anillin, actin binding protein	2.54	8.58E-06	8.28E-03
Plekhg3	pleckstrin homology domain containing, family G (with RhoGef domain) member 3	2.37	8.70E-06	8.28E-03
Nipal4	NIPA-like domain containing 4	0.74	9.31E-06	8.28E-03
Serpind1	serine (or cysteine) peptidaseinhibitor, clade D, member 1	1.96	1.01E-05	8.28E-03
Adamtsl4	ADAMTS-like 4	1.08	1.02E-05	8.28E-03
Jam3	junction adhesion molecule 3	1.48	1.05E-05	8.28E-03
Elovl1	elongation of very long chain fatty acids (FEN1/Elo2, SUR4/Elo3,yeast)-like 1	1.4	1.10E-05	8.28E-03
Sspo	SCO-spondin	1.25	1.13E-05	8.28E-03
Slc6a9	solute carrier family 6 (neurotransmitter transporter,glycine), member 9	1.53	1.13E-05	8.28E-03
Plekhg3	pleckstrin homology domain containing, family G (with RhoGef domain) member 3	1.71	1.24E-05	8.28E-03
Tex52	testis expressed 52	1.16	1.26E-05	8.28E-03
Phldb1	pleckstrin homology like domain,family B, member 1	2.37	1.27E-05	8.28E-03
Cpox	coproporphyrinogen oxidase	1.31	1.32E-05	8.28E-03
Eif4ebp1	eukaryotic translation initiationfactor 4E binding protein 1	-1.67	1.33E-05	8.28E-03
Septin4	septin 4	2.41	1.41E-05	8.48E-03
Ptprd	protein tyrosine phosphatase, receptor type, D	1.37	1.44E-05	8.48E-03
Dock5	dedicator of cytokinesis 5	1.99	1.46E-05	8.48E-03
Aspa	aspartoacylase	2.28	1.54E-05	8.70E-03
Erbb3	erb-b2 receptor tyrosine kinase 3	0.89	1.66E-05	8.76E-03
Aatk	apoptosis-associated tyrosine kinase	1.34	1.74E-05	9.01E-03
Gstp1	glutathione S-transferase, pi 1	0.98	1.92E-05	9.32E-03
Sox8	SRY (sex determining region Y)-box 8	1.54	1.94E-05	9.32E-03
Rffl	ring finger and FYVE like domaincontaining protein	1.32	1.95E-05	9.32E-03
Map7	microtubule-associated protein 7	1.39	2.06E-05	9.58E-03
Plxnb3	plexin B3	2.4	2.09E-05	9.58E-03
Rcbtb1	regulator of chromosome condensation (RCC1) and BTB (POZ) domain containing protein 1	1.46	2.26E-05	1.02E-02
Plaat3	phospholipase A and acyltransferase 3	2.17	2.37E-05	1.05E-02
Trp53inp2	transformation related protein 53inducible nuclear protein 2	1.19	2.40E-05	1.05E-02
Il17rb	interleukin 17 receptor B	1.25	2.49E-05	1.05E-02
Fign	fidgetin	1.42	2.49E-05	1.05E-02
Opalin	oligodendrocytic myelin paranodaland inner loop protein	1.3	2.58E-05	1.06E-02
Josd2	Josephin domain containing 2	1.3	2.61E-05	1.06E-02
Ugt8a	UDP galactosyltransferase 8A	2.79	2.85E-05	1.14E-02
Pacs2	phosphofurin acidic cluster sorting protein 2	1.93	2.98E-05	1.17E-02

Adamts4	a disintegrin-like and metallopeptidase (reprolysin type) with thrombospondin type 1 motif,4	1.81	3.20E-05	1.24E-02
A730008I21Rik	RIKEN cDNA A730008I21 gene	0.83	3.30E-05	1.25E-02
Slc34a3	solute carrier family 34 (sodium phosphate), member 3	1.6	3.47E-05	1.28E-02
Tprn	taperin	1.9	3.68E-05	1.32E-02
Atf5	activating transcription factor 5	-2.34	3.88E-05	1.37E-02
Car14	carbonic anhydrase 14	2.1	4.13E-05	1.43E-02
Cntn2	contactin 2	1.82	4.17E-05	1.43E-02
Tmeff2	transmembrane protein with EGF-like and two follistatin-like domains 2	1.26	4.23E-05	1.43E-02
Galnt6	polypeptide N-acetylgalactosaminyltransferase 6	1.2	4.53E-05	1.50E-02
Enpp4	ectonucleotide pyrophosphatase/phosphodiesterase4	1.33	4.75E-05	1.50E-02
Desi1	desumoylating isopeptidase 1	1.5	4.82E-05	1.50E-02
Slc5a11	solute carrier family 5 (sodium/glucose cotransporter), member 11	1.54	4.87E-05	1.50E-02
Rasl12	RAS-like, family 12	0.95	5.04E-05	1.50E-02
Enpp6	ectonucleotide pyrophosphatase/phosphodiesterase6	1.13	5.06E-05	1.50E-02
Speer4b	spermatogenesis associated glutamate (E)-rich protein 4B	0.7	5.11E-05	1.50E-02
Gnb4	guanine nucleotide binding protein(G protein), beta 4	0.81	5.12E-05	1.50E-02
Cacna2d4	calcium channel, voltage- dependent, alpha 2/delta subunit 4	1.75	5.16E-05	1.50E-02
Smad7	SMAD family member 7	1.32	5.24E-05	1.50E-02
Cers2	ceramide synthase 2	2	5.25E-05	1.50E-02
Cpm	carboxypeptidase M	1.94	5.25E-05	1.50E-02
Sox10	SRY (sex determining region Y)-box 10	1.6	5.40E-05	1.50E-02
Dync1li2	dynein, cytoplasmic 1 light intermediate chain 2	0.84	5.44E-05	1.50E-02
Dnajb2	DnaJ heat shock protein family (Hsp40) member B2	1.5	5.58E-05	1.51E-02
Foxn3	forkhead box N3	1.31	5.60E-05	1.51E-02
Fnbp1	formin binding protein 1	1.33	5.64E-05	1.51E-02
Trpv3	transient receptor potential cation channel, subfamily V, member 3	0.68	5.77E-05	1.51E-02
Il12rb1	interleukin 12 receptor, beta 1	1.92	5.81E-05	1.51E-02
Srd5a1	steroid 5 alpha-reductase 1	1.5	5.95E-05	1.51E-02
Ldlr	low density lipoprotein receptor	1.77	5.95E-05	1.51E-02
Elovl7	ELOVL family member 7, elongation of long chain fatty acids (yeast)	1.94	5.96E-05	1.51E-02
Mboat1	membrane bound O-acyltransferase domain containing 1	2.1	6.29E-05	1.55E-02
Rnf13	ring finger protein 13	0.9	6.43E-05	1.56E-02
Rftn1	raftlin lipid raft linker 1	1.42	6.57E-05	1.56E-02
Pde4b	phosphodiesterase 4B, cAMP specific	1.48	6.62E-05	1.56E-02
Otud7b	OTU domain containing 7B	1.43	6.63E-05	1.56E-02
Agpat4	1-acylglycerol-3-phosphate O-acyltransferase 4 (lysophosphatidic acid acyltransferase, delta)	1.08	6.73E-05	1.56E-02
Fndc11	fibronectin type III domain containing 11	1.27	6.80E-05	1.56E-02
Frmd8	FERM domain containing 8	1.74	6.80E-05	1.56E-02
Kndc1	kinase non-catalytic C-lobe domain (KIND) containing 1	1.45	7.19E-05	1.62E-02
Gm4221	predicted gene 4221	0.94	7.20E-05	1.62E-02
Micall1	microtubule associated monooxygenase,	2.08	7.25E-05	1.62E-02

	calponin and LIM domain containing -like 1			
Cdc42ep2	CDC42 effector protein (Rho GTPase binding) 2	1.88	7.42E-05	1.64E-02
Mobp	myelin-associated oligodendrocytic basic protein	3.24	7.66E-05	1.65E-02
Mcam	melanoma cell adhesion molecule	0.75	7.85E-05	1.65E-02
Nsdhl	NAD(P) dependent steroid dehydrogenase-like	1.16	7.92E-05	1.65E-02
Mobp	myelin-associated oligodendrocytic basic protein	3.7	7.95E-05	1.65E-02
Dhcr7	7-dehydrocholesterol reductase	1.1	8.02E-05	1.65E-02
5031439G07Rik	RIKEN cDNA 5031439G07 gene	1.45	8.03E-05	1.65E-02
Edil3	EGF-like repeats and discoidin I-like domains 3	0.81	8.05E-05	1.65E-02
Mcam	melanoma cell adhesion molecule	2.17	8.15E-05	1.65E-02
Ralgds	ral guanine nucleotide dissociation stimulator	1.04	8.31E-05	1.65E-02
Daam1	dishevelled associated activator of morphogenesis 1	1	8.31E-05	1.65E-02
Rhog	ras homolog family member G	1.69	8.40E-05	1.65E-02
Nfasc	neurofascin	1.35	8.42E-05	1.65E-02
Tjp2	tight junction protein 2	1.11	8.50E-05	1.65E-02
Hapln2	hyaluronan and proteoglycan link protein 2	2.3	8.52E-05	1.65E-02
Lpar1	lysophosphatidic acid receptor 1	2.44	8.68E-05	1.65E-02
Nat8	N-acetyltransferase 8 (GCN5-related)	-0.92	8.74E-05	1.65E-02
D16Ertd472e	DNA segment, Chr 16, ERATO Doi 472, expressed	1.73	8.90E-05	1.65E-02
Tmprss5	transmembrane protease, serine 5 (spinesin)	1.66	8.91E-05	1.65E-02
Zfp536	zinc finger protein 536	1.54	9.02E-05	1.65E-02
Pde1c	phosphodiesterase 1C	0.72	9.06E-05	1.65E-02
Usp54	ubiquitin specific peptidase 54	1.55	9.07E-05	1.65E-02
Tlcd3a	TLC domain containing 3A	0.9	9.10E-05	1.65E-02
Myo1d	myosin ID	1.16	9.11E-05	1.65E-02
Gipr	gastric inhibitory polypeptide receptor	0.85	9.22E-05	1.65E-02
Nod1	nucleotide-binding oligomerization domain containing 1	1.3	9.33E-05	1.65E-02
Speer8-ps1	spermatogenesis associated glutamate (E)-rich protein 8, pseudogene 1	0.79	9.38E-05	1.65E-02
Nkain2	Na+/K+ transporting ATPase interacting 2	0.88	9.42E-05	1.65E-02
Pcyt2	phosphate cytidylyltransferase 2, ethanolamine	0.98	9.49E-05	1.65E-02
Arhgef10	Rho guanine nucleotide exchange factor (GEF) 10	1.37	9.55E-05	1.65E-02
Padi2	peptidyl arginine deiminase, type II	2.03	9.66E-05	1.66E-02
Bin1	bridging integrator 1	0.84	1.01E-04	1.69E-02
Secisbp2l	SECIS binding protein 2-like	0.98	1.01E-04	1.69E-02
Pls1	plastin 1 (I-isoform)	2.03	1.02E-04	1.69E-02
Tafa1	TAFA chemokine like family member 1	-0.99	1.04E-04	1.71E-02
Tspan2	tetraspanin 2	2.8	1.05E-04	1.71E-02
Nipa1	non imprinted in Prader-Willi/Angelman syndrome 1 homolog (human)	1.04	1.06E-04	1.71E-02
A1300008O04Rik	RIKEN cDNA A1300008O04 gene	1.83	1.07E-04	1.71E-02
Fasn	fatty acid synthase	0.66	1.08E-04	1.71E-02
Erbin	Erbb2 interacting protein	1.49	1.11E-04	1.74E-02
Stard9	START domain containing 9	0.75	1.11E-04	1.74E-02
Dusp26	dual specificity phosphatase 26 (putative)	0.93	1.11E-04	1.74E-02
Nmral1	NmrA-like family domain	2.08	1.15E-04	1.78E-02

	containing 1			
Cmtm5	CKLF-like MARVEL transmembrane domain containing5	1.83	1.19E-04	1.82E-02
Lctl	lactase-like	0.73	1.20E-04	1.83E-02
Nxph4	neurexophilin 4	0.97	1.23E-04	1.87E-02
Pde8a	phosphodiesterase 8A	1.89	1.26E-04	1.88E-02
Ppfibp2	PTPRF interacting protein, bindingprotein 2 (liprin beta 2)	1.55	1.26E-04	1.88E-02
Cyp51	cytochrome P450, family 51	1.02	1.29E-04	1.90E-02
Cdk18	cyclin-dependent kinase 18	1.33	1.30E-04	1.90E-02
Adssl1	adenylosuccinate synthetase like 1	1.45	1.35E-04	1.92E-02
Prr5l	proline rich 5 like	2.59	1.36E-04	1.92E-02
Gm9895	predicted gene 9895	2.05	1.36E-04	1.92E-02
4930506C21Rik	RIKEN cDNA 4930506C21 gene	0.91	1.38E-04	1.92E-02
Arhgap23	Rho GTPase activating protein 23	1.53	1.38E-04	1.92E-02
Pkp4	plakophilin 4	1.29	1.38E-04	1.92E-02
Marchf8	membrane associated ring-CH-type finger 8	1.21	1.39E-04	1.92E-02
Hmgcs1	3-hydroxy-3-methylglutaryl-Coenzyme A synthase 1	1.19	1.39E-04	1.92E-02
Sc5d	sterol-C5-desaturase	0.77	1.40E-04	1.92E-02
Emilin2	elastin microfibril interfacer 2	1.61	1.41E-04	1.92E-02
Sqle	squalene epoxidase	1.07	1.43E-04	1.95E-02
Rtkn	rhotekin	1	1.45E-04	1.97E-02
Cercam	cerebral endothelial cell adhesion molecule	0.66	1.49E-04	1.99E-02
Itgb4	integrin beta 4	2.84	1.50E-04	1.99E-02
Gab1	growth factor receptor boundprotein 2-associated protein 1	1.12	1.51E-04	1.99E-02
Wnt3	wingless-type MMTV integrationsite family, member 3	1.79	1.54E-04	2.00E-02
Abca2	ATP-binding cassette, sub-familyA (ABC1), member 2	1.67	1.57E-04	2.03E-02
Rap1a	RAS-related protein 1a	1.01	1.62E-04	2.08E-02
Chst3	carbohydrate sulfotransferase 3	1.29	1.64E-04	2.09E-02
Fgfr2	fibroblast growth factor receptor 2	1.38	1.67E-04	2.12E-02
Srpk3	serine/arginine-rich protein specific kinase 3	1.89	1.71E-04	2.14E-02
4930402H24Rik	RIKEN cDNA 4930402H24 gene	0.92	1.72E-04	2.14E-02
Tppp	tubulin polymerization promoting protein	0.77	1.74E-04	2.14E-02
Rasgrp3	RAS, guanyl releasing protein 3	1.14	1.74E-04	2.14E-02
Dbndd2	dysbindin (dystrobrevin bindingprotein 1) domain containing 2	1.5	1.75E-04	2.14E-02
Taldo1	transaldolase 1	0.99	1.75E-04	2.14E-02
Dixdc1	DIX domain containing 1	1.15	1.76E-04	2.14E-02
Kazn	kazrin, periplakin interacting protein	1.09	1.79E-04	2.16E-02
Epn2	epsin 2	0.71	1.80E-04	2.16E-02

Spink8	serine peptidase inhibitor, Kazal type 8	-1.9	1.80E-04	2.16E-02
Chac1	ChaC, cation transport regulator 1	-1.43	1.81E-04	2.16E-02
Cldn11	claudin 11	2.89	1.82E-04	2.16E-02
Scd2	stearoyl-Coenzyme A desaturase 2	1.12	1.84E-04	2.17E-02
Fhdc1	FH2 domain containing 1	1.15	1.86E-04	2.19E-02
Slc26a11	solute carrier family 26, member 11	1.03	1.87E-04	2.19E-02
Sort1	sortilin 1	1.04	1.90E-04	2.20E-02
Bace1	beta-site APP cleaving enzyme 1	0.75	1.91E-04	2.20E-02
Tmem189	transmembrane protein 189	0.91	1.91E-04	2.20E-02
Slc7a5	solute carrier family 7 (cationic amino acid transporter, y+ system), member 5	-1.31	1.92E-04	2.20E-02
Ramp2	receptor (calcitonin) activity modifying protein 2	-0.72	1.94E-04	2.22E-02
Car2	carbonic anhydrase 2	2.1	1.99E-04	2.27E-02
Ypel2	yippee like 2	0.8	2.02E-04	2.29E-02
Atp1b3	ATPase, Na+/K+ transporting, beta 3 polypeptide	0.88	2.05E-04	2.31E-02
Gkap1	G kinase anchoring protein 1	0.84	2.07E-04	2.31E-02
2810410L24Rik	RIKEN cDNA 2810410L24 gene	0.69	2.08E-04	2.31E-02
Tmem63a	transmembrane protein 63a	2.45	2.08E-04	2.31E-02
Litaf	LPS-induced TN factor	1.74	2.16E-04	2.39E-02
Rhou	ras homolog family member U	1.39	2.16E-04	2.39E-02
Gdf15	growth differentiation factor 15	-2.19	2.22E-04	2.43E-02
Plp1	proteolipid protein (myelin) 1	3.36	2.24E-04	2.43E-02
Rhobtb3	Rho-related BTB domain containing 3	1	2.26E-04	2.44E-02
Rtkn2	rhotekin 2	1.6	2.28E-04	2.45E-02
Cdc37l1	cell division cycle 37-like 1	1.25	2.31E-04	2.47E-02
Gpr62	G protein-coupled receptor 62	2.49	2.32E-04	2.47E-02
Adamts2	a disintegrin-like and metallopeptidase (reprolysin type) with thrombospondin type 1 motif,2	0.89	2.39E-04	2.53E-02
Dhcr24	24-dehydrocholesterol reductase	1.14	2.48E-04	2.60E-02
Gss	glutathione synthetase	1.11	2.50E-04	2.61E-02
S1pr5	sphingosine-1-phosphate receptor 5	2.68	2.51E-04	2.61E-02
Shtn1	shootin 1	1.65	2.56E-04	2.65E-02
Fam83d	family with sequence similarity 83, member D	0.81	2.58E-04	2.67E-02
Wrap53	WD repeat containing, antisense to Trp53	0.76	2.69E-04	2.77E-02
Sptbn1	spectrin beta, non-erythrocytic 1	1.56	2.75E-04	2.79E-02
Pigz	phosphatidylinositol glycan anchor biosynthesis, class Z	2.25	2.76E-04	2.79E-02
Lss	lanosterol synthase	0.82	2.78E-04	2.79E-02
Wipi1	WD repeat domain, phosphoinositide interacting 1	1.03	2.79E-04	2.79E-02

Xrcc3	X-ray repair complementing defective repair in Chinese hamster cells 3	1.67	2.80E-04	2.79E-02
Jakmip3	janus kinase and microtubule interacting protein 3	0.95	2.84E-04	2.80E-02
Limch1	LIM and calponin homology domains 1	0.79	2.85E-04	2.80E-02
Olig1	oligodendrocyte transcription factor 1	1.69	2.85E-04	2.80E-02
Speer4a	spermatogenesis associated glutamate (E)-rich protein 4A	0.56	2.87E-04	2.80E-02
Gpt	glutamic pyruvic transaminase, soluble	1.14	2.89E-04	2.80E-02
Chd7	chromodomain helicase DNA binding protein 7	1.07	2.89E-04	2.80E-02
5033421B08Rik	RIKEN cDNA 5033421B08 gene	0.62	2.92E-04	2.80E-02
Mbnl2	muscleblind like splicing factor 2	0.97	2.92E-04	2.80E-02
Gm35438	predicted gene, 35438	0.66	2.92E-04	2.80E-02
Ptprf	protein tyrosine phosphatase, receptor type, F	1.03	2.96E-04	2.80E-02
Mxd4	Max dimerization protein 4	1.29	2.99E-04	2.80E-02
Stxbp3	syntaxin binding protein 3	1.33	2.99E-04	2.80E-02
Hhip	Hedgehog-interacting protein	1.37	2.99E-04	2.80E-02
Hipk2	homeodomain interacting protein kinase 2	0.91	3.01E-04	2.80E-02
Creb5	cAMP responsive element bindingprotein 5	1.34	3.02E-04	2.80E-02
Kcnj16	potassium inwardly-rectifying channel, subfamily J, member 16	-0.84	3.06E-04	2.82E-02
St6galnac3	ST6 (alpha-N-acetyl-neuraminyl-2,3-beta-galactosyl-1,3)-N-acetylgalactosaminide alpha-2,6-sialyltransferase 3	1.01	3.08E-04	2.83E-02
Gamt	guanidinoacetate methyltransferase	2.17	3.11E-04	2.84E-02
Cilk1	ciliogenesis associated kinase 1	0.94	3.16E-04	2.87E-02
Cilk1	ciliogenesis associated kinase 1	1.31	3.18E-04	2.87E-02
2700046A07Rik	RIKEN cDNA 2700046A07 gene	1.49	3.21E-04	2.87E-02
Plekhb1	pleckstrin homology domain containing, family B (evectins) member 1	2.27	3.22E-04	2.87E-02
Lrig3	leucine-rich repeats and immunoglobulin-like domains 3	1.36	3.26E-04	2.88E-02
Tmeff1	transmembrane protein with EGF-like and two follistatin-like domains 1	1.07	3.26E-04	2.88E-02
Ttyh2	tweety family member 2	1.54	3.27E-04	2.88E-02
Abhd17b	abhydrolase domain containing 17B	0.89	3.28E-04	2.88E-02
Tesk2	testis-specific kinase 2	0.95	3.29E-04	2.88E-02
Paqr5	progestin and adipoQ receptor family member V	-0.75	3.30E-04	2.88E-02
Ddc	dopa decarboxylase	1.5	3.31E-04	2.88E-02
Chn2	chimerin 2	1.19	3.38E-04	2.92E-02

Uqcrq	ubiquinol-cytochrome c reductase, complex III subunit VII	-0.55	3.43E-04	2.95E-02
Mindy1	MINDY lysine 48 deubiquitinase 1	0.9	3.44E-04	2.95E-02
Depdc1b	DEP domain containing 1B	1.3	3.47E-04	2.97E-02
Larp6	La ribonucleoprotein domain family, member 6	0.84	3.51E-04	2.98E-02
Wnk1	WNK lysine deficient protein kinase 1	1.04	3.59E-04	3.03E-02
Wfdc18	WAP four-disulfide core domain 18	1.47	3.69E-04	3.09E-02
Atp8a1	ATPase, aminophospholipid transporter (APLT), class I, type 8A, member 1	0.66	3.70E-04	3.09E-02
Smtnl2	smoothelin-like 2	1.2	3.73E-04	3.11E-02
Cth	cystathionase (cystathionine gamma-lyase)	-0.79	3.82E-04	3.15E-02
Hsf5	heat shock transcription factor family member 5	0.65	3.82E-04	3.15E-02
Slc44a1	solute carrier family 44, member 1	2.2	3.83E-04	3.15E-02
Sez6l2	seizure related 6 homolog like 2	0.97	3.85E-04	3.15E-02
Ccdc13	coiled-coil domain containing 13	1.37	3.87E-04	3.16E-02
Ccnjl	cyclin J-like	1.1	3.91E-04	3.17E-02
Eda2r	ectodysplasin A2 receptor	-0.51	3.91E-04	3.17E-02
Rnf43	ring finger protein 43	1.11	3.94E-04	3.18E-02
Klk6	kallikrein related-peptidase 6	2.25	4.02E-04	3.19E-02
Fdps	farnesyl diphosphate synthetase	1.07	4.02E-04	3.19E-02
Prima1	proline rich membrane anchor 1	1.21	4.03E-04	3.19E-02
Efcab14	EF-hand calcium binding domain14	0.89	4.03E-04	3.19E-02
Mbp	myelin basic protein	3	4.08E-04	3.22E-02
Mvd	mevalonate (diphospho) decarboxylase	1.05	4.09E-04	3.22E-02
Fdft1	farnesyl diphosphate farnesyl transferase 1	0.86	4.15E-04	3.26E-02
Kif13a	kinesin family member 13A	1.19	4.21E-04	3.30E-02
Prrg1	proline rich Gla (G-carboxyglutamic acid) 1	1.66	4.23E-04	3.30E-02
Sccpdh	saccharopine dehydrogenase (putative)	1.11	4.27E-04	3.32E-02
Arrdc3	arrestin domain containing 3	1.38	4.29E-04	3.32E-02
Emilin3	elastin microfibril interfacer 3	1.05	4.30E-04	3.32E-02
Shisa4	shisa family member 4	0.89	4.35E-04	3.33E-02
Sesn2	sestrin 2	-1.49	4.37E-04	3.33E-02
Arhgef28	Rho guanine nucleotide exchange factor (GEF) 28	0.94	4.45E-04	3.36E-02
Plcl1	phospholipase C-like 1	1.05	4.45E-04	3.36E-02
Slain1	SLAIN motif family, member 1	1.47	4.48E-04	3.36E-02
Tmcc3	transmembrane and coiled coil domains 3	1.58	4.60E-04	3.41E-02
Rnf7	ring finger protein 7	0.85	4.60E-04	3.41E-02
Septin9	septin 9	-0.78	4.61E-04	3.41E-02
Sirt2	sirtuin 2	1.32	4.66E-04	3.41E-02
Syt11	synaptotagmin XI	0.6	4.66E-04	3.41E-02

Pip5k1b	phosphatidylinositol-4-phosphate5-kinase, type 1 beta	-0.86	4.70E-04	3.41E-02
Scd1	stearoyl-Coenzyme A desaturase 1	1.43	4.74E-04	3.43E-02
Ptpdc1	protein tyrosine phosphatasedomain containing 1	0.94	4.79E-04	3.45E-02
Hsd17b7	hydroxysteroid (17-beta) dehydrogenase 7	0.7	4.84E-04	3.47E-02
Cryab	crystallin, alpha B	1.85	4.88E-04	3.47E-02
Ankrd13a	ankyrin repeat domain 13a	0.94	4.88E-04	3.47E-02
Aplp1	amyloid beta (A4) precursor-like protein 1	0.95	4.89E-04	3.47E-02
Fbln5	fibulin 5	-0.96	4.92E-04	3.48E-02
Zdhhc20	zinc finger, DHHC domain containing 20	0.84	5.00E-04	3.51E-02
Fkbp5	FK506 binding protein 5	-0.69	5.02E-04	3.51E-02
Nectin4	nectin cell adhesion molecule 4	1.37	5.12E-04	3.56E-02
Golga7	golgi autoantigen, golgin subfamily a, 7	1.19	5.16E-04	3.58E-02
Lgi3	leucine-rich repeat LGI family, member 3	1.66	5.19E-04	3.59E-02
Adamtsl1	ADAMTS-like 1	0.5	5.38E-04	3.70E-02
1500015L24Rik	RIKEN cDNA 1500015L24 gene	1.02	5.39E-04	3.70E-02
Ldlrad4	low density lipoprotein receptorclass A domain containing 4	0.77	5.50E-04	3.76E-02
Rab33a	RAB33A, member RAS oncogene family	0.74	5.55E-04	3.79E-02
Emb	embigin	-0.79	5.62E-04	3.83E-02
Tmem88b	transmembrane protein 88B	2.4	5.67E-04	3.84E-02
D630028G08Rik	RIKEN cDNA D630028G08 gene	0.6	5.68E-04	3.84E-02
Vmp1	vacuole membrane protein 1	1.92	5.89E-04	3.97E-02
Slc22a23	solute carrier family 22, member 23	0.61	5.97E-04	4.01E-02
Efhd1	EF hand domain containing 1	2.15	6.01E-04	4.03E-02
Apbb2	amyloid beta (A4) precursor protein-binding, family B, member 2	0.56	6.05E-04	4.05E-02
Cep97	centrosomal protein 97	0.92	6.07E-04	4.05E-02
Il33	interleukin 33	2.42	6.14E-04	4.07E-02
Qdpr	quinoid dihydropteridine reductase	2.45	6.20E-04	4.10E-02
Fam222a	family with sequence similarity 222, member A	1.56	6.27E-04	4.14E-02
Tppp3	tubulin polymerization-promotingprotein family member 3	2.06	6.34E-04	4.16E-02
Gjc2	gap junction protein, gamma 2	1.34	6.38E-04	4.17E-02
Ermp1	endoplasmic reticulum metallopeptidase 1	1.09	6.39E-04	4.17E-02
Snx15	sorting nexin 15	0.79	6.48E-04	4.20E-02
Prr18	proline rich 18	3.17	6.48E-04	4.20E-02
Usp30	ubiquitin specific peptidase 30	0.81	6.53E-04	4.22E-02
Sik1	salt inducible kinase 1	0.82	6.56E-04	4.23E-02
Plk3	polo like kinase 3	0.84	6.58E-04	4.23E-02

Cyth1	cytohesin 1	0.89	6.60E-04	4.23E-02
Tmem163	transmembrane protein 163	1.26	6.69E-04	4.25E-02
Serpinb1c	serine (or cysteine) peptidase inhibitor, clade B, member 1c	1	6.74E-04	4.25E-02
Pkp2	plakophilin 2	-1.26	6.76E-04	4.25E-02
Ipo13	importin 13	0.64	6.76E-04	4.25E-02
Tnfaip6	tumor necrosis factor alpha induced protein 6	1.87	6.78E-04	4.25E-02
Nkain1	Na+/K+ transporting ATPase interacting 1	1.78	6.79E-04	4.25E-02
Prkcq	protein kinase C, theta	1.66	6.80E-04	4.25E-02
Carhsp1	calcium regulated heat stable protein 1	1.78	6.88E-04	4.28E-02
Sstr3	somatostatin receptor 3	0.54	6.89E-04	4.28E-02
Nfe2l3	nuclear factor, erythroid derived 2, like 3	1.03	6.91E-04	4.28E-02
5031410I06Rik	RIKEN cDNA 5031410I06 gene	1.02	6.98E-04	4.30E-02
Gm32450	predicted gene, 32450	0.67	7.03E-04	4.32E-02
Rtn4	reticulon 4	0.73	7.07E-04	4.33E-02
Slc45a3	solute carrier family 45, member 3	0.61	7.28E-04	4.42E-02
Lrrn1	leucine rich repeat protein 1, neuronal	1.32	7.30E-04	4.42E-02
Cst3	cystatin C	-0.71	7.40E-04	4.45E-02
Degs1	delta(4)-desaturase, sphingolipid 1	0.75	7.42E-04	4.45E-02
Trim59	tripartite motif-containing 59	1.36	7.43E-04	4.45E-02
Bicd2	BICD cargo adaptor 2	0.76	7.45E-04	4.45E-02
E130308A19Rik	RIKEN cDNA E130308A19 gene	1.25	7.51E-04	4.47E-02
Bfsp2	beaded filament structural protein 2, phakinin	1.41	7.60E-04	4.51E-02
Serinc5	serine incorporator 5	1.32	7.62E-04	4.51E-02
P3h4	prolyl 3-hydroxylase family member 4 (non-enzymatic)	1.02	7.72E-04	4.56E-02
Lbr	lamin B receptor	0.84	7.75E-04	4.57E-02
Adgrv1	adhesion G protein-coupled receptor V1	1.06	7.79E-04	4.57E-02
Enpp2	ectonucleotide pyrophosphatase/phosphodiesterase 2	0.91	7.80E-04	4.57E-02
Pck2	phosphoenolpyruvate carboxykinase 2 (mitochondrial)	-0.7	7.87E-04	4.61E-02
Ldlrap1	low density lipoprotein receptoradaptor protein 1	1.08	7.93E-04	4.62E-02
Ly6e	lymphocyte antigen 6 complex,locus E	-0.94	7.95E-04	4.62E-02
Gal3st1	galactose-3-O-sulfotransferase 1	1.95	8.02E-04	4.63E-02
Rps18	ribosomal protein S18	-0.51	8.02E-04	4.63E-02
Reep3	receptor accessory protein 3	1	8.18E-04	4.68E-02
Rnf141	ring finger protein 141	0.69	8.22E-04	4.69E-02
Acsl1	acyl-CoA synthetase long-chain family member 1	0.66	8.23E-04	4.69E-02
Fah	fumarylacetoacetate hydrolase	1.42	8.26E-04	4.70E-02

Card19	caspase recruitment domain family, member 19	1.08	8.62E-04	4.87E-02
Gpr37	G protein-coupled receptor 37	1.86	8.75E-04	4.93E-02

Table S13. GO molecular functions of differentially expressed genes identified during remyelination compared to treatment with cuprizone for 4 weeks in *corpus callosum*.

GO ID	Term	Ontology	N	Differentially expressed (DE)	p-value	Adjusted p-value
GO:0003824	Catalytic activity	MF	5717	136	1.07E-11	1.58E-08
GO:0005515	Protein binding	MF	9318	186	8.26E-10	5.49E-07
GO:0005488	Binding	MF	13753	245	3.90E-09	2.32E-06
GO:0043167	Ion binding	MF	5513	121	4.81E-08	2.26E-05
GO:0043168	Anion binding	MF	2730	68	1.80E-06	5.80E-04
GO:0019899	Enzime binding	MF	2366	61	2.30E-06	6.96E-04
GO:0019911	Structural constituent of myelin sheath	MF	10	4	7.51E-06	1.91E-03
GO:0016628	Oxidoreductase activity, acting on the CH-CH group of donors, NAD or NADP as acceptor	MF	24	5	1.81E-05	3.94E-03
GO:0036094	Small molecule binding	MF	2432	58	4.24E-05	7.80E-03
GO:0033218	Amide binding	MF	391	17	4.35E-05	7.91E-03
GO:0050839	Cell adhesion molecule binding	MF	284	14	5.44E-05	9.26E-03
GO:0051020	GTPase binding	MF	523	20	5.74E-05	9.60E-03
GO:0070402	NADPH binding	MF	18	4	1.00E-04	1.48E-02
GO:0000166	Nucleotide binding	MF	2033	49	1.41E-04	1.97E-02
GO:1901265	Nucleoside phosphate binding	MF	2033	49	1.41E-04	1.97E-02
GO:0008092	Cytoskeletal protein binding	MF	980	29	1.41E-04	1.97E-02
GO:0016627	Oxidoreductase activity, acting on the CH-CH group of donors	MF	57	6	1.46E-04	2.01E-02
GO:0016717	Oxidoreductase activity, acting on paired donors, with oxidation of a pair of donors resulting in the reduction of molecular oxygen to two molecules of water	MF	8	3	1.46-04	2.01E-02
GO:0016787	Hydrolase activity	MF	2490	57	1.51E-04	2.07E-02
GO:0001540	Amyloid-beta binding	MF	58	6	1.61E-04	2.19E-02
GO:0042277	Peptide binding	MF	318	14	1.79E-04	2.37E-02
GO:0004312	Fatty acid synthase activity	MF	11	3	4.17E-04	4.50E-02
GO:0016746	Transferase activity, transferring acyl groups	MF	268	12	4.41E-04	4.67E-02

Abbreviations: MF, molecular function

Table S14. CIBERSORTx estimated cell fractions of *corpus callosum* samples.

	Neuron	Astrocyte	Oligodendrocyte	Pericyte	Endothelial cell	OPC
Control 1	0.54	0.24	0.09	0.06	0.04	0.02
Control 2	0.39	0.24	0.19	0.08	0.05	0.04
Cuprizone 1	0.41	0.16	0.38	0.02	0.03	0.00
Cuprizone 2	0.53	0.21	0.20	0.04	0.00	0.02
Cuprizone 3	0.43	0.21	0.26	0.06	0.02	0.03
**Mean Control**	0.47	0.24	0.14	0.07	0.05	0.03
**Mean Cuprizone**	0.46	0.19	0.28	0.04	0.02	0.02

Table S15. Pearson's correlation and root mean squared error (RMSE) between original *corpus callosum* data and the CIBERSORTx estimated mixture.

	Correlation	RMSE
Control 1	0.28	0.99
Control 2	0.52	0.85
Cuprizone 1	0.46	0.90
Cuprizone 2	0.38	0.94
Cuprizone 3	0.49	0.87

Table S16. Potential ligands in microglia that could affect gene expression after cuprizone treatment in OPCs ordered by the Pearson correlation coefficient.

Symbol	Gene name
TFPI	Tissue factor pathway inhibitor
SEMA4D	Semaphorin 4D
GSTP1	Glutathione S-Transferase Pi
SERPINE2	Serpin Family E Member 2
LRPAP1	Low Density Lipoprotein-Receptor Related Protein Associated Protein 1
THBS1	Thrombospondin 1
HSP90B1	Heat Shock Protein 90 Beta Family Member 1
EFNB2	Ephrin B2
F11R	F11 Receptor
C3	Complement C3
EFNB1	Ephrin B1
PF4	Platelet Factor 4
RGMA	Repulsive Guidance Molecule BMP Co-Receptor A
LIPH	Lipase H
ARF1	ADP Ribosylation Factor 1
SERPINE1	Serpin Family E Member 1
PROS1	Protein S
GAS6	Growth Arrest Specific 6
ADAM9	ADAM Metallopeptidase Domain 9
DUSP18	Dual Specificity Phosphatase 18
JAM2	Junctional Adhesion Molecule 2
CORT	Cortistatin
VCAM1	Vascular Cell Adhesion Molecule 1
ADAM15	ADAM Metallopeptidase Domain 15
PTPRC	Protein Tyrosine Phosphatase Receptor Type C
COL2A1	Collagen Type II Alpha 1 Chain
CSF1	Colony Stimulating Factor 1
PSAP	Prosaposin

CLCF1	Cardiotrophin Like Cytokine Factor 1
TNFSF9	TNF Superfamily Member 9
ADAM17	ADAM Metallopeptidase Domain 17
FN1	Fibronectin 1
BMP2	Bone Morphogenetic Protein 2
PLAU	Plasminogen Activator, Urokinase
SPP1	Secreted Phosphoprotein 1
LRRC4B	Leucine Rich Repeat Containing 4B
VEGFA	Vascular Endothelial Growth Factor A
OSM	Oncostatin M
IFNA5	Interferon Alpha 5
IHH	Indian Hedgehog Signaling Molecule
IL6	Interleukin 6
APOE	Apolipoprotein E
IL15	Interleukin 15
NPNT	Nephronectin
TNF	Tumor Necrosis Factor
IGF1	Insulin Like Growth Factor 1
CADM1	Cell Adhesion Molecule 1

Table S17. Mean expression and adjusted p-value of differential expressed ligands inmicroglia from control and cuprizone-treated mice.

Ligand	Name	Mean expression in microglia from control mice	Mean expression in microglia from cuprizone-treated mice	Fold change	Adjusted p-value
PLAU	Plasminogen activator, urokinase	8.69	9.67	1.94	0.052
SPP1	Secreted phosphoprotein 1	5.06	9.84	1.11	0.052

Table S18. Mean expression and adjusted p-value of differential expressed receptors in oligodendrocyte progenitor cells from control and cuprizone-treated mice.

Receptor	Name	Mean expressionin OPCs from control mice	Mean expression in OPCs from cuprizone-treated mice	Fold change	Adjusted p-value
Jam3	junction adhesion molecule 3	15.63	14.65	0.94	5.69e-05
Plxnb2	plexin B2	11.21	12.53	1.12	4.28e-03
Hhip	Hedgehog-interacting protein	14.11	12.72	0.90	4.28e-03
Tnfrsf1a	tumor necrosis factor receptorsuperfamily, member 1a	9.44	11.09	1.17	6.06e-03
Acvr1	activin A receptor, type 1	12.40	13.27	1.07	6.06e-03
Ldlr	low density lipoprotein receptor	12.66	11.05	0.87	7.19e-03
Fgfr2	fibroblast growth factor receptor 2	11.40	10.09	0.89	8.94e-03
Itgb4	integrin beta 4	14.62	12.61	0.86	8.94e-03
Ephb1	Eph receptor B1	14.38	13.59	0.94	1.23e-02
Lrp1	low density lipoprotein receptor-related protein 1	10.34	12.00	1.16	2.06e-02
Lpar1	lysophosphatidic acid receptor 1	12.06	10.77	0.89	2.12e-02
Tyro3	TYRO3 protein tyrosine kinase 3	11.69	10.51	0.90	3.22e-02

Itgb5	integrin beta 5	11.37	12.1<br>1	1.07	3.22e-02
Axl	AXL receptor tyrosine kinase	9.21	11.6<br>1	1.26	4.76e-02
Sorl1	sortilin-related receptor, LDLR class A repeats-containing	13.39	11.8<br>5	0.88	4.76e-02

Table S19. Mean expression and adjusted p-value of differentially expressed target genes in oligodendrocyte progenitor cells from control and cuprizone-treated mice.

Target	Name	Mean expression in OPCs from control mice	Mean expression in OPCs from cuprizone treated mice	Fold change	Adjusted p-value
Gadd45b	growth arrest and DNA-damage-inducible 45 beta	10.84	14.51	1.34	3.32e-05
Hist1h3d		13.77	15.55	1.13	3.32e-05
Bbc3	BCL2 binding component 3	11.33	14.24	1.26	3.61e-05
Tnfrsf12a	tumor necrosis factor receptorsuperfamily, member 12a	9.82	13.34	1.36	7.25e-05
Cdkn1a	cyclin-dependent kinase inhibitor 1A(P21)	9.30	15.17	1.63	1.07e-04
Fosb	FBJ osteosarcoma oncogene B	11.78	14.32	1.22	1.38e-04
Abca1	ATP-binding cassette, sub-family A(ABC1), member 1	11.16	13.40	1.20	2.10e-04
Fam181b	family with sequence similarity 181,member B	8.80	12.59	1.43	2.10e-04
Vgf	VGF nerve growth factor inducible	8.98	11.80	1.31	5.08e-04
Klf4	Kruppel-like factor 4 (gut)	11.08	14.15	1.28	5.08e-04
Kdr	kinase insert domain protein receptor	10.76	8.99	0.84	5.08e-04
Gstp1	glutathione S-transferase, pi 1	15.01	14.19	0.94	6.83e-04
Hexim1	hexamethylene bis-acetamideinducible 1	10.37	11.92	1.15	7.02e-04
Trim47	tripartite motif-containing 47	10.00	12.24	1.22	7.02e-04
Egr2	early growth response 2	10.38	12.85	1.24	7.45e-04
Sgk2	serum/glucocorticoid regulated kinase2	14.82	12.16	0.82	7.79e-04
Ccnd1	cyclin D1	9.92	13.32	1.34	8.81e-04
Il18	interleukin 18	13.55	12.34	0.91	1.00e-03
Hmga1	high mobility group AT-hook 1	12.84	15.50	1.21	1.40e-03
Ppp1r15a	protein phosphatase 1, regulatorysubunit 15A	12.47	15.43	1.24	1.40e-03
Dusp1	dual specificity phosphatase 1	11.41	13.23	1.16	1.57e-03
Egr1	early growth response 1	13.21	15.70	1.19	1.57e-03
Gadd45g	growth arrest and DNA-damage-inducible 45 gamma	11.15	15.24	1.37	1.57e-03
Chpf	chondroitin polymerizing factor	11.33	12.92	1.14	1.57e-03
Rab37	RAB37, member RAS oncogenefamily	11.40	10.28	0.90	1.57e-03
Adamtsl4	ADAMTS-like 4	14.13	11.65	0.82	1.57e-03
Pdgfra	platelet derived growth factorreceptor, alpha polypeptide	9.35	10.82	1.16	1.57e-03
Cntf	ciliary neurotrophic factor	10.32	12.01	1.16	1.57e-03
C1qc	complement component 1, qsubcomponent, C chain	9.57	12.34	1.29	1.57e-03
Hmgcs1	3-hydroxy-3-methylglutaryl-Coenzyme A synthase 1	15.24	13.50	0.89	1.63e-03
Itih3	inter-alpha trypsin inhibitor, heavychain 3	13.02	10.49	0.81	1.66e-03
Smad7	SMAD family member 7	13.14	11.94	0.91	1.75e-03
Nfil3	nuclear factor, interleukin 3, regulated	11.65	13.75	1.18	1.85e-03
Midn	midnolin	9.99	12.55	1.26	1.95e-03
Vim	vimentin	10.19	13.81	1.36	1.99e-03

Tnfrsf1a	tumor necrosis factor receptorsuperfamily, member 1a	9.44	11.09	1.17	2.19e-03
Nrcam	neuronal cell adhesion molecule	10.02	12.56	1.25	2.29e-03
Dhrs3	dehydrogenase/reductase (SDRfamily) member 3	10.19	11.60	1.14	2.60e-03
Traf4	TNF receptor associated factor 4	9.68	12.26	1.27	2.63e-03
Cdh13	cadherin 13	9.18	12.09	1.32	2.63e-03
Ldlr	low density lipoprotein receptor	12.66	11.05	0.87	2.81e-03
S100a10	S100 calcium binding protein A10 (calpactin)	9.46	12.25	1.30	2.86e-03
Pcdhga9	protocadherin gamma subfamily A, 9	10.71	11.93	1.11	2.96e-03
Klf6	Kruppel-like factor 6	12.88	14.53	1.13	3.03e-03
Atf4	activating transcription factor 4	10.68	12.04	1.13	3.03e-03
Serpind1	serine (or cysteine) peptidase inhibitor, clade D, member 1	12.36	10.60	0.86	3.10e-03
Timp3	tissue inhibitor of metalloproteinase 3	10.82	13.12	1.21	3.10e-03
Hist1h2ag		13.02	15.17	1.16	4.15e-03
Tsc22d3	TSC22 domain family, member 3	14.90	13.33	0.89	4.98e-03
Lmna	lamin A	12.29	14.20	1.16	5.01e-03
Gdf15	growth differentiation factor 15	8.91	14.19	1.59	5.01e-03
Odc1	ornithine decarboxylase, structural 1	11.01	12.66	1.15	5.01e-03
Bax	BCL2-associated X protein	13.11	14.07	1.07	5.01e-03
Rps19	ribosomal protein S19	13.44	15.06	1.12	5.01e-03
Socs3	suppressor of cytokine signaling 3	11.13	14.29	1.28	5.05e-03
Synpo	synaptopodin	12.27	11.25	0.92	5.19e-03
Klf10	Kruppel-like factor 10	10.07	11.65	1.16	6.18e-03
Ddc	dopa decarboxylase	13.71	12.80	0.93	6.18e-03
Cdk5r1	cyclin-dependent kinase 5, regulatory subunit 1 (p35)	10.93	12.01	1.10	8.98e-03
Lrp1	low density lipoprotein receptor-related protein 1	10.34	12.00	1.16	9.07e-03
Plin4	perilipin 4	11.79	13.36	1.13	9.07e-03
Ifit3	interferon-induced protein with tetratricopeptide repeats 3	9.33	11.03	1.18	9.17e-03
Myh9	myosin, heavy polypeptide 9, non-muscle	12.76	13.59	1.06	9.36e-03
Fos	FBJ osteosarcoma oncogene	12.59	14.95	1.19	9.80e-03
Ank3	ankyrin 3, epithelial	11.95	10.80	0.90	9.80e-03
Apln	apelin	12.35	11.03	0.89	9.80e-03
Per1	period circadian clock 1	12.20	14.41	1.18	1.00e-02
Tle3	transducin-like enhancer of split 3	13.65	14.54	1.07	1.00e-02
Tap2	transporter 2, ATP-binding cassette, sub-family B (MDR/TAP)	10.53	12.63	1.20	1.09e-02
Atf3	activating transcription factor 3	9.71	13.05	1.34	1.19e-02
Zfp36l1	zinc finger protein 36, C3H type-like 1	9.79	11.55	1.18	1.19e-02
Csrnp1	cysteine-serine-rich nuclear protein 1	11.70	12.93	1.11	1.19e-02
C4b	complement component 4B (Chido blood group)	13.80	15.89	1.15	1.19e-02
Cdk18	cyclin-dependent kinase 18	15.67	14.68	0.94	1.29e-02
Crybg3	beta-gamma crystallin domain containing 3	12.51	11.29	0.90	1.36e-02
Cebpb	CCAAT/enhancer binding protein (C/EBP), beta	9.61	12.35	1.28	1.41e-02
Hist1h4d		11.42	13.42	1.17	1.50e-02
Olig2	oligodendrocyte transcription factor 2	13.02	13.99	1.07	1.50e-02
Stat3	signal transducer and activator of transcription 3	12.83	13.59	1.06	1.76e-02
Sgk1	serum/glucocorticoid regulated kinase 1	13.72	15.42	1.12	1.80e-02
Nfkbia	nuclear factor of kappa light polypeptide gene enhancer in B cellsinhibitor, alpha	13.02	14.38	1.10	1.83e-02
Rab34	RAB34, member RAS oncogene family	10.57	12.47	1.18	1.90e-02
Cd44	CD44 antigen	9.51	10.83	1.14	1.90e-02
Anxa2	annexin A2	11.49	14.06	1.22	1.90e-02
Col1a2	collagen, type I, alpha 2	11.44	12.73	1.11	1.94e-02

Gadd45a	growth arrest and DNA-damage-inducible 45 alpha	10.56	12.64	1.20	2.04e-02
C1qb	complement component 1, q subcomponent, beta polypeptide	9.35	11.19	1.20	2.09e-02
Tgif1	TGFB-induced factor homeobox 1	8.84	12.71	1.44	2.14e-02
Spp1	secreted phosphoprotein 1	9.26	10.42	1.13	2.23e-02
Bcl6	B cell leukemia/lymphoma 6	12.51	13.62	1.09	2.30e-02
Mfge8	milk fat globule-EGF factor 8 protein	13.48	14.49	1.07	2.48e-02
Arhgef10l	Rho guanine nucleotide exchange factor (GEF) 10-like	9.48	11.96	1.26	2.59e-02
Reep1	receptor accessory protein 1	9.76	10.99	1.13	2.59e-02
Ddit4	DNA-damage-inducible transcript 4	14.37	15.55	1.08	2.64e-02
Csf1r	colony stimulating factor 1 receptor	11.50	13.92	1.21	2.67e-02
Trib1	tribbles pseudokinase 1	8.13	11.74	1.44	2.93e-02
Tnfaip3	tumor necrosis factor, alpha-inducedprotein 3	8.94	11.00	1.23	2.97e-02
Cirbp	cold inducible RNA binding protein	12.46	14.16	1.14	2.97e-02
Npepps	aminopeptidase puromycin sensitive	14.04	13.24	0.94	2.97e-02
Jun	jun proto-oncogene	14.96	15.97	1.07	3.20e-02
Ly86	lymphocyte antigen 86	7.52	12.08	1.61	3.48e-02
Mgp	matrix Gla protein	12.43	14.42	1.16	3.67e-02
Malat1	metastasis associated lung adenocarcinoma transcript 1 (non-coding RNA)	12.95	14.16	1.09	4.04e-02
Zeb2	zinc finger E-box binding homeobox 2	10.72	9.59	0.89	4.04e-02
Clic1	chloride intracellular channel 1	10.10	12.23	1.21	4.26e-02
Vcan	versican	8.95	11.37	1.27	4.37e-02
Magi1	membrane associated guanylatekinase, WW and PDZ domain containing 1	13.71	12.88	0.94	4.73e-02
